# Novel *Curcumin Inspired* Bis-Chalcone Promotes Endoplasmic Reticulum Stress and Glioblastoma Neurosphere Cell Death

**DOI:** 10.3390/cancers11030357

**Published:** 2019-03-13

**Authors:** Lorenzo Sansalone, Eduardo A. Veliz, Nadia G. Myrthil, Vasileios Stathias, Winston Walters, Ingrid I. Torrens, Stephan C. Schürer, Steven Vanni, Roger M. Leblanc, Regina M. Graham

**Affiliations:** 1 Department of Chemistry, University of Miami, 1301 Memorial Drive, Coral Gables, FL 33146, USA; lo.sansalone@gmail.com (L.S.); eav10@miami.edu (E.A.V.); rml@miami.edu (R.M.L.); 2 University of Miami Brain Tumor Initiative, Department of Neurosurgery, University of Miami Miller School of Medicine, 1095 NW 14th Terrace, Miami, FL 33136, USA; ngm17@med.miami.edu (N.G.M.); v.stathias@med.miami.edu (V.S.); wwalters@med.miami.edu (W.W.); iit4@miami.edu (I.I.T.); 3 Sylvester Comprehensive Cancer Center, University of Miami Miller School of Medicine, 1475 NW 12th Ave, Miami, FL 33136, USA; sschuerer@med.miami.edu; 4 Center for Therapeutic Innovation, Department of Psychiatry and Behavioral Sciences, University of Miami Miller School of Medicine, 1120 NW 14th St, Miami, FL 33136, USA; 5 Department of Molecular and Cellular Pharmacology, Center for Computational Science, University of Miami Miller School of Medicine, 1120 NW 14th St, Miami, FL 33136, USA; 6 Department of Neurosurgery, University of Miami Miller School of Medicine, 1095 NW 14th Terrace, Miami, FL 33136, USA; Svanni@med.miami.edu

**Keywords:** cancer stem cell, endoplasmic reticulum stress, glioblastoma multiforme, unfolded protein response, curcumin, bis-chalcones, brain tumor, glioblastoma stem cell

## Abstract

Glioblastoma (GBM) has a dismal prognosis and successful elimination of GBM stem cells (GSCs) is a high-priority as these cells are responsible for tumor regrowth following therapy and ultimately patient relapse. Natural products and their derivatives continue to be a source for the development of effective anticancer drugs and have been shown to effectively target pathways necessary for cancer stem cell self-renewal and proliferation. We generated a series of curcumin inspired bis-chalcones and examined their effect in multiple patient-derived GSC lines. Of the 19 compounds synthesized, four analogs robustly induced GSC death in six separate GSC lines, with a half maximal inhibitory concentration (IC50) ranging from 2.7–5.8 μM and significantly reduced GSC neurosphere formation at sub-cytotoxic levels. Structural analysis indicated that the presence of a methoxy group at position 3 of the lateral phenylic appendages was important for activity. Pathway and drug connectivity analysis of gene expression changes in response to treatment with the most active bis-chalcone 4j (the 3,4,5 trimethoxy substituted analog) suggested that the mechanism of action was the induction of endoplasmic reticulum (ER) stress and unfolded protein response (UPR) mediated cell death. This was confirmed by Western blot analysis in which 4j induced robust increases in CHOP, p-jun and caspase 12. The UPR is believed to play a significant role in GBM pathogenesis and resistance to therapy and as such represents a promising therapeutic target.

## 1. Introduction

Glioblastoma (GBM) is one of the worst diagnoses that a person can receive, with an average survival time of approximately 12–15 months [1]. It is the most common and malignant form of primary brain cancer and is currently treated with surgery to remove the tumor followed by radiotherapy with concurrent chemotherapy. It is a highly invasive cancer, invading into the normal tissue surrounding the tumor, making a total resection virtually impossible. Despite an enormous effort to identify the genetic and epigenetic alterations and develop molecularly targeted therapies, the outcome has not significantly improved in over fifteen years since the introduction of the Stupp protocol, which is the addition of the DNA alkylating agent temozolomide to the standard of care, affording patients an additional 2–3 months of survival [2]. Ultimately however, the tumor recurs and the patient succumbs to the disease. Treatment failure has been attributed to the highly invasive nature of these tumors as well as the presence of treatment resistant glioblastoma stem-like cancer cell also known as tumor initiating cells or glioblastoma stem cells (GSCs) [3].

The stem cell theory of carcinogenesis postulates that a small proportion of tumor cells are responsible for driving tumor growth, by giving rise to additional cancer stem cells as well as more differentiated progeny, contributing to tumor heterogeneity. The role of GSCs has been elegantly illustrated in cell lineage studies, which demonstrated the tumor recurrence following treatment was in fact due to GSCs in GBM mouse models [3]. Therefore, for long-term cures, treatments must successfully target GSCs. Multiple signaling pathways have been implicated in GSC self-renewal and therapy-resistance [4]; however, effective clinical therapies remain elusive.

It is now becoming more evident that the adaptive response known as the unfolded protein response (UPR) plays a major role in the development and progression of several cancers including GBM [5,6,7,8]. GBM is a fast growing tumor, often outgrowing its blood supply resulting in hypoxia, nutrient deprivation and acidosis. In addition to these extrinsic stresses, GBM cells are subject to intrinsic stresses including oncogene pathway activation and an increased demand for protein synthesis and folding. The endoplasmic reticulum (ER) plays a critical role in protein folding and secretion; and cellular stress can perturb the normal protein homeostasis leading to the accumulation of unfolded or misfolded proteins. In response to this stress, cells initiate the unfolded protein response (UPR), an adaptive response that aims to restore ER proteostasis. The UPR consists of three signal transduction pathways initiated by ER resident proteins, inositol requiring enzyme 1 (IRE1), double-stranded RNA-activated protein kinase (PKR)–like ER kinase (PERK), and activating transcription factor 6 (ATF6) and activation of these pathways is the attempt to mitigate, and reverse ER stress by promotion of protein degradation, decrease in global protein synthesis and upregulation of the expression of specific UPR downstream genes such as molecular chaperones and foldases to aid in protein folding. However, if the ER stress cannot be resolved, the UPR switches from adaptation and cell survival to the induction of apoptosis and cell death. Specifically, the convergence of the aforementioned pathways is actuated by the multi-functional transcription factor CCAAT-enhancer-binding protein homologous protein (CHOP), which plays a key role in ER stress induced cell death [9]. Evidence is accumulating and suggesting that the UPR plays an important role in GBM growth and progression, and supports tumor cell survival response to radiotherapy and chemotherapy and as such represents a promising therapeutic target [9].

Natural products and their derivatives have long provided a diverse source of new medicinal leads, especially in the development of anti-cancer drugs [10]. One of the most studied natural products is curcumin, which is a bioactive component of the popular Indian spice turmeric. Curcumin has shown anti-cancer properties for multiple cancers including GBM [11,12,13]. Specifically, curcumin has been shown to downregulate many cellular pathways critical for cancer stem cell self-renewal [14]. We previously demonstrated that curcumin caused GSC death by inducing reactive oxygen species (ROS) and downregulation of STAT3 activity [15]. However, a major obstacle to curcumin therapy is its poor bioavailability. Even at high oral doses (8 g/day), curcumin peak plasma levels are below 2 μM [16]. Attempts to increase plasma levels resulted in the development of different formulations including, theracurmin^®^, curcumin within N-trimethyl chitosan coated solid lipid nanoparticles or nano-emulsions encapsulating curcumin which demonstrated to increase peak plasma levels up to 12.6 μM in rodent models [17,18,19]. An alternate approach is to design curcumin structural analogs to optimize specific chemotherapeutic properties. Specifically, chalcones, both synthetic and natural, have demonstrated anti-glioma effects by multiple mechanisms. [20,21,22]. Recently, symmetric bis-chalcones were demonstrated to be potent inhibitors of the breast cancer resistance protein (BCRP/ABCG2); however, the anti-cancer effects of bis-chalcones have not been investigated in GBM [23]. Here, we generated a series of *curcumin inspired* bis-chalcone derivatives and examined their effect on GBM stem cells (GSCs). Patient derived GSCs have been shown to recapitulate the original tumor upon transplantation into mice confirming their reliability as an in vitro model system. [24].

## 2. Results

### 2.1. Bis-Chalcone Synthesis

**Figure 1:**
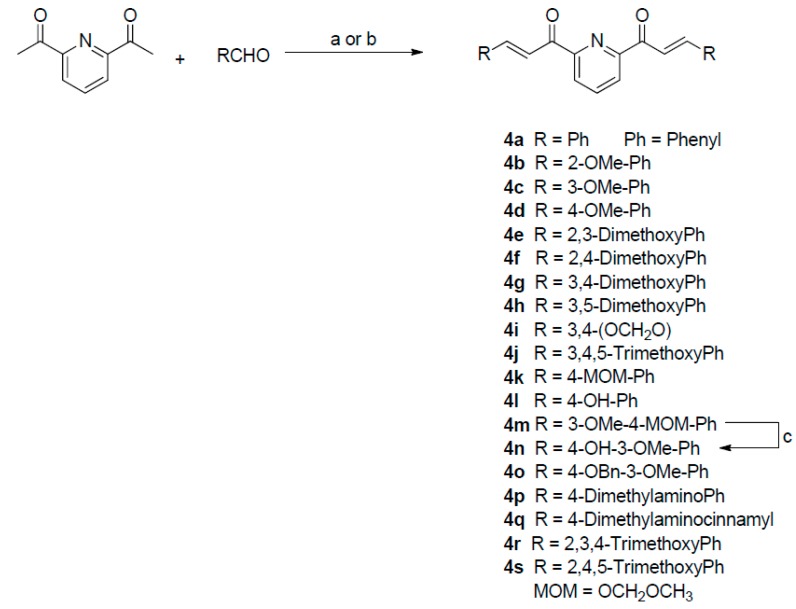
Reaction scheme for the synthesis of bis-chalcones^a^. ^a^Reagents and conditions: (a) 20%NaOH, MeOH, RT; (b) cat. Piperidine, MeOH, ref lux; (c) Trifluoroacetic acid/conc. HCl, Dichloromethane.

The synthesis of bis-chalcones 4a–4s is outlined in the following reaction scheme (Figure 1). The bis-chalcones were prepared by a base-catalyzed Claisen–Schmidt condensation between 2,6-diacetylpyridine (1 equivalent) and the appropriate aryl aldehyde (2.1 equivalents) using either method a or b. Bis-chalcones 4a, 4d, 4f [25], 4g [26] 4l [27] and 4p [28] were previously cited in the literature. More detailed description of the synthesis along with the spectral data for each compound can be found in the experimental section of the Supplemental Materials.

### 2.2. Bis-Chalcones Reduce Viability in GSCs

**Figure 2:**
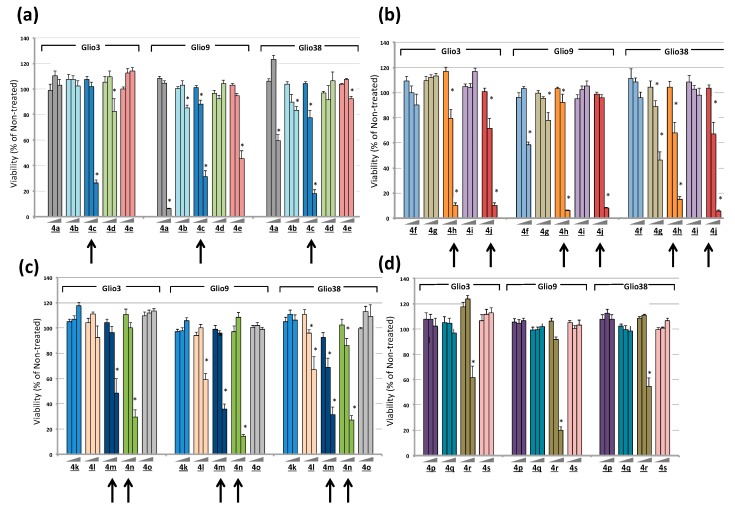
Bis-chalcones reduce GSC viability. GSC lines Glio3, Glio9 and Glio38 were treated with 0.1 μM, 1 μM, or 10 μM of each bis-chalcone analog and viability determined by MTS assay. The data is presented as percent viability compared to non-treated controls. * *p* < 0.05, compared to non-treated controls. Arrows indicate bis-chalcones that reduced viability over 50% at the 10 μM in all three GSC lines. (**a**) bis-chalcones 4a–4e; (**b**) bis-chalcones 4f–4j; (**c**) bis-chalcones 4k–4o; (**d**) bis-chalcones 4p–4s.

We previously found curcumin induced GSC death with an approximate IC_50_ of 25 μM. To determine if these bis-chalcones were more cytotoxic than curcumin, GSC lines Glio3, Glio9 and Glio38 were treated with increasing concentrations of each analog and viability was determined 72 h later by 3-(4,5-dimethylthiazol-2-yl)-5-(3-carboxymethoxyphenyl)-2-(4-sulfophenyl)-2H- tetrazolium) MTS assay. The percent viable cells for concentrations of 0.1 μM, 1 μM and 10 μM are shown in Figure 2. Interestingly, 10 μM of 4a and 4e (Figure 2a) induced robust cell death in Glio9, to approximately 6% and 45% of non-treated cells respectively, but only slightly reduced cell viability in the remaining two cell lines. On the other hand, 4r (Figure 2d) significantly reduced viability in all cell lines, although not all below 50% viability (approximately 20%–62% compared to non-treated controls). Morphological examination of Glio3 (62% viability) suggested that 4r might promote GSC differentiation as well as cell death as indicated by the loss of neurospheres and the corresponding increase in a more differentiated phenotype (Supplementary Figure S1). Bis-chalcone 4g (Figure 2b) reduced viability by more than 50% in Glio38 but was less effective in Glio3 and Glio9. At a concentration of 10 μM, bis-chalcones 4c (Figure 2a: blue), 4h and 4j (Figure 2b: orange and red), 4m and 4n (Figure 2c: dark blue and green) reduced cell viability below 50% compared to non-treated controls (100% viability) in all three GSC lines; Glio3, Glio9 and Glio38 (arrows).

### 2.3. Bis-Chalcones 4c, 4h, 4j and 4n Substantially Reduce Viability in Six GSC Lines

**Figure 3:**
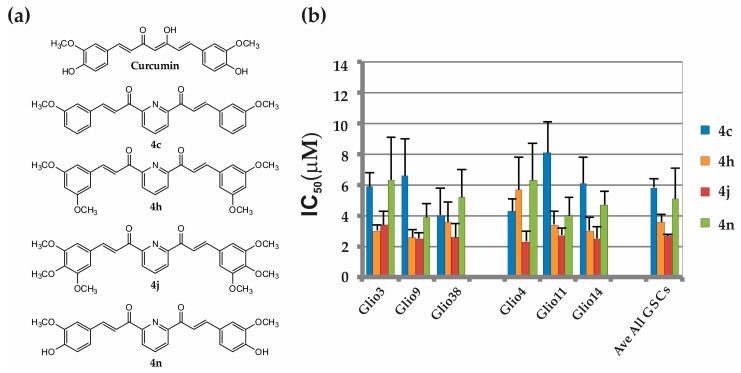
Bis-chalcones 4c, 4h, 4j, 4n induces robust cell death in 6 GSC lines. (**a**) structures of 4c, 4h, 4j, and 4n; (**b**) IC50 of each bis-chalcone for Glio3, Glio4, Glio9, Glio11, Glio14 and Glio38.

Since we are interested in finding an analog that is substantially more potent than curcumin and demonstrates efficacy across multiple GSC lines, we chose to continue further analysis only with the analogs in which upon treatment with 10 μM decreased the viability over 50% in all three cell lines compared to non-treated controls (arrows, Figure 2). To confirm the GSC cytotoxicity of bis-chalcones 4c, 4h, 4j, 4m and 4n, we treated three additional GSC lines, Glio4, Glio11, and Glio14, with increasing concentrations of each analog and determined cell viability. Similar to previous results, 4c, 4h, 4j and 4n induced robust cell death in the three additional GSC lines. The structures and IC50 for these analogs are shown in Figure 3a,b, respectively. Previously, we determined that the IC50s for curcumin were as follows: Glio3 25.5 ± 2.7 μM, Glio4 39.5 ± 5.4 μM, Glio9 22.5 ± 1.7 μM, Glio11 20.3 ± 3.7 μM, and Glio14 13.9 ± 5.0 μM [15]. Overall, on average, these analogs were approximately 5–10 fold more cytotoxic than curcumin, with 4j, the 2,6-di-[3-(3,4,5-trimethoxyphenyl)propenoyl]pyridine, having the lowest IC50. (Glio3 3.37 ± 0.9 μM, Glio4 2.32 ± 0.6 μM, Glio9 2.51 ± 0.4 μM, Glio11 2.73 ± 0.5 μM, and Glio14 2.52 ± 0.8 μM, Glio38 2.58 ± 0.9). Interestingly, 4m induced robust loss of cell viability in two additional cell lines (Glio11 and Glio14) but to a much lesser extent in Glio4. This is consistent with our previous data demonstrating that, among the five GSC lines examined, Glio4 was the most resistant to curcumin. Percent viability and the IC50 of 4m for Glio4, Glio11 and Glio14 are shown in Supplementary Figure S2a,b, respectively. Taken together, these data suggest that the genetic or epigenetic differences between the various GSC lines may regulate the susceptibility to the bis-chalcones.

### 2.4. Bis-Chalcones Reduce Neurosphere Formation at Sub Cytotoxic Levels

**Figure 4:**
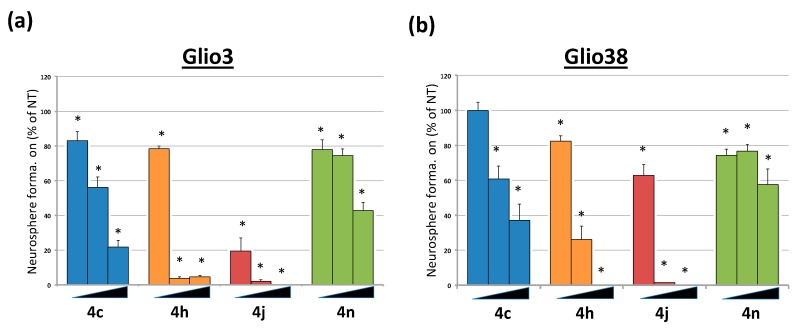
Bis-chalcones 4c, 4h, 4j, 4n significantly reduce neurosphere formation. GSCs Glio3 and Glio38 were plated as single cells in 96-well plates and treated with increasing concentration of 4c, 4h, 4j, 4n (100 nM, 250 nM or 500 nM) and the number of neurospheres counted on day 14. Data is presented as percent neurospheres relative to non-treated controls. (**a**) Glio3; (**b**) Glio38. * *p* < 0.05.

We previously demonstrated that 2.5 μM curcumin, a 10-fold lower dose than the average IC50, could significantly interfere with GSC neurosphere formation, suggesting an inhibition of GSC self-renewal properties [15]. To determine the effect of the bis-chalcones on neurosphere formation, GSC lines, Glio3 and Glio38, were dissociated and 50–100 single cells/well were plated into 96-well plates, treated with each analog at 100 nM, 250 nM and 500 nM concentrations and the number of neurospheres counted 14 days later. Sub cytotoxic levels of each bis-chalcone significantly reduced neurosphere formation in both cell lines (Figure 4). Consistent with the viability results, bis-chalcone 4j was the most effective, virtually eliminating sphere formation at concentrations as low as 250 nM.

### 2.5. Bis-Chalcone 4j Does not Substantially Reduce p-Stat Activity

We and others have shown that curcumin induces anti-cancer effects via STAT3 inhibition [15,29]. To determine if the mechanism of the most active bis-chalcone, 4j, is similar, we investigated the levels of STAT3 phosphorylation at tyrosine 705 (p-STAT3) and total STAT3 in Glio3 and Glio38 in response to either 5 μM bis-chalcone 4j as well as 4c and 4n. However, unlike curcumin, the bis-chalcones did not substantially reduce p-STAT3 levels suggesting an alternate mechanism of action. Bis-chalcones did, however, induce caspase activity, indicating activation of an apoptotic pathway (Supplemental Figure S3).

### 2.6. Bis-Chalcone 4j Induces Gene Expression Changes Consistent with ER Stress and UPR

To more extensively study the mechanism of action of 4j, we proceeded with profiling its transcriptional impact on Glioblastoma cells. For this, the GSC lines Glio9, Glio11, Glio14 and Glio38 were treated with 2.5 μM bis-chalcone 4j or, for comparison, bis-chalcone 4n and curcumin for 24 h and mRNA were collected for gene expression profiling using the L1000 Platform [30].

#### 2.6.1. Bis-Chalcones 4j Induces a Greater Transcriptional Impact Compared to 4n or Curcumin

**Figure 5:**
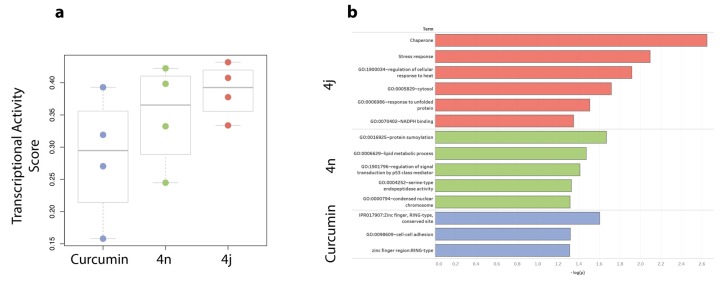
Bis-chalcone 4j elicits a high transcriptional response. (**a**) GSCs Glio9, Glio11, Glio14 and Glio38 were treated with 2.5 μM bis-chalcone 4j, 4n or curcumin for 24 h; RNA was collected and processed by the L1000 Platform. The Transcriptional Activity Score (TAS) for each condition was calculated from the resulted L1000 gene expression data; (**b**) Bis-chalcone 4j induces a transcriptional signature consistent with ER stress. For each compound, a Transcriptional Consensus Signature was calculated from the L1000 gene expression profiling data and subjected to functional enrichment analysis using DAVID.

We then calculated the Transcriptional Activity Score (TAS) for each treatment and compared the results among the different conditions. TAS is a quality metric developed for the L1000 gene expression data that quantifies the strength and reproducibility of the transcriptional changes induced by a perturbation [30]. As shown in Figure 5a, bis-chalcone 4j elicited the strongest transcriptional response among the four GSC lines tested (Average TAS = 0.388). Moreover, curcumin exhibited the lowest transcriptional response among the three compounds with an average TAS of 0.285. By comparing our results to CLUE (https://clue.io), an external L1000 database, we observed that curcumin exhibits a similar weak transcriptional response in non-glioblastoma cancer cell lines (TAS = 0.21).

#### 2.6.2. Bis-Chalcone 4j Induces a Transcriptional Signature Consistent with ER Stress

To identify genes that differentially regulated across all four glioblastoma cell lines, we calculated the Transcriptional Consensus Signature for each compound, as previously described (Table S1 in expression profiling data section of the Supplemental Materials) [31]. The upregulated and downregulated genes of each compound were then used to perform a functional enrichment analysis using the online annotation tool DAVID [32]. As shown in Figure 5b, the transcriptional signature of 4j is enriched in terms related to ER stress response, including “chaperone”, “stress response” and “response to unfolded protein”. No obvious discernable pathway was evident for bis-chalcone 4n or curcumin at the concentration tested.

#### 2.6.3. Bis-Chalcone 4j Induce Similar Transcriptional Responses only in the Neurosphere Cell Lines

**Figure 6:**
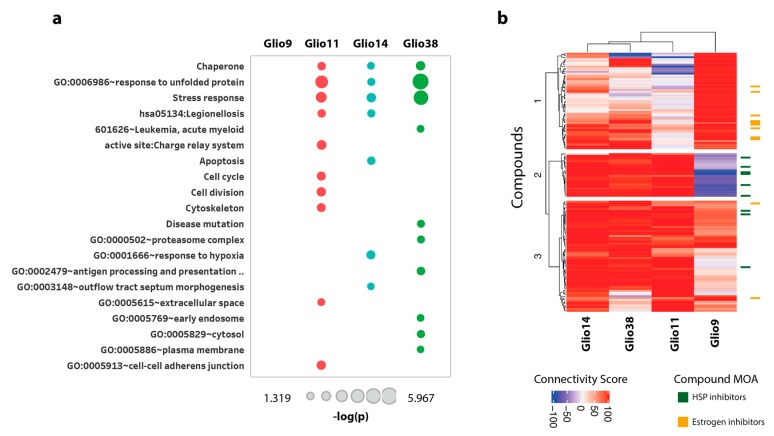
Bis-chalcone 4j induce similar transcriptional responses only in the neurosphere cell lines. (**a**) The individual gene expression signatures for each condition were subjected to functional enrichment analysis using DAVID and the significant (*p*-value < 0.05) enrichment terms were ranked based on the numbers of cells that were enriched in them; (**b**) Drug connectivity analysis of 4j indicates ER perturbation of neurosphere cells. Drug connectivity scores were determined by comparing the 4j, 4n and Curcumin transcriptional response to 2911 compounds, part of the CLUE Touchstone dataset. Hierarchical clustering was performed on the 50 most connected compounds for each condition. Glio11, Glio14 and Glio38 cluster together and are highly connected to heat shock protein HSP inhibitors (green). Glio9 was highly connected to estrogen inhibitors (orange).

The Transcriptional Consensus Signature was generated by aggregating across all four GSC lines; however, we also wanted to evaluate whether there were cell-specific transcriptional responses to the 4j, 4n, and curcumin treatments. For this, we created cell-specific gene expression signatures that would be indicative of a cell’s transcriptional response to a treatment. This signature was created by computing the median gene expression across all biological replicates for a particular GSC line–compound pair. We then performed the same functional enrichment analysis as above (filtered genes with |z-score| >= 1). As shown in Figure 6a, we observed that three (Glio11, Glio14 and Glio38) out of the four cell lines treated with bis-chalcone 4j were enriched in stress response terms similarly to Figure 5b; however, Glio9 followed a different transcriptional response. Interestingly, Glio11, Glio14 and Glio38 were derived from treatment naïve tumors and grow as neurospheres, whereas Glio9 was derived from a recurrent tumor and grows adherently. The functional enrichment analysis for bis-chalcone 4n and curcumin can be found in the expression profiling data of Supplementary Materials.

#### 2.6.4. Drug Connectivity Analysis of 4j Supports ER Perturbation of Neurosphere Cells

We further validated the difference in the transcriptional response of Glio9, Glio11, Glio14 and Glio38 after bis-chalcone 4j treatment by evaluating their connectivity to other compounds in the CLUE reference dataset (Touchstone), a dataset consisting of cellular signatures representing systematic perturbations of small-molecule and genetic perturbations. The cell-specific gene expression signatures were used as input in the CLUE tool and for each signature we extracted the 50 most connected/similar compounds (out of a total of 2911 reference CLUE compounds). We then plotted the corresponding Connectivity Scores in Figure 6b. The differential response to treatments was more prominent in the case of 4j, where we can see that the Glio14, Glio38 and Glio11 signatures are highly connected to compounds in Cluster 2 and 3 and the Glio9 signature is highly connected to compounds in Cluster 1. Moreover, by examining the mechanisms of action of the compounds in each Cluster, we noticed that the Glio14, Glio38 and Glio11 signatures were highly connected to heat shock protein (HSP) inhibitors (Cluster 2), while the Glio9 signature was highly connected to estrogen inhibitors (Cluster 1). Drug connectivity analysis for bis-chalcone 4n and curcumin can be found in the expression profiling data of the Supplementary Materials.

### 2.7. Bis-Chalcone 4j Induces Robust Expression of CHOP and Promotes JNK and Caspase 12 Activity

**Figure 7:**
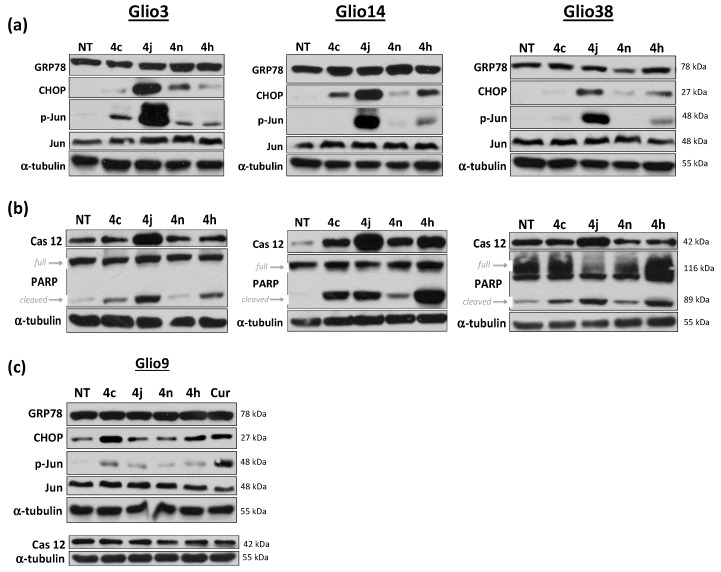
Western blot analysis indicates that 4j induces robust ER stress and UPR in neurosphere cells. Glio3, Glio9, Glio14 and Glio38 were treated with 5 μM of bis-chalcones 4c, 4h, 4j, and 4n for 8 or 24 h and subjected to Western blot analysis. Levels of GRP78, CHOP, p-jun, jun, were examined at 8 h. Levels of caspase 12 and cleaved PARP were examined at 24 h. α-tubulin serves as a loading control. (**a**) levels of ER stress/UPR markers at eight hour in the neurosphere cell lines; (**b**) levels of apoptosis indicators at 24 h in the neurosphere cell lines; (**c**) levels of ER stress/UPR markers in the adherent Glio9 cell line.

The above analysis indicates that 4j cytotoxicity may be mediated through the ER stress induced UPR response. The UPR is an adaptive mechanism initiated in response to ER stress. To confirm that bis-chalcone 4j was inducing the UPR, we examined UPR markers in the GSCs that grow as neurospheres (glio3, 14 and 38) by Western blot analysis. Consistent with the transcriptional response and drug connectivity data, 4j induced robust increase in the protein levels of UPR markers including CHOP, p-jun at serine 73 (indicative of the stress activated kinase c-Jun N-terminal kinase (JNK) activity) and caspase 12 only in the neurosphere cell lines. No obvious increase in glucose related protein 78 (GRP78), a molecular chaperone important for mediating cell adaptation and survival in response to ER stress, was observed (Figure 7a,b). The other analogs examined either failed to induce protein expression or did so at a much lower level compared to 4j, indicating that 4c, 4h, 4n induce GSC death, at least in part, by an alternate mechanism. All analogs however induced Poly (ADP-ribose) polymerase (PARP) cleavage suggestive of apoptotic cell death. As expected, 4j treatment did not result in an increase in CHOP or caspase 12 in Glio9. Bis-chalcone 4j did, however, induce a small increase in p-jun compared to non-treated controls; however, this was much less than that observed in the neurosphere cells or what was observed in response to curcumin (Figure 7c).

### 2.8. Bis-Chalcone 4j Demonstrates Reduced Toxicity to Non-Cancer Stem Cells

To determine the cytotoxicity of bis-chalcone 4j in a non-cancer stem cell line, we treated human mesenchymal stem cells (MSCs) with increasing concentrations of 4j (0.1–10 μM) and examined viability at 72 h as previously described. Compared to GSCs, the cytotoxicity induced by treatment was substantially lower, with an estimated IC50 of 13.1 μM (Supplemental Figure S4), suggesting a selective targeted effect of 4j towards GSCs.

## 3. Discussion

Despite an aggressive treatment regimen, the prognosis for GBM patients remains dismal. This poor outcome has in part been attributed to the presence of a small number of treatment resistant cells that are responsible for tumor recurrence and patient relapse. Cancer stem cells and cancer cells, in general, have an innate ability to adapt to extracellular stress (hypoxia, acidosis, nutrient and oxygen deprivation, etc.) and intracellular stress (ROS, oncogenic signaling pathways, etc.). Cellular stresses can result in a loss of protein homeostasis in the ER, prompting the activation of the UPR that consists of three different parallel signaling pathways initiated by ER resident transmembrane proteins, PERK, IRE1) and ATF6. Activation of these pathways aims to restore ER protein homeostasis by attenuating protein synthesis, upregulating the expression of specific protein chaperones to aid in protein folding and to stimulate ER associated protein degradation. However, if protein homeostasis cannot be restored, the UPR initiates apoptosis via the activation of the transcription factor CHOP. CHOP regulates the expression of both pro-survival and pro-cell death BCL-2 family members [33]. In addition, IRE1 mediated JNK activation has been shown to play an important role in ER stress mediated cell death by promoting mitochondrial-mediated cell death [34,35]. Lastly, ER localized Caspase 12 has been shown to mediate ER stress cell death as caspase-12-deficient mice were resistant to ER stress-induced apoptosis [36].

Of the 19 bis-chalcones synthesized and tested, only four (4c, 4g, 4j and 4n) significantly reduced GSC viability across a panel of six genetically distinct GBM patient-derived cancer stem cell lines with IC50s in the very low micromolar range (2.3–5.8 μM), considerably less than the previously reported IC50 for curcumin of approximately 25 Μm [15]. Our results indicate that a methoxy group (−OCH_3_) at the 3 position of the phenylic side appendages is important for inducing GSC death. In fact, at 10 μM, bis-chalcones 4c, 4e, 4g, 4h, 4j, 4m, 4n, and 4r reduced GSC viability to below 50% of non-treated controls in at least one cell line. Similarly, the methoxy group on the curcumin scaffold has been shown to be important for its biological activities [37,38]. Although bis-chalcone 4o possesses a methoxy group at the 3 position, it appears that its activity is compromised by the lipophilic benzyloxy moiety at position 4. Furthermore, it has been shown that the introduction of additional methoxy groups on the aromatic rings enhanced the anticancer effect on multiple cancer cell lines [39]. In analogy, we found that bis-chalcones containing multiple methoxy groups on the phenyl rings could be very cytotoxic. The most effective bis-chalcone was the 3,4,5 trimethoxy substituted analog (4j); however, the 2,4,5 trimethoxy substituted analog (4s), lacking substitution at position 3 was ineffective.

Bis-chalcone 4j induced robust cell death in all GSC lines with IC50 ranging from 2.3 μM (Glio4) to 2.7 μM (Glio11). Furthermore, 4j significantly reduced neurosphere formation at concentrations as low as 100 nM and virtually eliminating neurosphere formation at 250 nM suggesting modulation of GSC self-renewal properties. Pathway analysis of expression profiles generated using L1000 assay indicated that 4j treatment induced a stress response consistent with ER stress/UPR in the GSCs that grow as neurospheres but not in the GSC that grows adherently. Furthermore, drug connectivity analysis using the Clue compound database indicated that the signatures were highly connected to HSP inhibitors for the neurosphere cell lines, which is consistent with the pathway analysis, as HSP inhibition is associated with ER stress and UPR [40]. Consistent with these findings, Western blot analysis demonstrated that 4j induced robust induction of CHOP, p-jun (indicative of JNK activity) and caspase 12.

A putative mechanism by which bis-chalcone 4j is inducing ER stress in the neurospheres cells may be by directly increasing the number of misfolded proteins in the ER. Like curcumin, 4j is electrophilic. In fact, the presence of the pyridine ring between the two carbonyls makes the dienones more electrophilic, i.e., more reactive. Electrophiles (electron deficient) target electron rich nucleophiles such as side chains of the amino acids cysteine, histidine and lysine. One of the major modifications occurring in the ER is the formation of disulfide bonds, which stabilizes the newly formed protein [41]. It is possible that the reactive bis-chalcone is inducing ER stress by disrupting the formation of disulfide bonds between the thiol groups of the cysteine residues by forming a carbon-sulfur bond (Michael adduct formation). This disruption leads to an accumulation of unfolded proteins and, thus, ER stress similar to that of arylating quinones [42]. Additionally, it has been shown that GBM cells display a higher antioxidant capacity compared to normal cells, particularly higher level of Glutathione Reductase (GSR) and Glutathione (GSH) were detected [43]. Such elevated levels have been linked to the well-known GBM resistance to standard treatment with Temozolomide. It is then conceivable that the subtle chemical reactivity of our bis-chalcones could be targeting these antioxidants species (GSR, GSH), which would, in turn, lead to an increase in reactive oxygen species and, ultimately, ER stress [44].

However, the induction of ER stress/UPR was observed only in the neurosphere cell lines and not the adherent cell line, Glio9. Drug connectivity analysis indicated that 4j might induce cell death in Glio9 similar to that of estrogen inhibition. One potential connection of the UPR to estrogen signaling is the IRE1/XBP1 signaling pathway. Activated IRE1 cleaves XBP1 mRNA leading to the translation of a highly active transcription factor. The estrogen receptor antagonist, fulvestrant, was demonstrated to downregulate the IRE1/XBP1 signaling pathway in prolactinoma cells [45]. Conversely, Minchenko et al. recently demonstrated that IRE1 inhibition modulates the expression of genes encoding estrogen related proteins in glioma cells [46]. Recent data supports a role for IRE1/XBP1 in glioblastoma development and progression and as such a promising therapeutic target [47,48]. Glio9 was derived from a recurrent tumor, previously treated with both chemotherapy and radiation; therefore, it is not inconceivable that these cells would respond differently compared to cells generated from naive tumors. Regardless, our bis-chalcones, in particular 4j, induced robust cell death in all GSC lines examined. Moreover, our analogs obey Lipinski’s rule of 5, an important “benchmark test” in drug development, suggesting the *likely* oral bioavailability of such compounds [49]. Our encouraging results support 4j as a potential therapeutic lead for the development of a novel drug for the treatment of this deadly disease.

## 4. Materials and Methods

### 4.1. Chemistry

All reagents were obtained from Sigma-Aldrich (St. Louis, MO, USA) and were used directly without further purification. ^1^H- and ^13^C-NMR spectra were recorded at 500 and 125 MHz on Bruker. The spectra were referenced to the residual protonated solvents. Abbreviations such as *s*, *d*, *t*, *m*, *br*, and *dd* used in the description denote *singlet*, *doublet*, *triplet*, *multiplet*, *broad*, and *double doublet*, respectively. The chemical shifts and coupling constants were reported in parts per million (ppm) and hertz (Hz), respectively. High-resolution mass spectra were obtained on Bruker micrOTO-Q II mass spectrometer (Bruker, Billerica, MA, USA). The NMR and mass spectrometry data for the synthesized compounds are provided in the supplemental document. All intermediate and final products were monitored by thin layer chromatography (TLC) on 250 μm silica plates. Where applicable, the compounds were recrystallized from the proper solvent or purified by flash column chromatography on silica gel (200–300 mesh) with ethyl acetate/hexanes (1:1) as eluant.

The synthesis of bis-chalcones 4a–4s is outlined in Figure 1. The bis-chalcones were prepared by the Claisen–Schmidt condensation between 2,6-diacetylpyridine (1 equivalent) and the appropriate aryl aldehyde (2.1 equivalents) using either method a or b as shown. The reaction monitored by TLC. Upon completion, the reaction mixture was diluted with water and the solid formed was collected by vacuum filtration. The bis-chalcone was either purified by flash column chromatography or recrystallization. All the compounds were characterized by NMR (Nuclear Magnetic Resonance) Analysis and EI-HRMS (Electrospray Ionization-High Resolution Mass Spectrometry, Bruker, Billerica, MA, USA) Analysis. MOM-protected derivatives of 4-hydroxybenzaldehyde and vanillin were synthesized according to literature procedures [50]. More detailed description of the synthesis for each compound can be found in the Supplemental Materials.

### 4.2. Cell Culture

GSC lines Glio3, Glio4, Glio9, Glio11 and Glio14 have been previously described [15]. With Institutional Review Board (IRB) approval (number 20060858), the Glio38 cell line was derived from a patient’s resected tumor after receiving written consent. Briefly, tumor samples were physically and enzymatically digested and single cells were plated in DMEM/F12 3:1 supplemented with 20 ng/mL each of epidermal growth factor (EGF) and fibroblast growth factor (FGF), 2% Gem21 and 1% Penicillin/Streptomycin (P/S) to promote the growth of glioblastoma stem-like cells. Similar to the other neurosphere cell lines Glio3, Glio4, Glio11 and Glio14, Glio38 cells grew as neurospheres and expressed the putative GBM stem cell markers; cell surface proteins CD133 and A2B5, intermediate filament Nestin, RNA binding protein Musashi, oncogene BMi-1 and the transcriptional regulator Sox2 (Supplemental Figure S5). Glio9 was derived from a recurrent, post therapy, tumor and grows adherently and fails to express Sox2. [15] Human MSCs were obtained from Thermo Fisher Scientific (Waltham, MA, USA) and maintained in MEM supplemented with 20% fetal bovine serum (FBS) and 1% P/S. Our cell lines were routinely tested for mycoplasma using LookOut mycoplasma PCR detection kit (Sigma Aldrich, St. Louis, MO, USA) according to the manufacturer’s instructions and maintained at 37 °C in a humidified 5% CO_2_ incubator.

### 4.3. Drug Treatment

The bis-chalcones were dissolved in dimethyl sulfoxide (DMSO) at a concentration of 10 mM, vortexed and subsequently diluted 1:10 to obtain 1 mM and 0.1 mM stock concentrations. Viability was determined using the CellTiter 96® Aqueous One Solution Cell Proliferation Assay (MTS) assay (Promega Madison, WI, USA) as previously described [15]. Briefly, GSCs were seeded into 96-well plates using a modified neurosphere media containing 5% FBS at a density of 5000–10,000 cells per well, depending on the cell line. Cells were treated with increasing concentrations (0.1–10 μM) of each curcumin analog for 72 h. Media was aspirated and 100 μL of a 1:5 solution of MTS to cell culture media was added to each well and incubated for 1–4 h. Optical density was measured at 490 nm using a BoiTek Synergy HT plate reader (Biotek, Winooski, VT, USA). Viability of drug treated cells is expressed as the percent viable cells relative to non-treated cells (100% viability). Experiments were done in triplicate.

### 4.4. Neurosphere Forming Assay

To determine the effect of the curcumin analogs on stem cell activity, neurosphere assays were performed as previously described [15]. Briefly, single cells were seeded at 50–100 cells per well in a 96-well plate and treated with 100 nM, 250 nM or 500 nM bis-chalcones on day 0. Spheres greater than 50 microns were manually counted under microscopy on day 14. All experiments were done in triplicate.

### 4.5. RNA Analysis

To determine the molecular mechanism of bis-chalcone induced cell death, we exposed Glio11, Glio14 and Glio38 as neurospheres as well as the adherent Glio9 to 2.5 μM of bis-chalcones 4j, 4n or 2.5 μM curcumin (for comparison) and RNA was extracted 24 h later. Neurospheres were collected, spun down, washed with sterile PBS and RNA harvested using RNeasy Mini kit (Qiagen, Valencia, CA, USA) as per the manufacturer’s instructions. For the adherent culture, Glio9, cells were collected using accutase (Gemini), spun down, washed and RNA isolated as described above. RNA concentration was determined using Nanodrop 2000 spectrophotometer (Thermo Scientific, Waltham, MA, USA) and subsequently aliquotted for gene expression profiling. For Glio9, Glio14 and Glio38, three biological replicates each with four technical replicates were analyzed. For Glio11, two biological replicates each with six technical replicates were analyzed. Transcriptional profiles were generated by the LINCS Project, which utilizes a novel gene expression profiling method that measures the expression of 978 representative landmark transcripts [30].

#### 4.5.1. Transcriptional Impact

Transcriptional Consensus Signatures (TCSs) for each compound were calculated as described previously [24] using the Level 4 population-normalized L1000 data. Briefly, the TCSs quantify the genes that are consistently over/under expressed in multiple cell lines after a compound treatment.

#### 4.5.2. Functional Enrichment Analysis

Functional enrichment analysis was performed using the Functional Annotation Chart tool in David [25]. The median gene expression was calculated between biological replicates of the Level 5 L1000 data and genes with a |z-score| >= 1 were used as input in DAVID.

#### 4.5.3. CLUE Analysis

Connectivity Scores between the CLUE Touchstone (Reference) perturbagens were calculated using https://clue.io/. Hierarchical clustering (complete linkage, Euclidean distance) was performed on the 50 highest connected compounds for each condition (cell line treated with drug).

### 4.6. Western Blot Assay

Our protocol for Western blot assays has been described previously [51]. Cells were treated with 5 μM bis-chalcones or curcumin for 8 or 24 h cells are lysed with a RIPA buffer (1% sodium deoxycholate, 0.1% Sodium dodecyl sulfate (SDS), 1% Triton X-100, 10 mM Tris pH 8 and 140 mM NaCl) supplemented with 250 units per ml Benzonase, 1 mM dithiothreitol and phosSTOP phosphatase inhibitor cocktail and a cOmplete protease inhibitor cocktail (both from Roche, Indianapolis, IN, USA). Protein concentration determined using a bicinchoninic acid (BCA) protein assay (Thermo Scientific, Waltham, MA, USA), and 20 μg of protein was loaded onto 8, 12 or 15% polyacrylamide gels (BioRad Hercules, CA, USA) gels for electrophoresis and subsequently transferred onto nitrocellulose membranes. Membranes are incubated overnight with primary antibodies, washed and incubated with HRP conjugated secondary antibodies for 1 hour. Bands were visualized using Super-Signal™ West Pico Chemiluminescent Substrate (Thermo Scientific Waltham, MA, USA). Anti-C/EBP homologous protein (CHOP), anti-78 kDa glucose-regulated protein (GRP78), anti-caspase 12, anti-phospho c-jun (Ser63), anti-c-jun, anti-signal transducer and activator of transcription 3 (STAT3), anti-phospho-STAT3 (Tyr705), anti-poly ADP ribose polymerase (PARP) and anti-cleaved caspase 3 were all obtained from Cell Signaling Technology (Danvers, MA, USA). Anti-α-tubulin was obtained from Abcam (Eugene, OR, USA).

### 4.7. Statistical Analysis

Significance was determined using Student’s *t*-tests for all pairwise comparisons of the different treatments that were tested. The results are presented as the mean ± standard error mean (SEM). Significance was set at *p*  <  0.05.

## 5. Conclusions

Despite advances in neuroimaging and neurosurgical techniques, and an abundance of research aimed at understanding and targeting cell-signaling pathways driving GBM pathogenesis, GBM remains one of the most lethal brain tumors. The UPR is an adaptive mechanism initiated to mitigate ER stress resulting from the tumor microenvironment, oncogene activation, rapid cell proliferation as well as anti-cancer therapies. Specifically, the UPR plays a role in temozolomide and radiotherapy resistance in GBM [52,53]. However, if protein homeostasis cannot be restored, the UPR induces cell death. Here, we discovered a novel bis-chalcone (4j) capable of “weaponizing” the UPR to promote GBM stem cell death. Targeting the UPR is a novel strategy for treating this deadly disease and 4j is a promising lead compound for drug development.

## Supplementary Materials

The following are available online at https://www.mdpi.com/2072-6694/11/3/357/s1, Figure S1: Bis-chalcone 4r promotes morphological changes consistent with cell differentiation. Glio3 cells were treated with 10 μM bis-chalcone 4r for 3 days and examined by light microscopy. Arrows indicate neurospheres in non-treated cells, Figure S2: Bis-chalcone 4m induced cell death in 3 additional GSC lines. (a) Percent viability of bis-chalcone 4m treated Glio4, Glio11, and Glio14 cell lines. * *p* < 0.05 compared to non-treated controls; (b) IC50 of 4m for each cell line tested, Figure S3: Bis-chalcones do not substantially reduce STAT3 activity. Glio3 and Glio38 were treated with 5 μM of 4c, 4j and 4n and harvested 8 h later. Levels of p-STAT3, STAT3, cleaved caspase 3 were examined by Western blot analysis, Figure S4: Bis-chalcone 4j is less toxic to human MSCs. MSCs were treated with 0.1 μM–μ10 M 4j and percent viability determined at 72 h by MTS assay. * *p* < 0.05, compared to non-treated controls, Figure S5: Glio3 Characterization. Bright field images indicate that Glio3 grows as neurospheres in defined media. Expression of putative stem cell markers A2B5, CD133, Nestin, Bmi1, Musashi and Sox2 were evaluated by immunocytochemistry, Table S1: Transcriptional Consensus Signature for each compound, bis-chalcone 4j, 4n and curcumin. Table S1, the functional enrichment analysis for bis-chalcone 4n and curcumin and the Drug connectivity analysis for bis-chalcone 4n and curcumin can be found in the expression profiling data of the supplementary materials. Detailed description of bis-chalcone synthesis along with the spectral data for each compound can be found in the experimental section of the supplemental materials.

## References

[1] Ostrom Q.T., Gittleman H., Truitt G., Boscia A., Kruchko C., Barnholtz-Sloan J.S. (2018). CBTRUS Statistical Report: Primary Brain and Other Central Nervous System Tumors Diagnosed in the United States in 2011–2015. Neuro-Oncology.

[2] Stupp R., Mason W.P., van den Bent M.J., Weller M., Fisher B., Taphoorn M.J., Belanger K., Brandes A.A., Marosi C., Bogdahn U. (2005). Radiotherapy plus concomitant and adjuvant temozolomide for glioblastoma. N. Engl. J. Med..

[3] Chen J., Li Y., Yu T.S., McKay R.M., Burns D.K., Kernie S.G., Parada L.F. (2012). A restricted cell population propagates glioblastoma growth after chemotherapy. Nature.

[4] Kalkan R. (2015). Glioblastoma Stem Cells as a New Therapeutic Target for Glioblastoma. Clin. Med. Insights Oncol..

[5] Madden E., Logue S.E., Healy S.J., Manie S., Samali A. (2019). The role of the unfolded protein response in cancer progression: From oncogenesis to chemoresistance. Biol. Cell.

[6] Ciavattini A., Delli Carpini G., Serri M., Tozzi A., Leoni F., Di Loreto E., Saccucci F. (2018). Unfolded protein response, a link between endometrioid ovarian carcinoma and endometriosis: A pilot study. Oncol. Lett..

[7] Obacz J., Avril T., Le Reste P.J., Urra H., Quillien V., Hetz C., Chevet E. (2017). Endoplasmic reticulum proteostasis in glioblastoma-From molecular mechanisms to therapeutic perspectives. Sci. Signal..

[8] Obacz J., Avril T., Rubio-Patino C., Bossowski J.P., Igbaria A., Ricci J.E., Chevet E. (2017). Regulation of tumor-stroma interactions by the unfolded protein response. FEBS J..

[9] Penaranda Fajardo N.M., Meijer C., Kruyt F.A. (2016). The endoplasmic reticulum stress/unfolded protein response in gliomagenesis, tumor progression and as a therapeutic target in glioblastoma. Biochem. Pharmacol..

[10] Mann J. (2002). Natural products in cancer chemotherapy: Past, present and future. Nat. Rev. Cancer.

[11] Rodriguez G.A., Shah A.H., Gersey Z.C., Shah S.S., Bregy A., Komotar R.J., Graham R.M. (2016). Investigating the therapeutic role and molecular biology of curcumin as a treatment for glioblastoma. Ther. Adv. Med. Oncol..

[12] Wang Y., Yu J., Cui R., Lin J., Ding X. (2016). Curcumin in Treating Breast Cancer: A Review. J. Lab. Autom..

[13] Mehta H.J., Patel V., Sadikot R.T. (2014). Curcumin and lung cancer—A review. Targeted Oncol..

[14] Li Y., Zhang T. (2014). Targeting cancer stem cells by curcumin and clinical applications. Cancer Lett..

[15] Gersey Z.C., Rodriguez G.A., Barbarite E., Sanchez A., Walters W.M., Ohaeto K.C., Komotar R.J., Graham R.M. (2017). Curcumin decreases malignant characteristics of glioblastoma stem cells via induction of reactive oxygen species. BMC Cancer.

[16] Cheng A.L., Hsu C.H., Lin J.K., Hsu M.M., Ho Y.F., Shen T.S., Ko J.Y., Lin J.T., Lin B.R., Ming-Shiang W. (2001). Phase I clinical trial of curcumin, a chemopreventive agent, in patients with high-risk or pre-malignant lesions. Anticancer Res..

[17] Ramalingam P., Ko Y.T. (2015). Enhanced oral delivery of curcumin from N-trimethyl chitosan surface-modified solid lipid nanoparticles: Pharmacokinetic and brain distribution evaluations. Pharm. Res..

[18] Zhongfa L., Chiu M., Wang J., Chen W., Yen W., Fan-Havard P., Yee L.D., Chan K.K. (2012). Enhancement of curcumin oral absorption and pharmacokinetics of curcuminoids and curcumin metabolites in mice. Cancer Chemother. Pharmacol..

[19] Sasaki H., Sunagawa Y., Takahashi K., Imaizumi A., Fukuda H., Hashimoto T., Wada H., Katanasaka Y., Kakeya H., Fujita M. (2011). Innovative preparation of curcumin for improved oral bioavailability. Biol. Pharm. Bull..

[20] Chen P.H., Chang C.K., Shih C.M., Cheng C.H., Lin C.W., Lee C.C., Liu A.J., Ho K.H., Chen K.C. (2016). The miR-204-3p-targeted IGFBP2 pathway is involved in xanthohumol-induced glioma cell apoptotic death. Neuropharmacology.

[21] Champelovier P., Chauchet X., Hazane-Puch F., Vergnaud S., Garrel C., Laporte F., Boutonnat J., Boumendjel A. (2013). Cellular and molecular mechanisms activating the cell death processes by chalcones: Critical structural effects. Toxicol. In Vitro Int. J. Publ. Assoc. BIBRA.

[22] Robinson M.W., Overmeyer J.H., Young A.M., Erhardt P.W., Maltese W.A. (2012). Synthesis and evaluation of indole-based chalcones as inducers of methuosis, a novel type of nonapoptotic cell death. J. Med. Chem..

[23] Winter E., Devantier Neuenfeldt P., Chiaradia-Delatorre L.D., Gauthier C., Yunes R.A., Nunes R.J., Creczynski-Pasa T.B., Di Pietro A. (2014). Symmetric bis-chalcones as a new type of breast cancer resistance protein inhibitors with a mechanism different from that of chromones. J. Med. Chem..

[24] Lee J., Kotliarova S., Kotliarov Y., Li A., Su Q., Donin N.M., Pastorino S., Purow B.W., Christopher N., Zhang W. (2006). Tumor stem cells derived from glioblastomas cultured in bFGF and EGF more closely mirror the phenotype and genotype of primary tumors than do serum-cultured cell lines. Cancer Cell.

[25] Al-Omran F., Al-Awadi N., Edun M. (1994). Corrigendum-Synthesis of New 2-Pyrazoline Derivatives from 2, 6-Dicinnamoylpyridine and 1, 3-Dicinnamoylbenzene. J. Chem. Res.-Part S Synop..

[26] Reddy D.B., Seshamma T., Seenaiah B., Reddy M.R. (1991). Synthesis and Biological Activity of Some New Bis (2-pyrazolin-3-yl) benzenes and-pyridines. Indian J. Chem..

[27] Constable E.C., Figgemeier E., Hougen I.A., Housecroft C.E., Neuburger M., Schaffner S., Whall L.A. (2005). Hairpin helicates: A missing link between double-helicates and trefoil knots. Dalton Trans..

[28] Tan Y., Zhang Q., Yu J., Zhao X., Tian Y., Cui Y., Hao X., Yang Y., Qian G. (2013). Solvent effect on two-photon absorption (TPA) of three novel dyes with large TPA cross-section and red emission. Dyes Pigments.

[29] Weissenberger J., Priester M., Bernreuther C., Rakel S., Glatzel M., Seifert V., Kogel D. (2010). Dietary curcumin attenuates glioma growth in a syngeneic mouse model by inhibition of the JAK1,2/STAT3 signaling pathway. Clin. Cancer Res. Off. J. Am. Assoc. Cancer Res..

[30] Subramanian A., Narayan R., Corsello S.M., Peck D.D., Natoli T.E., Lu X., Gould J., Davis J.F., Tubelli A.A., Asiedu J.K. (2017). A Next Generation Connectivity Map: L1000 Platform and the First 1,000,000 Profiles. Cell.

[31] Stathias V., Jermakowicz A.M., Maloof M.E., Forlin M., Walters W., Suter R.K., Durante M.A., Williams S.L., Harbour J.W., Volmar C.H. (2018). Drug and disease signature integration identifies synergistic combinations in glioblastoma. Nat. Commun..

[32] Huang da W., Sherman B.T., Lempicki R.A. (2009). Systematic and integrative analysis of large gene lists using DAVID bioinformatics resources. Nat. Protocols.

[33] Li Y., Guo Y., Tang J., Jiang J., Chen Z. (2014). New insights into the roles of CHOP-induced apoptosis in ER stress. Acta Biochim. Biophys. Sin..

[34] Joo H., Lee H.J., Shin E.A., Kim H., Seo K.H., Baek N.I., Kim B., Kim S.H. (2016). c-Jun N-terminal Kinase-Dependent Endoplasmic Reticulum Stress Pathway is Critically Involved in Arjunic Acid Induced Apoptosis in Non-Small Cell Lung Cancer Cells. Phytother. Res..

[35] Zheng Q.Y., Li P.P., Jin F.S., Yao C., Zhang G.H., Zang T., Ai X. (2013). Ursolic acid induces ER stress response to activate ASK1-JNK signaling and induce apoptosis in human bladder cancer T24 cells. Cell. Signal..

[36] Nakagawa T., Zhu H., Morishima N., Li E., Xu J., Yankner B.A., Yuan J. (2000). Caspase-12 mediates endoplasmic-reticulum-specific apoptosis and cytotoxicity by amyloid-beta. Nature.

[37] Yang H., Du Z., Wang W., Song M., Sanidad K., Sukamtoh E., Zheng J., Tian L., Xiao H., Liu Z. (2017). Structure-Activity Relationship of Curcumin: Role of the Methoxy Group in Anti-inflammatory and Anticolitis Effects of Curcumin. J. Agric. Food Chem..

[38] Indira Priyadarsini K. (2013). Chemical and structural features influencing the biological activity of curcumin. Curr. Pharm. Des..

[39] Zhou D., Ding N., Zhao S., Li D., Van Doren J., Qian Y., Wei X., Zheng X. (2014). Synthesis and evaluation of curcumin-related compounds containing inden-2-one for their effects on human cancer cells. Biol. Pharm. Bull..

[40] Bi K., Nishihara K., Machleidt T., Hermanson S., Wang J., Sakamuru S., Huang R., Xia M. (2015). Identification of known drugs targeting the endoplasmic reticulum stress response. Anal. Bioanal. Chem..

[41] Trivedi M.V., Laurence J.S., Siahaan T.J. (2009). The role of thiols and disulfides on protein stability. Curr. Protein Pept. Sci..

[42] Wang X., Thomas B., Sachdeva R., Arterburn L., Frye L., Hatcher P.G., Cornwell D.G., Ma J. (2006). Mechanism of arylating quinone toxicity involving Michael adduct formation and induction of endoplasmic reticulum stress. Proc. Natl. Acad. Sci. USA.

[43] Zhu Z., Du S., Du Y., Ren J., Ying G., Yan Z. (2018). Glutathione reductase mediates drug resistance in glioblastoma cells by regulating redox homeostasis. J. Neurochem..

[44] Jackson P.A., Widen J.C., Harki D.A., Brummond K.M. (2017). Covalent Modifiers: A Chemical Perspective on the Reactivity of alpha,beta-Unsaturated Carbonyls with Thiols via Hetero-Michael Addition Reactions. J. Med. Chem..

[45] Wang C., Bai M., Wang X., Tan C., Zhang D., Chang L., Li G., Xie L., Su J., Wen Y. (2018). Estrogen receptor antagonist fulvestrant inhibits proliferation and promotes apoptosis of prolactinoma cells by regulating the IRE1/XBP1 signaling pathway. Mol. Med. Rep..

[46] Minchenko D.O., Riabovol O.O., Ratushna O.O., Minchenko O.H. (2017). Hypoxic regulation of the expression of genes encoded estrogen related proteins in U87 glioma cells: Effect of IRE1 inhibition. Endocr. Regul..

[47] Lhomond S., Avril T., Dejeans N., Voutetakis K., Doultsinos D., McMahon M., Pineau R., Obacz J., Papadodima O., Jouan F. (2018). Dual IRE1 RNase functions dictate glioblastoma development. EMBO Mol. Med..

[48] Jabouille A., Delugin M., Pineau R., Dubrac A., Soulet F., Lhomond S., Pallares-Lupon N., Prats H., Bikfalvi A., Chevet E. (2015). Glioblastoma invasion and cooption depend on IRE1alpha endoribonuclease activity. Oncotarget.

[49] Lipinski C.A., Lombardo F., Dominy B.W., Feeney P.J. (2001). Experimental and computational approaches to estimate solubility and permeability in drug discovery and development settings. Adv. Drug Deliv. Rev..

[50] Wuts P.G., Greene T.W. (2006). Greene’s Protective Groups in Organic Synthesis.

[51] Graham R.M., Hernandez F., Puerta N., De Angulo G., Webster K.A., Vanni S. (2016). Resveratrol augments ER stress and the cytotoxic effects of glycolytic inhibition in neuroblastoma by downregulating Akt in a mechanism independent of SIRT1. Exp. Mol. Med..

[52] Hombach-Klonisch S., Mehrpour M., Shojaei S., Harlos C., Pitz M., Hamai A., Siemianowicz K., Likus W., Wiechec E., Toyota B.D. (2018). Glioblastoma and chemoresistance to alkylating agents: Involvement of apoptosis, autophagy, and unfolded protein response. Pharmacol. Ther..

[53] Dadey D.Y., Kapoor V., Khudanyan A., Urano F., Kim A.H., Thotala D., Hallahan D.E. (2016). The ATF6 pathway of the ER stress response contributes to enhanced viability in glioblastoma. Oncotarget.

