# Supplementary material for: Novel Curcumin Inspired Bis-Chalcone Promotes Endoplasmic Reticulum Stress and Glioblastoma Neurosphere Cell Death
Source: Cancers (Basel). 2019 Mar 13;11(3):357. doi: 10.3390/cancers11030357 (PMC6468769; doi:10.3390/cancers11030357)
Supplement: Supplementary file 1 [file cancers-11-00357-s001.zip › Graham Cancers 444757 supplemental files/cancers-444757-Graham supplemental figures -Proofreading.pdf]

Supplementary Materials: Novel *Curcumin Inspired* Bis-Chalcone Promotes Endoplasmic Reticulum Stress and Glioblastoma Neurosphere Cell Death

Lorenzo Sansalone, Eduardo A. Veliz, Nadia G. Myrthil, Vasileios Stathias, Winston Walters, Ingrid I. Torrens, Stephan C. Schürer, Steven Vanni, Roger M. Leblanc and Regina M. Graham

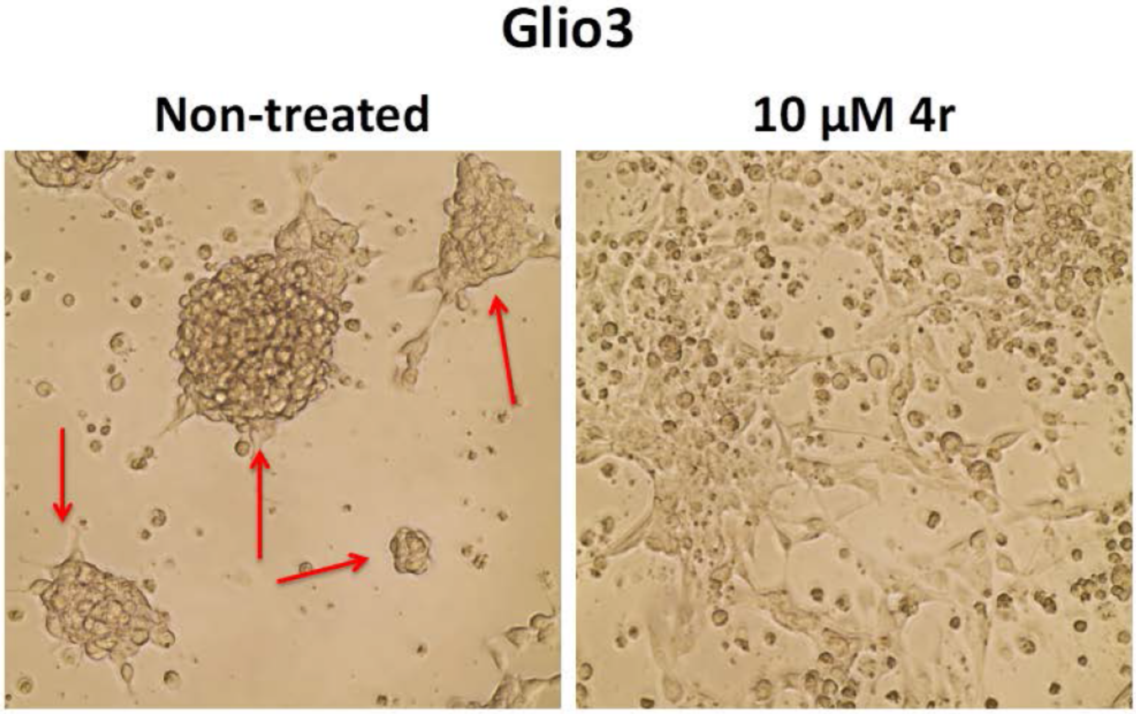

Figure S1. Bis-chalcone 4r promotes morphological changes consistent with cell differentiation. Glio3 cells were treated with 10 μM bis-chalcone 4r for 3 days and examined by light microscopy. Arrows indicate neurospheres in non-treated cells.

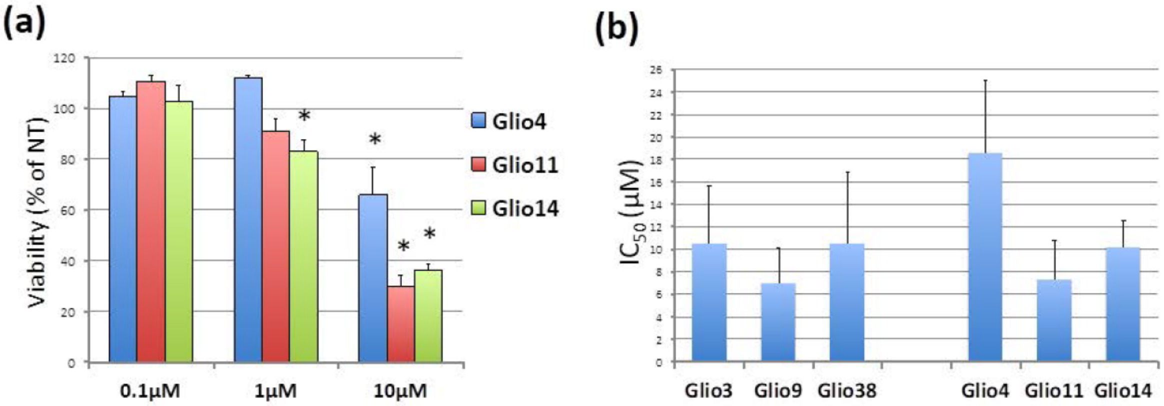

Figure S2. Bis-chalcone 4m induced cell death in 3 additional GSC lines. (**a**) Percent viability of bis-chalcone 4m treated Glio4, Glio11, and Glio14 cell lines. * $p < 0.05$ compared to non-treated controls. (**b**) IC50 of 4m for each cell line tested.

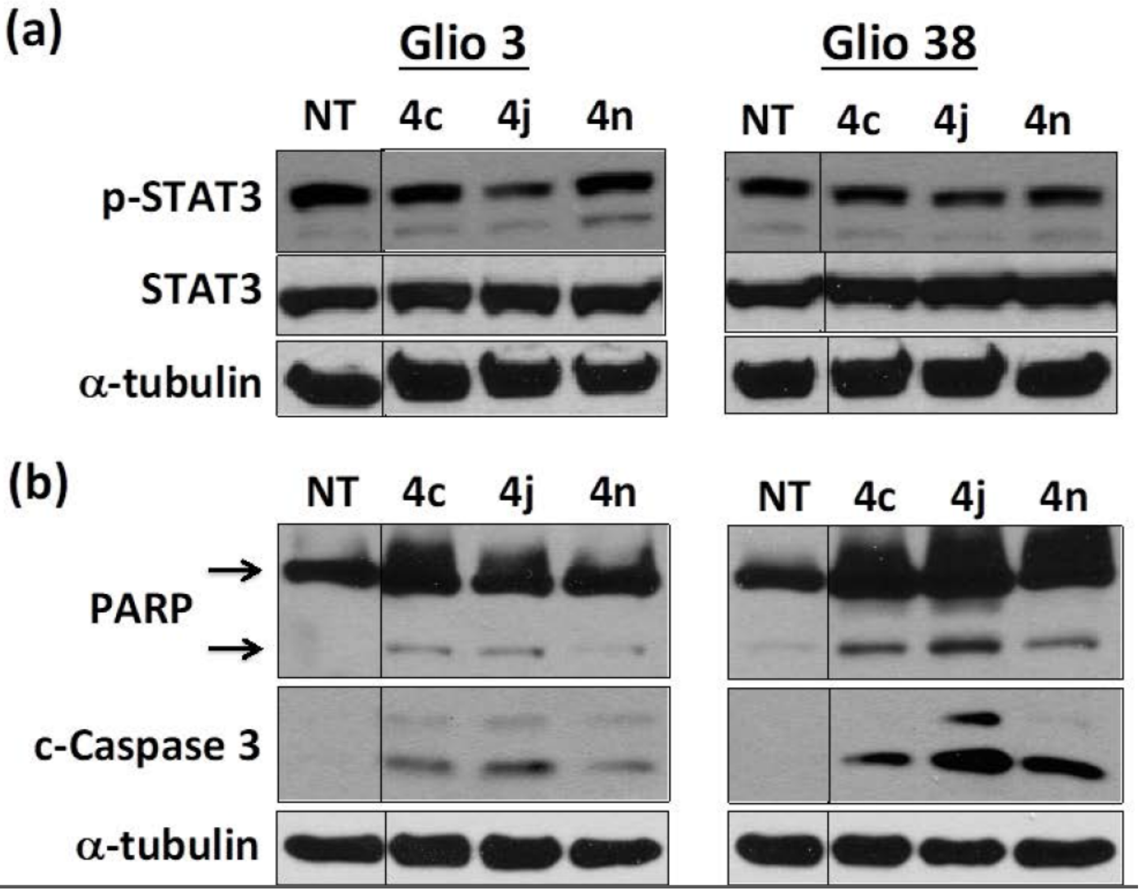

Figure S3. Bis-chalcones do not substantially reduce STAT3 activity. Glio3 and Glio38 were treated with 5 μM of 4c, 4j and 4n and harvested 8 hours later. Levels of p-STAT3, STAT3, cleaved caspase 3 were examined by western blot analysis.

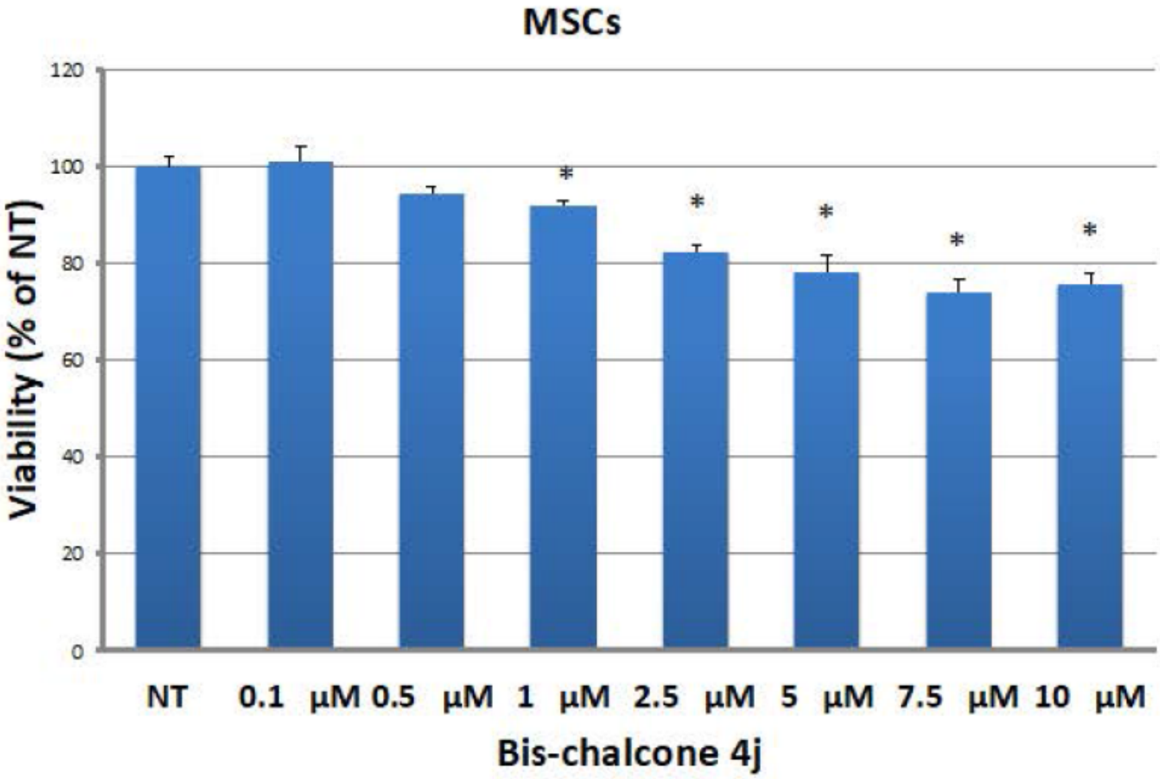

Figure S4. Bis-chalcone 4j is less toxic to human MSCs. MSCs were treated with 0.1 μM - μ10 M 4j and percent viability determined at 72 hours by MTS assay. * $p < 0.05$, compared to non-treated controls.

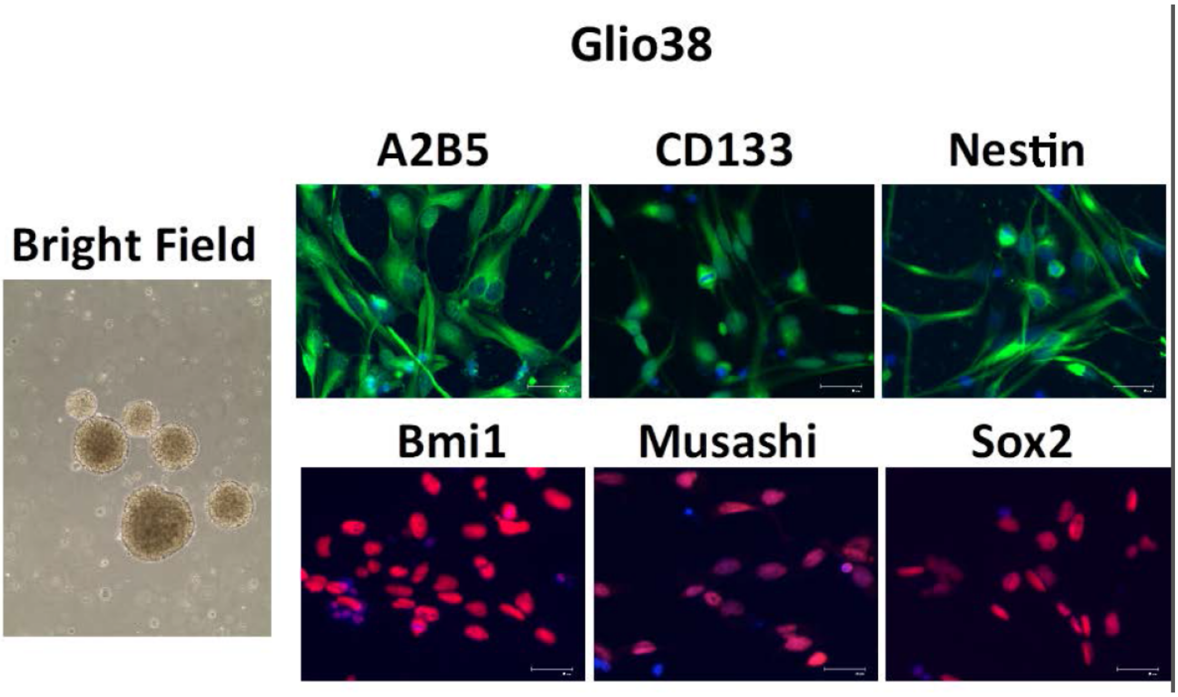

Figure S5. Glio3 Characterization. Bright field images indicate that Glio3 grows as neurospheres in defined media. Expression of putative stem cell markers A2B5, CD133, Nestin, Bmi1, Musashi and Sox2 were evaluated by immunocytochemistry.
