# Supplementary material for: Novel Curcumin Inspired Bis-Chalcone Promotes Endoplasmic Reticulum Stress and Glioblastoma Neurosphere Cell Death
Source: Cancers (Basel). 2019 Mar 13;11(3):357. doi: 10.3390/cancers11030357 (PMC6468769; doi:10.3390/cancers11030357)
Supplement: Supplementary file 1 [file cancers-11-00357-s001.zip › Graham Cancers 444757 supplemental files/Graham Appendix A expression profiling data.pdf]

4j

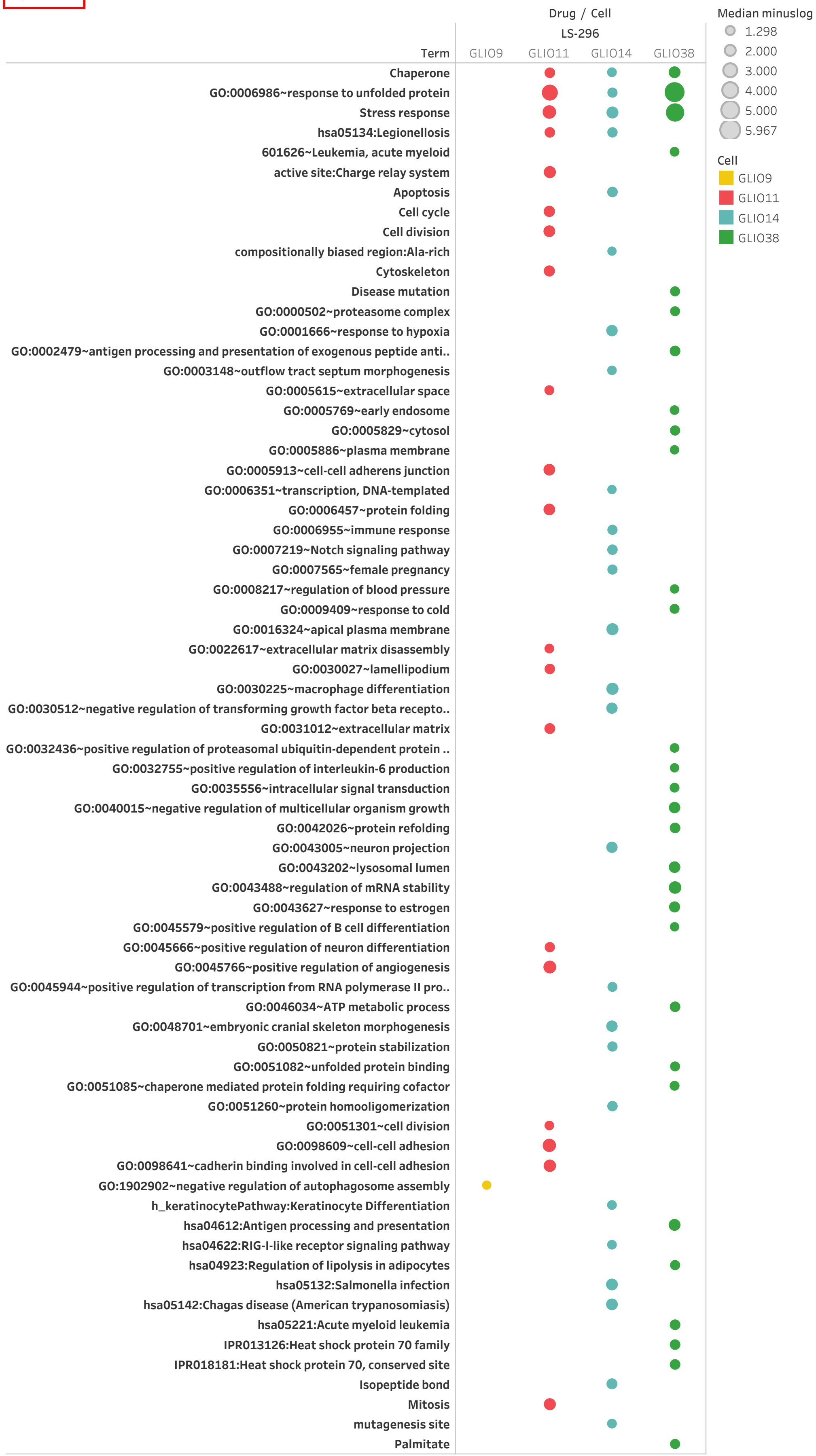

Cell (color) and median of minuslog (size) broken down by Drug and Cell vs. Term. The data is filtered on average of P Value, which ranges from 1.08e-06 to 0.0506. The view is filtered on Drug and Cell. The Drug filter keeps LS-296. The Cell filter keeps GLIO11, GLIO14, GLIO38 and GLIO9.

4n

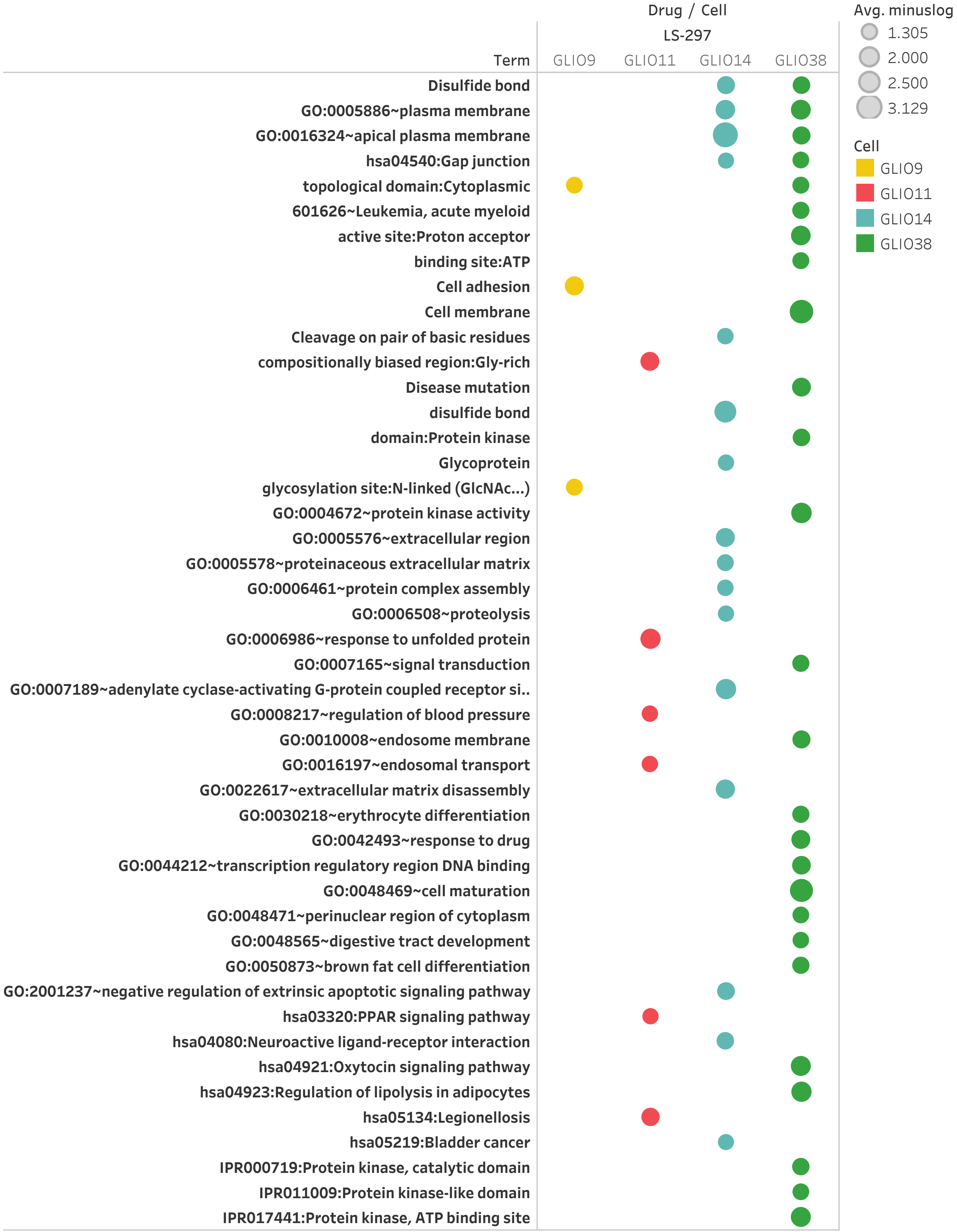

Cell (color) and average of minuslog (size) broken down by Drug and Cell vs. Term. The data is filtered on average of P Value, which ranges from 1.08e-06 to 0.05. The view is filtered on Term, Drug and Cell. The Term filter keeps 321 of 321 members. The Drug filter keeps LS-297. The Cell filter keeps GLIO11, GLIO14, GLIO38 and GLIO9.

4n

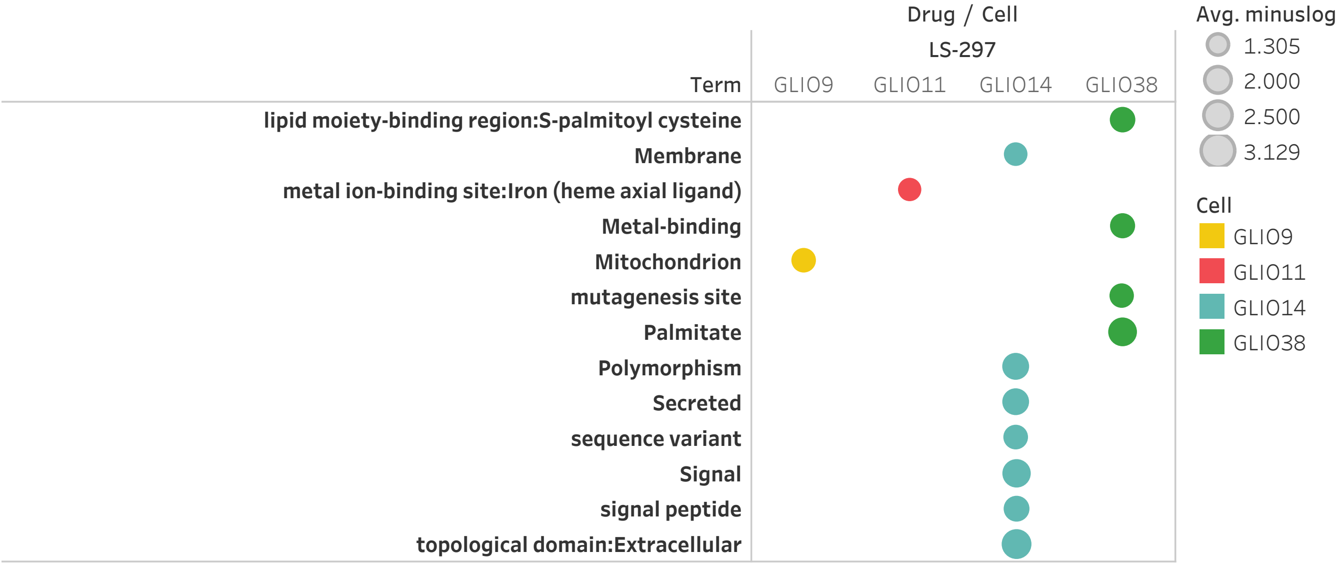

Cell (color) and average of minuslog (size) broken down by Drug and Cell vs. Term. The data is filtered on average of P Value, which ranges from 1.08e-06 to 0.05. The view is filtered on Term, Drug and Cell. The Term filter keeps 321 of 321 members. The Drug filter keeps LS-297. The Cell filter keeps GLIO11, GLIO14, GLIO38 and GLIO9.

Curcumin

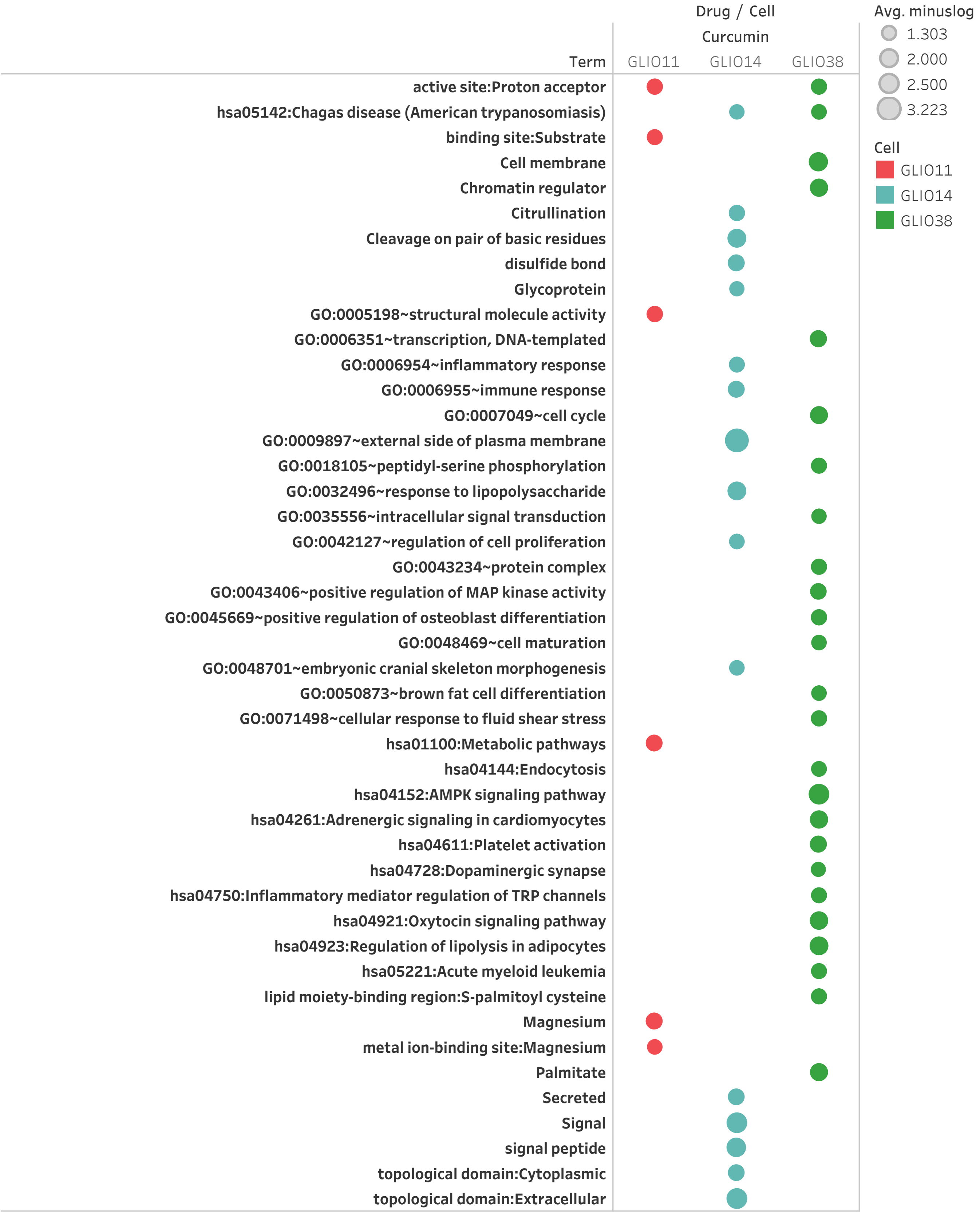

Cell (color) and average of minuslog (size) broken down by Drug and Cell vs. Term. The data is filtered on average of P Value, which ranges from 1.08e-06 to 0.0507. The view is filtered on Cell, Term and Drug. The Cell filter keeps GLIO11, GLIO14 and GLIO38. The Term filter keeps 321 of 321 members. The Drug filter keeps Curcumin.

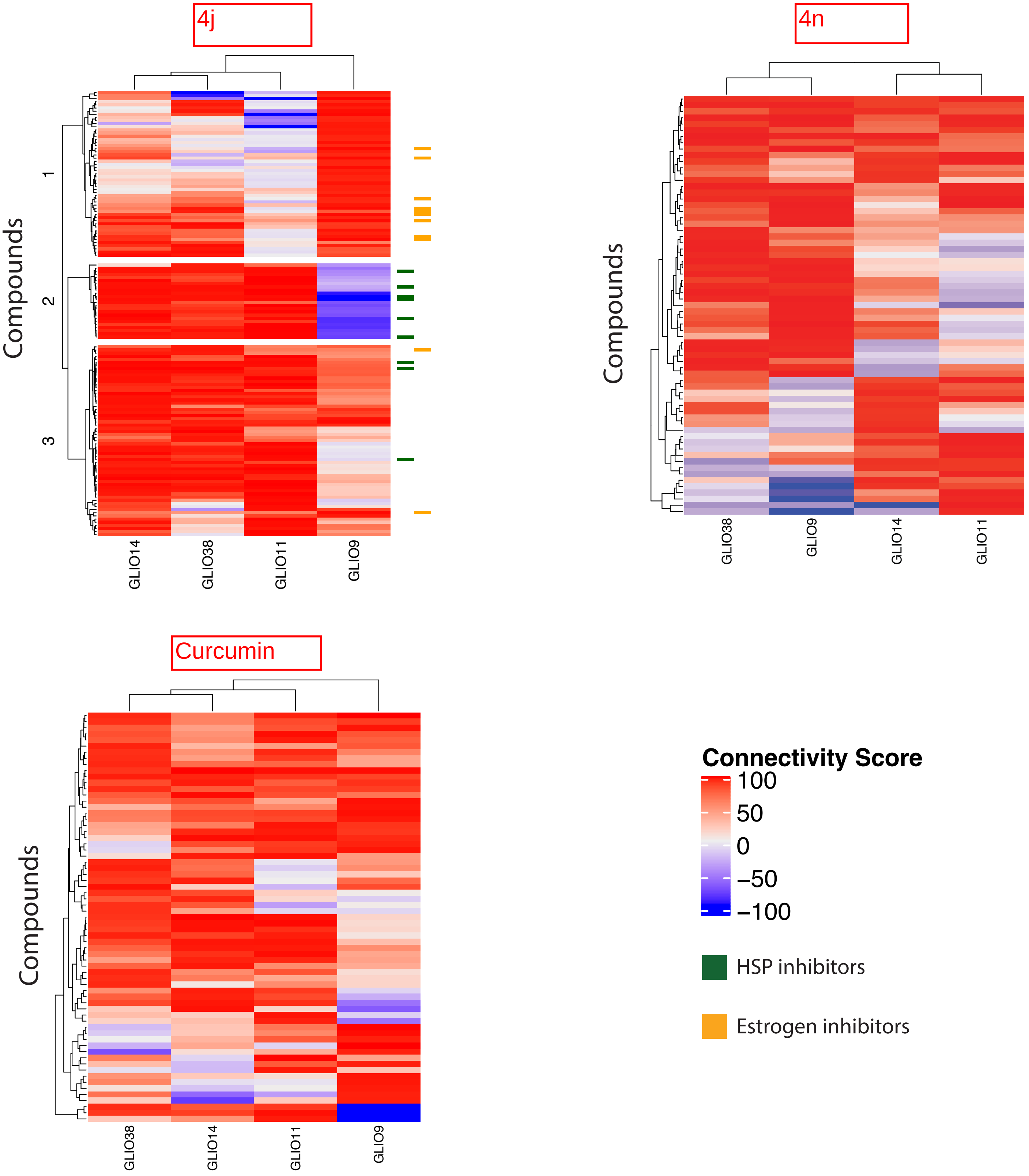

4j
Compounds
1
2
3
GLIO14
GLIO38
GLIO11
GLIO9
4n
Compounds
GLIO38
GLIO9
GLIO14
GLIO11
Curcumin
Compounds
GLIO38
GLIO14
GLIO11
GLIO9
Connectivity Score
100
50
0
−50
−100
HSP inhibitors
Estrogen inhibitors

Table 1: Up regulated and down regulated genes accross all GSCs exposed to bis-chalcone 4j (LS 296), 4n (LS 297) and curcumin.

Gene_Symbol	Gene_ID	CURCUMIN	LS−296	LS−297
PSME1	5720	0	0	0
CISD1	55847	0	2	0
VDAC1	7416	0	0	0
SORBS3	10174	0	0	0
SPDEF	25803	0	0	0
ATF1	466	0	0	0
SPAG4	6676	0	0	0
E2F2	1870	0	0	0
RHEB	6009	0	0	0
IGF1R	3480	0	0	2
SLC5A6	8884	0	0	0
FOXO3	2309	0	0	0
RGS2	5997	0	0	0
GSTM2	2946	0	0	0
SCYL3	57147	0	0	0
RHOA	387	0	−2	0
TBX2	6909	0	0	0
DNAJA3	9093	0	0	0
IL1B	3553	0	0	0
ASAH1	427	0	0	−2
DCUN1D4	23142	0	0	0
DYRK3	8444	0	0	0
RALA	5898	0	0	0
ARHGEF12	23365	0	0	0

POLG2	11232	0	0	0
SOX2	6657	0	0	0
TRAPPC3	27095	0	0	0
SERPINE1	5054	0	0	0
TCFL5	10732	−2	0	0
CRK	1398	0	0	0
HLA.DMA	#N/A	0	0	0
EGF	1950	0	3	0
RBM34	23029	2	0	0
ITGB1BP1	9270	2	0	0
SPTLC2	9517	0	0	0
ACLY	47	0	0	0
APP	351	0	0	0
RAD51C	5889	0	0	0
TSKU	25987	0	0	0
SMC1A	8243	0	0	2
TMEM2	23670	0	0	0
NOS3	4846	3	0	0
YME1L1	10730	0	0	0
ACAA1	30	0	0	0
CSNK1A1	1452	0	0	0
HSPA8	3312	0	0	0
NFATC4	4776	0	0	0
CCNB1	891	0	0	0
TBP	6908	0	0	0

SLC2A6	11182	0	0	0
WFS1	7466	0	0	0
EZH2	2146	0	0	−2
UBE3B	89910	0	0	0
ICAM3	3385	0	0	0
NPDC1	56654	0	0	0
ALDH7A1	501	0	0	0
BRCA1	672	0	0	0
NFATC3	4775	0	0	−2
PSMD4	5710	0	0	0
PCNA	5111	0	0	0
PHKG2	5261	0	0	0
ETV1	2115	0	0	0
POLR2I	5438	0	0	0
KDM5B	10765	0	0	0
PSMB8	5696	0	0	0
AKAP8L	26993	0	0	0
COG4	25839	0	0	0
MCM3	4172	0	0	0
PLEKHJ1	55111	0	0	0
HAT1	8520	0	0	0
TERT	7015	0	0	0
G3BP1	10146	0	0	0
SNX13	23161	0	0	0
PAK4	10298	0	0	−3

TPD52L2	7165	0	0	0
DPH2	1802	0	0	0
PIH1D1	55011	0	0	0
PRKCQ	5588	0	−2	0
SNX6	58533	0	0	0
EED	8726	0	−2	0
SHB	6461	0	0	2
UTP14A	10813	0	0	0
WIPF2	147179	−2	0	0
NENF	29937	0	−2	0
TSEN2	80746	0	0	0
RPN1	6184	0	0	0
SUV39H1	6839	0	0	0
C2CD2L	9854	−2	0	0
LYPLA1	10434	0	0	0
BIRC2	329	0	0	0
GABPB1	2553	0	0	0
MAP2K5	5607	0	0	0
POLR2K	5440	0	0	0
PTK2B	2185	0	0	0
PUF60	22827	0	0	0
DHRS7	51635	0	0	0
PAF1	54623	0	−2	0
ARID5B	84159	2	0	0
POLR1C	9533	−2	0	0

PAK1	5058	0	0	0
RUVBL1	8607	0	0	0
AKT1	207	0	0	0
CDC42	998	0	0	0
IGHMBP2	3508	0	0	0
SENP6	26054	0	0	0
ADO	84890	0	0	0
HSPB1	3315	0	3	0
EBP	10682	0	0	0
TCTN1	79600	0	0	0
AKR7A2	8574	0	0	0
CASK	8573	0	0	0
PPP2R5E	5529	0	0	0
RRP8	23378	0	0	0
LAMA3	3909	0	0	0
PIK3C3	5289	0	0	0
BHLHE40	8553	0	0	0
CCNE2	9134	0	0	0
BDH1	622	0	0	0
FEZ2	9637	0	−2	0
IKBKAP	8518	0	0	0
SH3BP5	9467	0	0	0
PPP2R5A	5525	0	0	0
SGCB	6443	0	0	0
EML3	256364	0	0	0

ENOPH1	58478	0	0	0	
BIRC5	332	0	0	0	
CNOT4	4850	0	0	0	
EPRS	2058	0	0	0	
SNAP25	6616	0	0	0	
JMJD6	23210	0	0	2	
PAX8	7849	0	0	0	
CBLB	868	0	0	0	
SLC35F2	54733	0	0	0	
ANO10	55129	0	0	0	
PDS5A	23244	0	0	0	
DFFB	1677	−2	0	0	
STUB1	10273	0	0	0	
PGAM1	5223	0	−2	0	
TGFB3	7043	0	0	0	
BAD	572	0	0	0	
XBP1	7494	0	0	0	
NMT1	4836	0	0	0	
PSMD10	5716	0	0	−2	
ARFIP2	23647	0	0	0	
RAB11FIP2		22841	0	0	0
FPGS	2356	0	0	0	
STAMBP	10617	0	0	0	
PRKX	5613	0	0	0	
RNH1	6050	0	0	0	

ATG3	64422	0	0	0
FAH	2184	0	0	0
NCK1	4690	0	0	0
PLP2	5355	0	0	0
CFLAR	8837	0	0	0
SOX4	6659	0	0	0
HEATR1	55127	0	0	0
TESK1	7016	0	0	0
HN1L	90861	0	0	0
WDR7	23335	0	0	0
AKAP8	10270	0	0	0
RRAGA	10670	0	0	0
ELOVL6	79071	0	0	0
SLC1A4	6509	0	0	0
ACBD3	64746	0	0	0
GRWD1	83743	0	0	0
PMAIP1	5366	0	0	0
STAT1	6772	0	0	0
FOXJ3	22887	0	0	0
TWF2	11344	0	0	0
HDAC6	10013	0	0	0
MTERFD1	51001	−2	0	0
CCND1	595	0	0	0
CHIC2	26511	0	0	0
PXN	5829	−2	0	0

USP7	7874	0	0	0
NOSIP	51070	0	0	0
PHKB	5257	0	0	2
DLD	1738	0	0	0
NFKBIB	4793	2	0	0
CASC3	22794	0	0	0
IDE	3416	0	0	0
CD40	958	0	0	0
MLEC	9761	0	−3	0
GLI2	2736	0	0	0
PRPF4	9128	0	0	0
MELK	9833	0	0	0
USP22	23326	0	0	0
DAXX	1616	0	2	0
TMED10	10972	0	0	0
CHERP	10523	0	0	0
SMAD3	4088	0	0	0
OXA1L	5018	0	0	0
PIK3CA	5290	0	0	0
SNX7	51375	0	0	0
TRAPPC6A	79090	0	−2	−2
ATP2C1	27032	0	0	−2
CDCA4	55038	0	0	0
TBC1D9B	23061	0	0	0
USP14	9097	0	−3	0

APPBP2	10513	0	0	0
CDC20	991	0	0	0
NARFL	64428	0	0	0
RPS6	6194	0	0	0
DDX42	11325	0	0	0
ECH1	1891	0	0	0
HDAC2	3066	0	0	0
IKBKE	9641	0	0	0
CCL2	6347	0	0	0
DFFA	1676	0	0	0
MRPL19	9801	0	0	0
WDTC1	23038	0	0	0
PLSCR3	57048	0	0	0
SUZ12	23512	0	0	0
CSNK2A2	1459	0	0	−2
H2AFV	94239	0	0	0
NUSAP1	51203	0	0	0
TICAM1	148022	0	0	0
FAM69A	388650	−2	0	0
KIAA0100	9703	0	0	0
PAICS	10606	0	0	2
SRC	6714	0	0	0
BMP4	652	0	0	0
CDK2	1017	2	0	0
EDN1	1906	0	0	0

XPO7	23039	0	0	0	
FAIM	55179	0	0	0	
KIAA0494	9813	0	0	0	
PXMP2	5827	2	0	0	
SQSTM1	8878	0	2	0	
HIF1A	3091	0	0	0	
TIMM17B	10245	0	0	0	
STXBP2	6813	0	0	0	
WRB	7485	0	0	0	
ETFB	2109	2	0	0	
MNAT1	4331	−2	0	0	
CAMSAP2	23271	−2	0	0	
HPRT1	3251	0	0	0	
ARNT2	9915	0	0	0	
RRS1	23212	0	0	0	
ADAT1	23536	0	0	0	
SPAG7	9552	0	0	0	
MAPK1IP1L		93487	0	0	0
SCAND1	51282	0	0	2	
MFSD10	10227	0	0	0	
MSH6	2956	0	0	0	
COPS7A	50813	0	0	0	
UBE2C	11065	0	0	0	
FIS1	51024	0	0	0	
HS2ST1	9653	0	0	0	

NUDT9	53343	0	0	0
TXLNA	200081	0	0	0
CSRP1	1465	0	0	0
DNAJB1	3337	−3	4	0
METRN	79006	0	0	0
TOPBP1	11073	0	0	2
ABCF1	23	0	0	0
TCTA	6988	0	0	0
LGMN	5641	0	0	0
TLK2	11011	0	0	0
KIAA1033	23325	0	0	0
PTGS2	5743	0	0	0
DNAJB6	10049	0	0	0
PLA2G4A	5321	0	0	0
MAT2A	4144	0	0	0
TLR4	7099	0	0	0
HMGCS1	3157	0	0	0
MBTPS1	8720	−2	−2	−2
SPRED2	200734	0	0	0
ZNF274	10782	0	0	0
CLIC4	25932	0	−3	0
MAP7	9053	0	0	0
CHAC1	79094	0	2	0
EIF4G1	1981	0	0	0
BTK	695	0	0	0

GNAS	2778	0	0	0
MYL9	10398	0	0	0
TEX10	54881	0	0	0
PSMD2	5708	0	0	0
ZNF586	54807	0	0	0
CLTC	1213	0	0	0
IKZF1	10320	0	0	0
APBB2	323	0	0	0
HSD17B10	3028	0	0	0
RNF167	26001	0	0	0
STX1A	6804	0	0	0
ALDOA	226	0	0	0
EPHA3	2042	0	0	0
STAT5B	6777	0	−2	0
TXNDC9	10190	−2	0	2
CCNA2	890	0	0	0
VGLL4	9686	0	0	0
CASP10	843	0	0	0
SDHB	6390	0	0	0
ETS1	2113	0	2	0
YTHDF1	54915	0	−2	0
FOXO4	4303	0	0	0
LSM5	23658	0	0	0
TOMM34	10953	0	0	2
ZMYM2	7750	0	0	0

CHMP6	79643	0	0	2
KIAA0907	22889	0	0	0
CASP7	840	0	2	0
HK1	3098	0	0	0
PAPD7	11044	0	0	0
PIGB	9488	0	0	0
CHEK2	11200	0	0	0
PLOD3	8985	0	−2	0
ACD	65057	0	0	0
TRAK2	66008	0	0	0
SUPV3L1	6832	0	0	0
VAPB	9217	−2	0	−2
GPATCH8	23131	0	0	0
HSPD1	3329	0	4	0
BUB1B	701	0	0	0
TGFBR2	7048	0	0	0
MRPL12	6182	0	0	0
NOTCH1	4851	0	0	0
PNP	4860	0	0	0
SHC1	6464	0	2	0
ARPP19	10776	0	0	0
CEBPA	1050	0	0	0
PSMF1	9491	0	0	0
RTN2	6253	0	0	0
NPEPL1	79716	0	0	0

USP1	7398	0	0	0
RPA1	6117	0	0	0
SIRT3	23410	0	0	0
KLHDC2	23588	0	0	0
UBQLN2	29978	0	0	0
DMTF1	9988	0	0	0
SCARB1	949	0	0	0
CLPX	10845	0	0	0
TPM1	7168	0	0	0
TM9SF2	9375	0	0	−2
TXNL4B	54957	0	0	0
ATF6	22926	0	0	0
PRR15L	79170	0	−2	0
NUP88	4927	0	0	0
ST6GALNAC2	10610	0	0	0
ADH5	128	0	0	3
FUT1	2523	0	0	0
GOLT1B	51026	0	0	0
PRUNE	58497	0	0	0
PPOX	5498	0	0	0
SYPL1	6856	0	0	0
CANT1	124583	0	0	0
ZNF451	26036	0	0	0
HOMER2	9455	0	0	0
RALB	5899	0	0	0

CLTB	1212	0	0	0	
TRIM13	10206	−2	0	−2	
FBXO7	25793	0	0	0	
PHKA1	5255	0	0	0	
CYCS	54205	0	−2	0	
PPARD	5467	0	0	2	
ENOSF1	55556	0	0	0	
MAP4K4	9448	0	0	0	
PAN2	9924	0	0	0	
STAT3	6774	0	0	0	
LIG1	3978	0	0	0	
PPIC	5480	0	0	0	
EIF5	1983	−2	0	0	
GNB5	10681	0	0	0	
GNAI1	2770	0	0	0	
INSIG1	3638	0	0	0	
DNMT3A	1788	0	0	0	
TM9SF3	56889	0	0	0	
CCDC86	79080	0	0	0	
HIST2H2BE		8349	0	0	0
S100A4	6275	0	0	0	
SLC25A14	9016	0	0	0	
KTN1	3895	0	0	0	
TBXA2R	6915	3	0	0	
ARHGAP1	392	0	0	0	

FBXO11	80204	0	0	0
HERPUD1	9709	0	0	0
LPAR2	9170	0	0	0
COASY	80347	0	0	0
RPL39L	116832	0	0	0
BPHL	670	0	0	0
RASA1	5921	0	0	0
ATMIN	23300	0	0	0
MAPKAPK3	7867	0	0	0
CASP3	836	0	0	0
MBOAT7	79143	0	0	0
PNKP	11284	0	2	0
PSME2	5721	0	0	0
NISCH	11188	0	0	0
SLC35A3	23443	0	0	0
CPSF4	10898	0	0	0
LSR	51599	0	0	0
COPB2	9276	0	0	0
MEF2C	4208	0	0	0
GADD45A	1647	0	0	0
KDM5A	5927	0	0	0
FAM63A	55793	0	0	0
SMNDC1	10285	0	0	0
NUDCD3	23386	0	0	0
RAB4A	5867	0	0	0

FGFR2	2263	0	0	0
IFRD2	7866	2	0	0
BACE2	25825	0	0	0
HMGCR	3156	0	−2	0
LYN	4067	0	0	0
SLC35B1	10237	0	0	0
ISOC1	51015	0	0	0
TIMP2	7077	0	0	0
DUSP3	1845	0	0	0
LSM6	11157	0	0	0
CALM3	808	0	0	0
MOK	5891	0	0	0
PPP1R13B	23368	0	0	0
SACM1L	22908	−2	0	0
DNAJB2	3300	0	0	0
TCERG1	10915	0	0	0
CERK	64781	0	0	0
KIAA0196	9897	0	0	0
DNM1	1759	0	0	0
LAP3	51056	0	0	0
DDIT4	54541	0	0	0
MAPKAPK5	8550	0	0	0
ALAS1	211	0	4	2
HDGFRP3	50810	0	0	2
PAFAH1B1	5048	0	−2	0

PRKCH	5583	0	0	0	
PSRC1	84722	0	0	0	
RAB21	23011	0	0	0	
ERO1L	30001	0	0	0	
PSIP1	11168	0	0	0	
APOE	348	0	0	0	
CREB1	1385	0	0	0	
MBNL2	10150	0	0	0	
RBM15B	29890	0	0	0	
PACSIN3	29763	0	0	0	
TOMM70A	9868	0	0	0	
CBR1	873	0	0	0	
MTA1	9112	0	0	0	
MACF1	23499	0	0	0	
PRKCD	5580	0	0	0	
CCNA1	8900	0	0	0	
PIK3R4	30849	0	0	0	
CSNK1E	1454	0	0	0	
HIST1H2BK		85236	0	0	0
JUN	3725	0	0	0	
PCCB	5096	0	0	0	
STAP2	55620	0	0	0	
TMEM50A	23585	0	0	0	
PMM2	5373	0	0	0	
SCCPDH	51097	2	0	0	

HSD17B11	51170	0	0	0
TSC22D3	1831	0	0	0
FBXL12	54850	0	0	0
TFAP2A	7020	0	0	0
WDR61	80349	0	0	0
ZNF395	55893	0	0	0
FAS	355	0	0	0
VPS72	6944	0	0	0
CENPE	1062	0	0	0
CLSTN1	22883	0	0	0
CREG1	8804	0	0	0
MAPK9	5601	0	0	0
PRR7	80758	0	0	0
SKP1	6500	0	0	0
POP4	10775	0	0	0
TMEM110	375346	0	0	0
NFE2L2	4780	0	0	0
ZNF589	51385	0	0	0
BAX	581	0	0	0
BLCAP	10904	0	0	0
CNDP2	55748	0	0	0
FAM20B	9917	0	0	0
PROS1	5627	0	0	0
TNIP1	10318	0	0	0
CRKL	1399	0	-2	0

P4HTM	54681	0	0	0
REEP5	7905	0	0	0
ZNF318	24149	0	0	0
BZW2	28969	0	0	0
FDFT1	2222	0	0	2
CGRRF1	10668	0	0	0
TRAP1	10131	2	−3	0
AMDHD2	51005	0	0	0
GTF2A2	2958	0	0	0
PEX11A	8800	0	0	0
PSMD9	5715	0	0	0
GFOD1	54438	0	0	0
MTFR1	9650	0	0	0
PECR	55825	0	0	0
UBE2A	7319	0	0	0
NIT1	4817	0	0	0
SOCS2	8835	0	0	0
SYNGR3	9143	0	0	0
ZDHHC6	64429	0	0	0
ITGAE	3682	0	0	0
NFKB2	4791	0	0	0
STK25	10494	0	0	−2
TMEM5	10329	0	0	0
ABCF3	55324	0	0	0
ATP5S	27109	0	0	0

FCHO1	23149	0	0	0
NGRN	51335	0	0	0
HOXA5	3202	0	−2	0
OXSR1	9943	0	0	0
GRN	2896	0	0	0
SMARCC1	6599	0	0	0
NUP85	79902	0	0	0
PAK6	56924	0	0	0
GHR	2690	0	0	0
GTPBP8	29083	0	0	0
GLOD4	51031	0	0	0
PTK2	5747	0	0	0
CDK4	1019	0	0	0
EPN2	22905	0	0	0
CCDC90A	63933	0	0	0
IL4R	3566	0	0	0
KIAA0528	9847	0	0	0
PRKAG2	51422	0	0	0
KIT	3815	0	0	0
MRPS16	51021	0	0	0
PTPRC	5788	0	0	0
RPS5	6193	0	0	0
NOL3	8996	0	0	0
PSMG1	8624	0	0	0
NR1H2	7376	0	0	0

TMEM97	27346	0	0	0
LRRC41	10489	0	0	0
PLS1	5357	0	0	0
DUSP22	56940	0	0	0
KIAA1279	26128	0	0	0
RPS6KA1	6195	0	0	0
TCEAL4	79921	0	0	0
DCK	1633	0	0	0
NUP133	55746	0	0	2
NUP62	23636	0	0	0
TRIM2	23321	2	0	0
SNCA	6622	0	0	0
SPTAN1	6709	0	0	0
MALT1	10892	0	0	0
VPS28	51160	0	0	0
LRRC16A	55604	0	0	0
YKT6	10652	0	0	0
CHP	11261	0	0	0
KIF2C	11004	0	0	0
NNT	23530	0	0	0
NR2F6	2063	0	0	0
COL1A1	1277	0	0	0
EXOSC4	54512	0	0	0
CDC25A	993	0	0	0
PDLIM1	9124	0	0	0

CRELD2	79174	0	0	0
TP53	7157	0	0	2
IQGAP1	8826	0	0	0
RRP12	23223	0	0	0
ACAT2	39	3	0	2
EGR1	1958	0	0	0
MUC1	4582	0	0	0
THAP11	57215	0	0	0
AURKB	9212	0	0	0
CEBPD	1052	2	0	0
HSPA4	3308	0	0	0
MAST2	23139	0	0	0
DHDDS	79947	0	0	0
TOP2A	7153	0	0	0
GADD45B	4616	0	2	0
NCK2	8440	0	0	0
AURKA	6790	0	−2	0
KIAA0355	9710	0	0	0
B3GNT1	11041	0	0	0
DYNLT3	6990	0	0	0
CDK5R1	8851	0	0	0
CHMP4A	29082	0	0	0
IPO13	9670	−2	0	0
SCP2	6342	0	0	0
ITFG1	81533	0	0	0

RAD9A	5883	0	0	0
DRAP1	10589	0	0	0
SPP1	6696	0	2	0
HYOU1	10525	0	0	0
PCMT1	5110	0	0	0
FOS	2353	0	0	0
FYN	2534	0	0	0
MCOLN1	57192	−2	0	0
MYC	4609	0	0	0
ID2	3398	0	0	0
POLB	5423	0	0	0
CETN3	1070	0	0	0
KCNK1	3775	0	0	0
AXIN1	8312	0	0	0
SMC4	10051	0	0	0
RAI14	26064	0	0	0
SMARCD2	6603	0	0	0
DNAJC15	29103	0	0	0
MPZL1	9019	0	0	0
HOXA10	3206	0	0	0
NRAS	4893	0	0	0
CASP2	835	0	−2	0
EBNA1BP2	10969	2	0	2
CTNND1	1500	0	0	0
TATDN2	9797	−2	0	0

LRPAP1	4043	0	0	−2
ZNF131	7690	0	0	0
BNIP3L	665	0	0	0
HOOK2	29911	0	0	0
ERBB2	2064	0	0	0
TBPL1	9519	0	0	0
INTS3	65123	0	0	0
RAE1	8480	0	2	0
CDK1	983	0	0	0
RFNG	5986	0	0	0
MICALL1	85377	0	0	0
SLC35A1	10559	0	−3	0
KLHL21	9903	−2	0	0
NIPSNAP1	8508	0	0	0
BCL7B	9275	0	0	0
TLE1	7088	0	0	0
FZD7	8324	0	0	0
TIMELESS	8914	0	0	0
COG2	22796	0	0	0
DHX29	54505	−4	0	0
MMP2	4313	0	0	0
MRPS2	51116	0	0	0
KCTD5	54442	0	0	0
TCEA2	6919	0	0	0
CNPY3	10695	0	0	0

FHL2	2274	0	2	0
ADAM10	102	0	0	3
FKBP4	2288	0	2	0
CD58	965	0	0	0
KAT6A	7994	0	0	0
CDK7	1022	0	0	0
KIF5C	3800	0	0	0
HMG20B	10362	0	−2	0
MAP3K4	4216	0	0	0
PIN1	5300	0	0	0
ZFP36	7538	0	0	0
INPP1	3628	0	0	0
LAGE3	8270	0	0	0
RAC2	5880	0	0	0
TNFRSF21	27242	0	0	0
HMOX1	3162	0	6	2
KIF14	9928	0	0	0
COL4A1	1282	2	0	0
DUSP6	1848	0	0	0
CYB561	1534	0	0	0
EPB41L2	2037	0	0	0
GNAI2	2771	0	0	0
HTRA1	5654	0	0	0
TSTA3	7264	0	0	0
WASF3	10810	0	0	0

DNMT1	1786	0	0	0
TSPAN6	7105	0	0	0
FBXO21	23014	0	0	0
RAB27A	5873	0	0	0
DDR1	780	0	0	0
PPP2R3C	55012	0	0	0
ADRB2	154	0	−2	0
LPGAT1	9926	0	0	0
DUSP11	8446	0	0	0
GRB10	2887	2	0	0
MMP1	4312	0	3	2
TP53BP2	7159	0	0	0
CDKN1A	1026	0	−2	0
ZW10	9183	0	0	0
CORO1A	11151	0	0	0
TJP1	7082	0	0	0
ABCB6	10058	0	0	0
XPNPEP1	7511	0	0	0
MAMLD1	10046	0	0	0
MTHFD2	10797	0	0	0
DSG2	1829	0	0	0
PRAF2	11230	0	0	0
DNTTIP2	30836	0	0	0
SLC27A3	11000	0	2	0
CD97	976	2	0	0

HTATSF1	27336	0	−2	−2
IL13RA1	3597	2	0	0
NCAPD2	9918	0	0	0
AGL	178	0	0	0
TMEM109	79073	0	0	0
CDC25B	994	0	0	0
ICAM1	3383	0	0	0
GATA3	2625	0	0	0
NUP93	9688	0	0	2
PLA2G15	23659	0	0	−2
RPA3	6119	0	0	2
PIP4K2B	8396	0	−2	0
TES	26136	0	0	0
MYCBP2	23077	0	0	0
SPR	6697	0	0	0
PGRMC1	10857	0	0	0
RELB	5971	0	0	0
AARS	16	0	0	0
EPHB2	2048	0	0	0
GPC1	2817	0	0	0
RPIA	22934	0	−2	0
ELAC2	60528	0	0	0
GPR56	9289	0	0	0
NVL	4931	0	0	0
PLSCR1	5359	0	0	0

CHEK1	1111	0	0	2
CTSL1	1514	0	0	0
CTSD	1509	0	2	0
ME2	4200	0	0	0
ARHGEF2	9181	0	0	0
PDGFA	5154	0	0	0
ILK	3611	0	0	0
WDR67	93594	0	0	0
NFIL3	4783	0	0	0
RFC2	5982	0	0	0
ARL4C	10123	0	0	0
TIMM9	26520	0	0	0
CPNE3	8895	0	0	0
ORC1	4998	0	0	0
C5	727	0	0	0
PARP2	10038	0	0	0
FASTKD5	60493	0	0	0
SCRN1	9805	0	0	0
MAN2B1	4125	0	0	0
PTPLAD1	51495	0	−2	0
MSRA	4482	0	0	0
NFKBIA	4792	0	0	0
DCTD	1635	0	4	0
SYNE2	23224	0	0	0
MBNL1	4154	0	0	0

UBE3C	9690	0	0	0
CCDC92	80212	0	0	0
MYCBP	26292	0	0	0
CXCL2	2920	0	0	0
PTPN1	5770	0	0	0
BRP44	25874	0	0	0
TIMM22	29928	0	0	0
ATF5	22809	0	0	0
ECD	11319	0	0	0
CIRBP	1153	0	0	0
NSDHL	50814	0	0	0
ADI1	55256	0	0	0
NFKBIE	4794	0	0	0
ADCK3	56997	0	0	0
PRSS23	11098	0	0	2
CCNH	902	0	2	0
GDPD5	81544	0	0	0
P4HA2	8974	0	0	0
TARBP1	6894	0	0	0
ATP6V0B	533	0	0	0
RBKS	64080	0	0	0
CAPN1	823	0	0	0
KDM3A	55818	0	0	0
BID	637	0	0	0
STXBP1	6812	0	0	0

ITGB5	3693	0	0	0
MTF2	22823	0	0	0
HLA.DRA	#N/A	0	0	0
SLC11A2	4891	0	0	0
PDIA5	10954	0	0	0
PIK3C2B	5287	0	−2	0
RAP1GAP	5909	0	0	0
SLC25A46	91137	0	0	0
CDK19	23097	−2	−3	0
NOLC1	9221	0	0	0
CAT	847	0	0	0
KIAA0753	9851	0	0	0
DUSP14	11072	0	0	0
KAT6B	23522	0	0	0
CHN1	1123	0	0	0
HMGA2	8091	0	0	0
IGFBP3	3486	0	0	0
MAPK13	5603	0	0	0
TRIB1	10221	0	0	0
UFM1	51569	0	0	0
GNA15	2769	0	0	0
TP53BP1	7158	0	0	0
FRS2	10818	−2	0	0
IARS2	55699	0	0	0
BECN1	8678	0	0	0

GATA2	2624	0	0	0
KDELR2	11014	0	0	0
RALGDS	5900	0	0	0
ELAVL1	1994	0	0	0
NRIP1	8204	−2	0	0
CCNB2	9133	0	0	0
IFNAR1	3454	0	0	0
MIF	4282	0	0	0
RNPS1	10921	0	0	0
DAG1	1605	0	0	0
SATB1	6304	0	0	0
ARID4B	51742	0	0	0
IGF2BP2	10644	0	0	0
GSTZ1	2954	2	−2	0
IGF2R	3482	0	0	0
SESN1	27244	0	0	0
TIPARP	25976	0	−2	0
CRTAP	10491	−2	0	0
SPEN	23013	0	0	0
GMNN	51053	0	0	0
PFKL	5211	0	0	0
CYTH1	9267	0	−2	0
PDHX	8050	0	0	0
DENND2D	79961	0	−3	0
PRCP	5547	0	0	0

CTTN	2017	−2	0	0
SMARCA4	6597	0	0	0
PYGL	5836	0	0	0
UBR7	55148	0	0	0
GRB7	2886	0	0	0
RPA2	6118	0	0	0
CD44	960	0	0	0
ERBB3	2065	0	0	0
GTF2E2	2961	0	0	0
VAV3	10451	0	0	0
ABHD6	57406	0	0	0
ANXA7	310	0	0	0
MAPKAPK2	9261	0	2	0
TXNRD1	7296	0	0	0
CAB39	51719	0	0	0
FZD1	8321	0	0	0
CRYZ	1429	0	2	0
TIAM1	7074	0	0	0
DNM1L	10059	0	0	0
GAA	2548	0	−3	0
DERA	51071	0	0	0
SKIV2L	6499	0	0	0
BLMH	642	0	0	0
PTPRK	5796	0	0	0
HERC6	55008	0	0	0

RRP1B	23076	0	0	0
MKNK1	8569	0	0	0
TSPAN3	10099	0	0	0
ACOT9	23597	0	0	2
PIK3R3	8503	0	0	0
KEAP1	9817	0	2	0
ST3GAL5	8869	0	0	0
CTNNAL1	8727	0	0	0
TFDP1	7027	0	0	0
ABHD4	63874	0	0	0
SMC3	9126	0	0	2
INPP4B	8821	0	0	2
LBR	3930	0	0	0
PPIE	10450	0	0	0
ZMIZ1	57178	0	0	0
PPARG	5468	0	0	0
TERF2IP	54386	0	−2	0
BNIP3	664	0	0	0
RPP38	10557	0	0	0
BCL2	596	0	0	0
CAST	831	0	0	0
BAG3	9531	0	3	0
RBM6	10180	0	0	0
CSK	1445	0	0	0
GNA11	2767	0	0	0

ALDOC	230	0	0	2
TOR1A	1861	0	0	0
SLC25A13	10165	0	0	0
UBE2L6	9246	0	0	0
SYK	6850	0	2	0
TSPAN4	7106	0	−2	−2
NR3C1	2908	0	0	0
PCBD1	5092	0	0	0
ICMT	23463	0	−2	0
TRIB3	57761	0	2	0
EIF4EBP1	1978	0	0	0
PHF15	23338	0	0	0
CXCR4	7852	0	0	0
GNPDA1	10007	0	0	0
CCND3	896	0	0	0
FKBP14	55033	0	0	0
CIAPIN1	57019	0	0	0
NUCB2	4925	0	0	0
COG7	91949	0	0	0
RSU1	6251	0	0	0
PTPN6	5777	0	0	0
TUBB6	84617	0	0	0
ABL1	25	0	0	0
DUSP4	1846	0	0	0
CCNF	899	0	−2	0

PTPN12	5782	0	0	0
HSPA1A	3303	0	4	0
PCK2	5106	0	0	0
FAM57A	79850	3	0	2
RB1	5925	0	0	0
EVL	51466	0	−3	0
GFPT1	2673	0	0	0
BLVRA	644	0	0	0
GPER	2852	0	0	0
EAPP	55837	−2	0	0
S100A13	6284	0	0	0
EXT1	2131	0	−3	0
MYBL2	4605	0	0	0
POLE2	5427	0	0	0
STX4	6810	0	3	0
PLCB3	5331	0	0	0
TMCO1	54499	0	0	0
C2CD2	25966	0	0	0
PARP1	142	0	−3	0
ANKRD10	55608	0	0	0
CCDC85B	11007	0	−2	0
DDB2	1643	0	0	0
DDX10	1662	0	0	0
BAMBI	25805	0	0	0
CEP57	9702	0	0	0

HADH	3033	0	0	0
RAB31	11031	0	0	0
ABCC5	10057	0	0	0
GLRX	2745	0	0	0
MVP	9961	0	0	0
RFC5	5985	0	0	0
HES1	3280	0	0	0
STMN1	3925	0	0	0
GALE	2582	0	0	−2
KLHL9	55958	0	0	0
PTPRF	5792	0	0	0
UBE2J1	51465	0	0	0
PGM1	5236	3	0	0
SLC37A4	2542	0	0	0
LOXL1	4016	0	0	0
PLK1	5347	0	0	0
MYO10	4651	0	−2	0
NPRL2	10641	0	−2	0
LIPA	3988	0	−2	0
PWP1	11137	0	0	0
PHGDH	26227	0	0	0
PLEKHM1	9842	0	0	0
ST7	7982	0	0	0
VAT1	10493	0	0	0
IKBKB	3551	0	0	0

KIF20A	10112	0	0	0
CDH3	1001	0	0	0
PCM1	5108	0	0	0
IER3	8870	0	2	0
SSBP2	23635	0	0	0
FAT1	2195	0	0	0
SNX11	29916	0	0	0
RNMT	8731	0	0	0
SFN	2810	0	0	0
EGFR	1956	0	0	0
RFX5	5993	0	0	0
NET1	10276	2	0	0
PKIG	11142	0	0	0
DECR1	1666	0	0	0
FSD1	79187	0	0	0
ATP6V1D	51382	0	0	0
CDK6	1021	0	0	0
CDC45	8318	2	0	0
NPC1	4864	0	0	0
EDEM1	9695	0	0	0
MEST	4232	0	0	0
MYLK	4638	0	0	0
PAFAH1B3	5050	0	0	0
CALU	813	0	0	0
USP6NL	9712	0	0	0

LGALS8	3964	0	2	0
NT5DC2	64943	0	0	0
CCP110	9738	0	0	0
LRP10	26020	0	0	0
ATP1B1	481	0	0	0
STK10	6793	0	−2	−2
OXCT1	5019	0	0	0
UGDH	7358	0	0	0
SLC25A4	291	0	0	0
SQRDL	58472	0	0	0
HEBP1	50865	0	0	0
PSMB10	5699	0	0	0
ASCC3	10973	0	0	0
FGFR4	2264	0	0	0
CDKN2A	1029	0	0	0
TRAM2	9697	0	0	0
ATP11B	23200	0	0	0
PYCR1	5831	0	0	0
CD320	51293	0	−2	0
NCOA3	8202	0	0	0
CDKN1B	1027	0	−2	0
POLD4	57804	0	0	0
FOSL1	8061	0	2	0
MLLT11	10962	0	2	0
CEBPZ	10153	0	0	−2

PRKACA	5566	0	0	0
GAPDH	2597	0	0	0
CBR3	874	0	2	0
LYRM1	57149	0	0	0
