# Supplementary material for: Novel Curcumin Inspired Bis-Chalcone Promotes Endoplasmic Reticulum Stress and Glioblastoma Neurosphere Cell Death
Source: Cancers (Basel). 2019 Mar 13;11(3):357. doi: 10.3390/cancers11030357 (PMC6468769; doi:10.3390/cancers11030357)
Supplement: Supplementary file 1 [file cancers-11-00357-s001.zip › Graham Cancers 444757 supplemental files/Graham Appendix B Bischalcone - experimental.pdf]

Novel *Curcumin Inspir*ed Bis-chalcone promotes Endoplasmic Reticulum Stress and Glioblastoma Neurosphere cell death

Lorenzo Sansalone[1], Eduardo Veliz[1], Nadia Myrthil[2], Vasileios Stathias[2,3,4,5], Winston Walters[2], Ingrid I. Torrens[2], Stephan Schürer[3,5] Steven Vanni[6], Roger M. Leblanc[1] and Regina M. Graham[2,3,6]

Corresponding Author:
Regina M. Graham, Ph.D.
Research Assistant Professor, Department of Neurological Surgery
University of Miami Miller School of Medicine
Lois Pope Life Center, 1095 14th Terrace, Miami, FL 33136
Email: RGraham@med.miami.edu

Experimental Section

General: all reagents were obtained from commercial sources and were used without further purification unless noted otherwise. Thin layer chromatography (TLC) was performed with Merck silica gel 60 F254 pre-coated plates, eluting with the solvents indicated. Short and long wave visualization was performed with a Mineralight multiband ultraviolet lamp at 254 and 365 nm, respectively. ^{1}H and ^{13}C Nuclear Magnetic Resonance spectra of pure compounds were recorded either on AVANCE II 400 or AVANCE 500 (Bruker). Chemical shifts are reported in parts per million in reference to the solvent peak. The abbreviations s, d, dd, t, td, q, m, and brs stand for singlet, doublet, doublet of doublets, triplet, triplet of doublets, quartet, multiplet, and broad singlet, in that order. High resolution mass spectra were obtained on a Bruker micrOTO-Q II spectrometer.

General procedure for the synthesis of bischalcones

Method A: to a stirred solution of substituted benzaldehyde (2.1 equiv.) and 2,6-diacetylpyridine (1 equiv.) in methanol (5 mL) was added a 1:1 mixture of methanol and 20% aq. NaOH (2 mL). The reaction mixture was stirred at room temperature for 24-48 h. The reaction mixture was

diluted with water and the solid obtained was collected by vacuum filtration. The purity of the crude product was determined by TLC analysis and NMR spectroscopy. If needed, the solid was recrystallized from chloroform/methanol co-solvent to give the analytically pure product.

Method B: to a stirred solution of the substituted benzaldehyde (2.1 equiv.) and 2,6-diacetylpyridine (1 equiv.) in methanol (8 mL) was added piperidine (100 μL). The mixture was heated under gentle reflux for 24 h. The reaction mixture was cooled down and water was added to the mixture. The solid obtained was collected by vacuum filtration. The purity of the crude product was determined by TLC analysis and NMR spectroscopy. If needed, the solid was recrystallized from chloroform/methanol co-solvent to give the analytically pure product.

2,6-Di-[3-(2-methoxyphenyl)propenoyl]pyridine (**4b**): the general procedure A was followed to give the product as a light yellow solid with the isolate yield 44%; ^{1}H NMR (500 MHz, CD_2Cl_2) δ (ppm): 8.51 (d, 2H, J = 16 Hz), 8.37 (d, 2H, J = 8 Hz), 8.36 (d, 2H, J = 16.5 Hz), 8.11 (t, 1H, J = 8 Hz), 7.84 (dd, 2H, J = 1.5 Hz, J = 7.5 Hz), 7.46 (td, 2H, J = 1.5 Hz, J = 7.5 Hz), 7.06 (d, 2H, J = 7.5 Hz), 7.02 (d, 2H, J = 8.5 Hz), 3.91 (s, 6H); ^{13}C NMR (125 MHz , CD_2Cl_2) δ (ppm): 188.9, 159.1, 153.4, 139.9, 138.3, 132.1, 128.8, 125.5, 124.1, 121.3, 120.7, 111.4, 55.6. HRMS calculated for $[M+1]^+$ 400.1504, found 400.1554.

2,6-Di-[3-(3-methoxyphenyl)propenoyl]pyridine (**4c**): the general procedure A was followed to give the product as a pale yellow solid with the isolate yield 32%; ^{1}H NMR (500 MHz, CD_2Cl_2) δ (ppm): 8.46 (d, 2H, J = 16 Hz), 8.39 (d, 2H, J = 7.5 Hz), 8.13 (t, 1H, J = 8 Hz), 8.00 (d, 2H, J = 16 Hz), 7.42-7.41 (m, 4H), 7.31 (d, 2H, J = 1.5 Hz), 7.06-7.04 (m, 2H), 3.86 (s, 6H); ^{13}C NMR

(125 MHz, CD_2Cl_2) δ (ppm): 188.4, 160.1, 153.2, 144.6, 138.4, 136.5, 130.0, 125.7, 121.1, 121.0, 116.5, 113.7, 55.3. HRMS calculated for $[M+Na]^+$ 422.1369, found 422.1371.

2,6-Di-[3-(2,3-dimethoxyphenyl)propenoyl]pyridine (**4d**): the general procedure A was followed to give the product as a pale yellow solid with the isolate yield 96%; ^{1}H NMR (500 MHz, $CDCl_3$) δ (ppm): 8.58 (d, 2H, *J* = 16.5 Hz), 8.41 (d, 2H, *J* = 8 Hz), 8.34 (d, 2H, *J* = 16.5 Hz), 8.10 (t, 1H, *J* = 8 Hz), 7.50 (dd, 2H, *J* = 1 Hz, *J* = 8 Hz), 7.16 (t, 2H, *J* = 8 Hz), 7.04 (dd, 2H, *J* = 1 Hz, *J* = 8 Hz), 3.94 (s, 12H); ^{13}C NMR (125 MHz, CD_2Cl_2) δ (ppm): 188.7, 153.4, 153.3, 149.3, 139.3, 138.4, 129.2, 125.6, 124.1, 122.0, 119.6, 114.6, 61.2, 55.8. HRMS calculated for $[M+1]^+$ 460.1715, found 460.1750.

2,6-Di-[3-(3,5-dimethoxyphenyl)propenoyl]pyridine (**4h**):): the general procedure A was followed to give the product as a light beige solid with the isolate yield 66%; ^{1}H NMR (500 MHz, CD_2Cl_2) δ (ppm): 8.39 (d, 2H, *J* = 8 Hz), 8.34 (d, 2H, *J* = 16 Hz), 8.13 (t, 1H, *J* = 8 Hz), 7.95 (d, 2H, *J* = 16 Hz), 6.91 (d, 2H, *J* = 2 Hz), 6.59 (t, 2H, *J* = 2.5 Hz), 3.81 (s, 12H); ^{13}C NMR (125 MHz, CD_2Cl_2) δ (ppm): 188.6, 161.2, 153.3, 145.0, 138.4, 137.0, 125.8, 121.2, 106.4, 102.9, 55.4. HRMS calculated for $[M+1]^+$ 460.1715, found 460.1750.

2,6-Di-[3-(3,4-methylenedioxyphenyl)propenoyl]pyridine (**4i**): the general procedure A was followed to give the product as a yellow solid with the isolate yield 67%; ^{1}H NMR (500 MHz, $CDCl_3$) δ (ppm): 8.39 (d, 2H, *J* = 7.5 Hz), 8.31 (d, 2H, *J* = 16 Hz), 8.09 (t, 1H, *J* = 8 Hz), 8.00 (d, 2H, *J* = 15.5 Hz), 7.31 (dd, 2H, *J* = 1.5 Hz, *J* = 8 Hz), 6.93 (d, 2H, *J* = 8 Hz), 6.08 (s, 4H); ^{13}C

NMR (125 MHz, CD_2Cl_2) δ (ppm): 188.5, 153.3, 150.2, 148.5, 144.9, 129.7, 125.7, 125.0, 118.8, 108.9, 107.4. HRMS calculated for $[M+1]^+$ 428.1089, found 428.1145.

2,6-Di-[3-(3,4,5-trimethoxyphenyl)propenoyl]pyridine (**4j**): the general procedure B was followed to give the product as a mellow yellow solid with the isolate yield 27%; ^1H NMR (500 MHz, CD_2Cl_2) δ (ppm): 8.36 (d, 2H, J = 8 Hz), 8.18 (d, 2H, J = 16 Hz), 8.14 (t, 1H, J = 7.5 Hz), 7.99 (d, 2H, J = 15.5 Hz), 6.96 (s, 4H), 3.88 (s, 6H), 3.78 (s, 12H); ^{13}C NMR (125 MHz, CD_2Cl_2) δ (ppm): 188.7, 153.7, 153.3, 145.3, 140.9, 138.5, 130.6, 125.7, 120.7, 105.9, 60.6, 56.0. HRMS calculated for $[M+1]^+$ 520.1927, found 520.1956.

2,6-Di-[3-(4-methoxymethoxyphenyl)propenoyl]pyridine (**4j**): the general procedure B was followed to give the product as a light yellow solid with the isolate yield 55%; ^1H NMR (500 MHz, CD_2Cl_2) δ (ppm): 8.40 (d, 2H, J = 16 Hz), 8.37 (d, 2H, J = 8 Hz), 8.11 (t, 1H, J = 7.5 Hz), 8.00 9(d, 2H, J = 16 Hz), 7.78 (m, 4H), 7.16 (d, 4H, J = 9 Hz), 5.29 (s, 4H), 3.53 (s, 6H); ^{13}C NMR (125 MHz, $CDCl_3$) δ (ppm): 188.7, 159.5, 153.3, 144.7, 138.3, 130.4, 129.0, 125.7, 119.0, 116.6, 94.2, 56.2. HRMS calculated for $[M+1]^+$ 460.1715, found 460.1778.

2,6-Di-[3-(3-methoxy-4-methoxymethoxyphenyl)propenoyl]pyridine (**4m**): the general procedure B was followed to give the product as a yellow solid with the isolate yield 56%; ^1H NMR (500 MHz, CD_2Cl_2) δ (ppm): 8.37 (d, 2H, J = 8 Hz), 8.30 (d, 2H, J = 16 Hz), 8.12 (t, 1H, J = 7.5 Hz), 7.99 (d, 2H, J = 16 Hz), 7.38 (dd, 2H, J = 2 Hz, J = 8.5 Hz), 7.29 (d, 2H, J = 2 Hz), 7.18 (d, 2H, J = 8.5 Hz), 5.29 (s, 4H), 3.86 (s, 6H), 3.54 (s, 6H); ^{13}C NMR (125 MHz, CD_2Cl_2) δ (ppm): 188.4, 153.4, 150.1, 149.1, 144.9, 138.4, 129.5, 125.7, 122.2, 119.2, 116.1, 111.7, 95.2, 56.2, 55.8. HRMS calculated for $[M+1]^+$ 520.1927, found 520.2003.

2,6-Di-[3-(4-benzyloxy-3-methoxyphenyl)propenoyl]pyridine (**4o**): the general procedure A was followed to give the product as a yellow solid with the isolate yield 19%; ^{1}H NMR (500 MHz, CD_2Cl_2) δ (ppm): 8.37 (d, 2H, J = 7.5 Hz), 8.28 (d, 2H, J = 16 Hz), 8.12 (t, 1H, J = 8 Hz), 8.00 (d, 2H, J = 16 Hz), 7.49 (d, 4H, J = 7 Hz), 7.45-7.42 (m, 4H), 7.40-7.38 (m, 2H), 7.36 (dd, 2H, J = 1.5 Hz, J = 8 Hz), 7.31 (d, 2H, J = 2 Hz), 7.01 (d, 2H, J = 8 Hz), 5.18 (s, 4H), 3.85 (s, 6H); ^{13}C NMR (125 MHz, CD_2Cl_2) δ (ppm): 188.5, 153.5, 151.0, 149.9, 145.0, 138.3, 136.6, 128.6, 128.5, 128.2, 127.7, 125.6, 122.7, 118.9, 113.2, 111.5, 70.9, 55.9. HRMS calculated for $[M+1]^+$ 612.2341, found 612.2378.

2,6-Di-[5-(4-dimethylaminophenyl)pent-2,4-dienoyl]pyridine (**4q**): the general procedure B was followed to give the product as a maroon solid with the isolate yield 59%; ^{1}H NMR (500 MHz, CD_2Cl_2) δ (ppm): 8.31 (d, 2H, J = 7.5 Hz), 8.06 (t, 1H, J = 7.5 Hz), 7.85-7.83 (m, 4H), 7.51 (d, 4H, J = 9 Hz), 7.11-7.10 (m, 4H), 6.75 (d, 4H, J = 8.5 Hz), 3.06 (s, 12H); ^{13}C NMR (125 MHz, CD_2Cl_2) δ (ppm): 188.4, 153.7, 151.4, 146.5, 143.7, 138.0, 129.0, 125.1, 124.1, 122.8, 121.3, 111.9, 39.9. HRMS calculated for $[M+1]^+$ 478.2450, found 478.2502.

2,6-Di-[3-(2,3,4-trimethoxyphenyl)propenoyl]pyridine (**4r**): the general procedure B was followed to give the product as a yellow solid with the isolate yield 59%; ^{1}H NMR (500 MHz, CD_2Cl_2) δ (ppm): 8.50 (d, 2H, J = 16 Hz), 8.37 (d, 2H, J = 7.5 Hz), 8.18 (d, 2H, J = 16.5 Hz), 8.11 (t, 1H, J = 8 Hz), 7.60 (d, 2H, J = 9 Hz), 6.82 (d, 2H, J = 9 Hz,), 3.99 (s, 6H), 3.95 (s, 6H), 3.91 (s, 6H); ^{13}C NMR (125 MHz, CD_2Cl_2) δ (ppm): 188.8, 156.2, 154.1, 153.6, 142.6, 140.0, 138.2, 125.4, 124.2, 122.1, 119.9, 107.7, 61.4, 60.7, 56.0. HRMS calculated for $[M+1]^+$ 520.1927, found 520.1987.

2,6-Di-[3-(2,4,5-trimethoxyphenyl)propenoyl]pyridine (**4s**): the general procedure B was followed to give the product as an orange solid with the isolate yield 80%; ^{1}H NMR (500 MHz, CD_2Cl_2) δ (ppm): 8.35 (d, 2H, J = 16.5 Hz,), 8.32 (s, 1H), 8.10 (t, 1H, J = 7.5 Hz), 7.26 (s., 2H), 6.57 (s, 2H), 3.95 (s, 6H), 3.87 (s, 6H), 3.73 (s, 6H); ^{13}C NMR (125 MHz, CD_2Cl_2) δ (ppm): 189.0, 155.0, 153.8, 153.2, 143.5, 140.2, 138.1, 125.3, 119.1, 115.7, 111.9, 97.0, 56.5, 56.3, 56.0. HRMS calculated for $[M+1]^+$ 520.1927, found 520.1990.

2,6-Di-[3-(4-hydroxy-3-methoxyphenyl)propenoyl]pyridine (**4n**): to a solution of the protected bischalcone **4m** (250 mg) in dichloromethane (15 mL) was added trifluoroacetic acid (15 mL) and the mixture was stirred at room temperature for 2 h. Then, the reaction mixture was concentrated and the oil obtained was diluted with water (10 mL). The aqueous solution was cooled down and neutralized with a saturated aqueous solution of sodium bicarbonate. The solid obtained was collected by vacuum filtration. Recrystallization in $CHCl_3/CH_3OH$ co-solvent afforded the product as a mustard color solid. Yield: 18%. ^{1}H NMR (400 MHz, $CDCl_3$) δ (ppm): 8.38 (d, 2H, J = 9.5 Hz), 8.22 (d, 2H, J = 20 Hz,), 8.08 (t, 1H, J = 9.5 Hz), 8.00 (d, 2H, J = 20 Hz), 7.34 (d, 2H, J = 10.5 Hz), 7.20 (s, 2H), 6.97 (d, 2H, J = 10.5 Hz), 3.87 (s, 6H); ^{13}C NMR (100 MHz, $CDCl_3$) δ (ppm): 188.7, 153.4, 148.6, 146.9, 145.6, 127.8, 125.8, 123.2, 118.6, 114.9, 110.5, 55.9. HRMS calculated for $[M+1]^+$ 432.1402, found 432.1435.

Spectral Data

SpinWorks 4: 2,6-Di-[3-(2-methoxyphenyl)propenoyl]pyridine

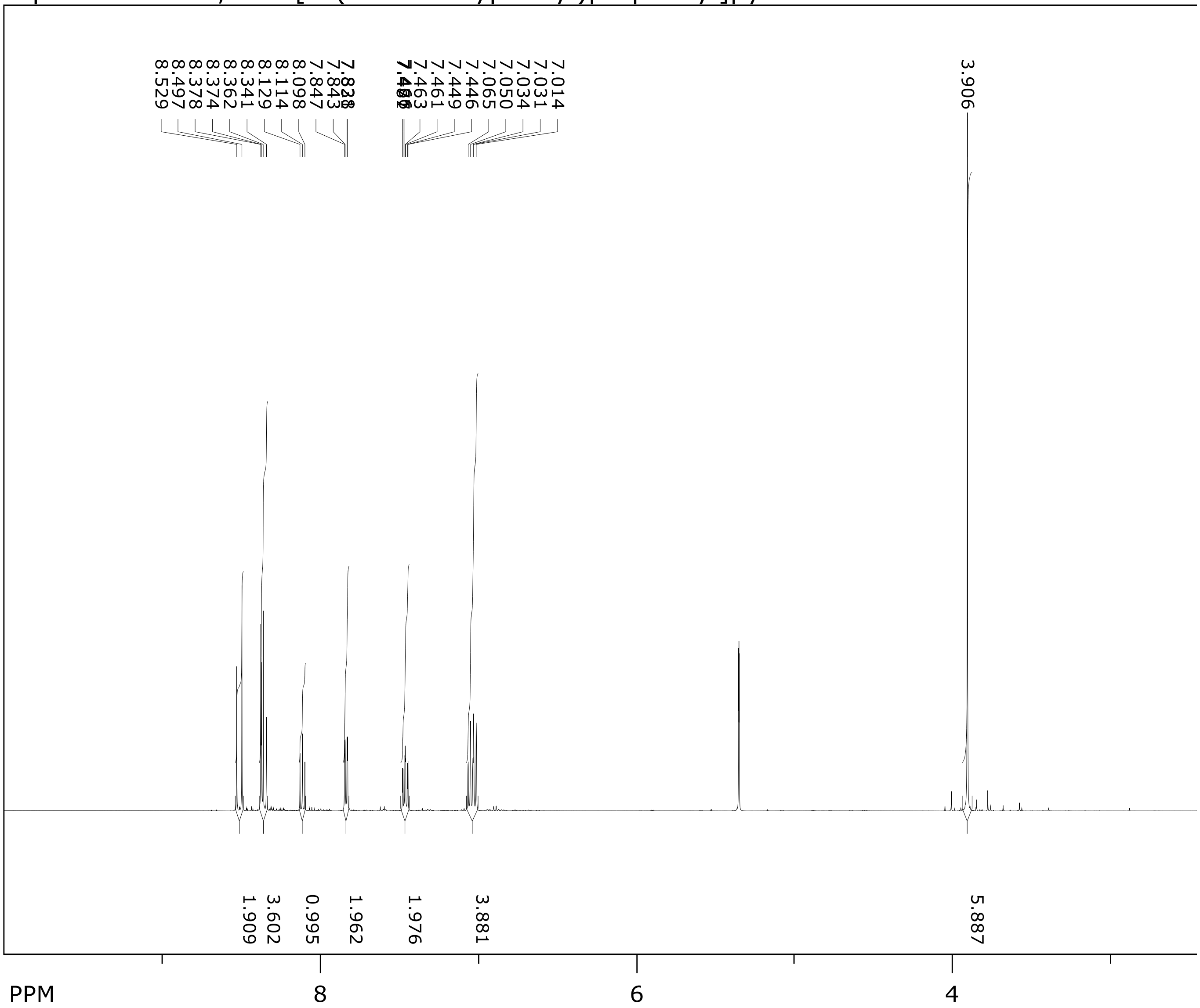

file: ...h\NMR Data\EAV\500NMR\LS-147\1\fid expt: <zg30>
transmitter freq.: 500.063088 MHz
time domain size: 65536 points
width: 10330.58 Hz = 20.6586 ppm = 0.157632 Hz/pt
number of scans: 64

freq. of 0 ppm: 500.060000 MHz
processed size: 32768 complex points
LB: 0.300 GF: 0.0000

SpinWorks 4: 2,6-Di-[3-(2-mehoxyphenyl)propenoyl]pyridine

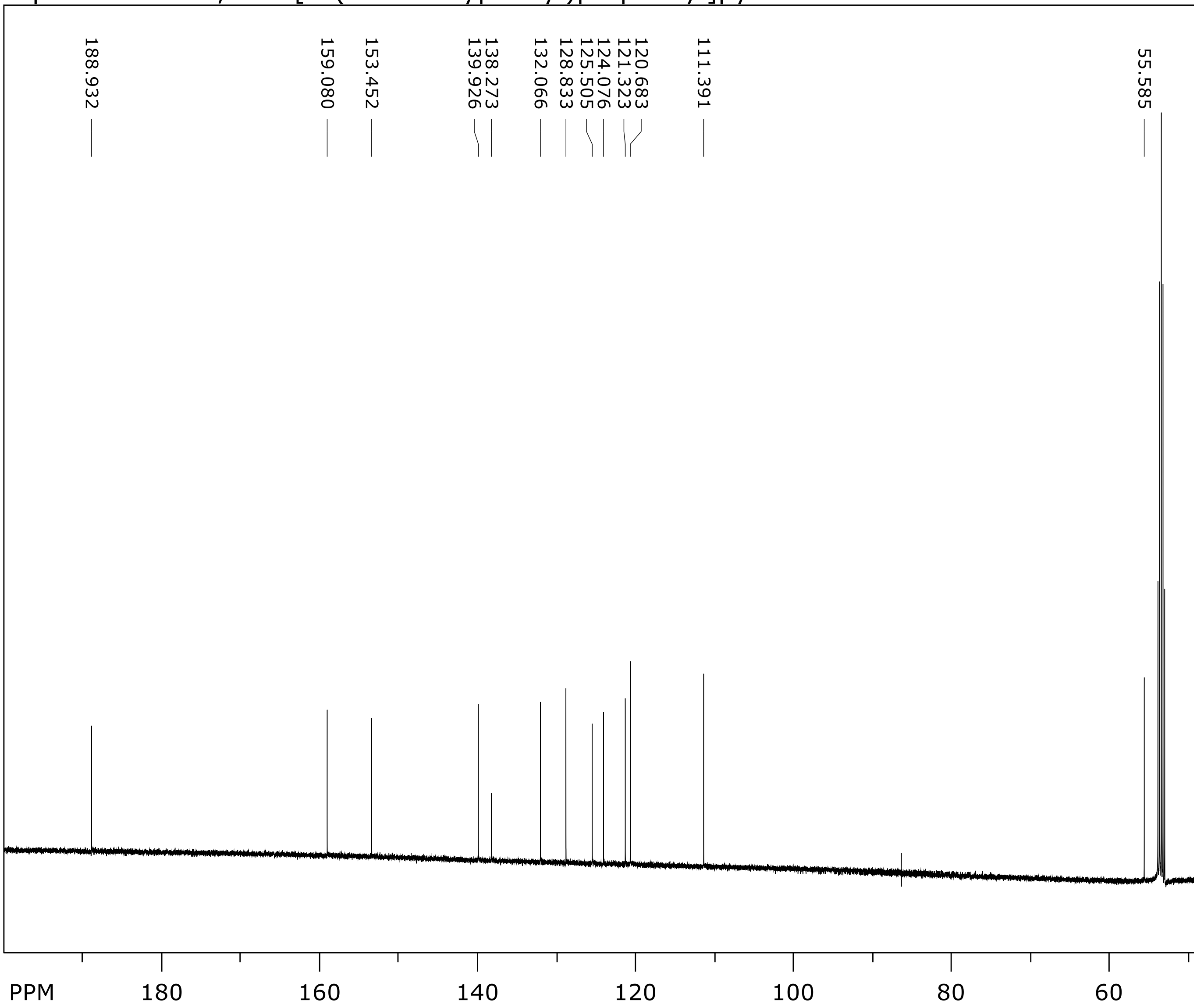

file: ...\NMR Data\EAV\500NMR\LS-147C\1\fid expt: <zgpg>
transmitter freq.: 125.752761 MHz
time domain size: 262144 points
width: 26455.03 Hz = 210.3733 ppm = 0.100918 Hz/pt
number of scans: 597

freq. of 0 ppm: 125.740187 MHz
processed size: 131072 complex points
LB: 0.300 GF: 0.0000

SpinWorks 4: 2,6-Di-[3-(3-methoxyphenyl)propenoyl]pyridine

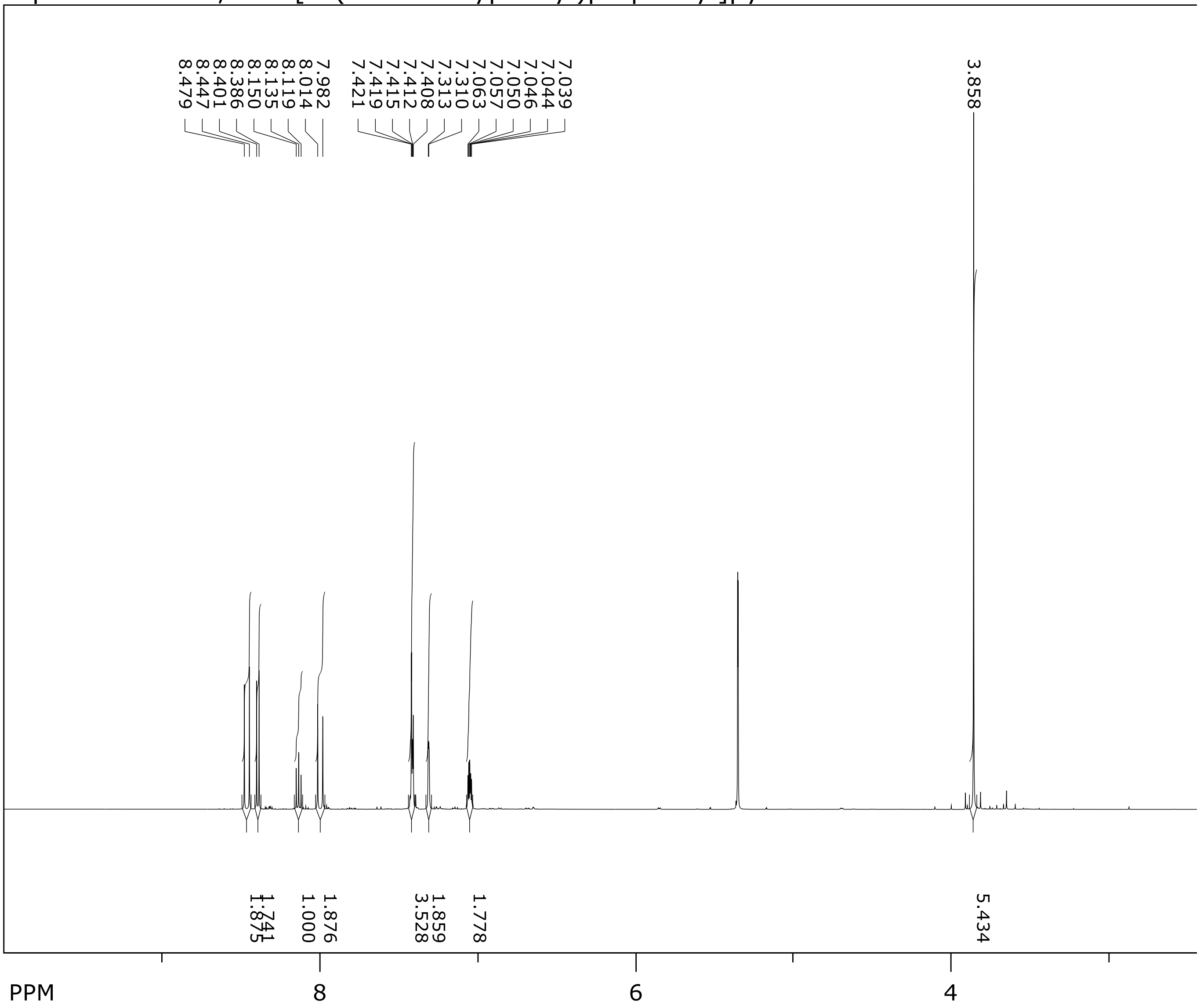

file: ...h\NMR Data\EAV\500NMR\LS-312\1\fid expt: <zg30>
transmitter freq.: 500.063088 MHz
time domain size: 65536 points
width: 10330.58 Hz = 20.6586 ppm = 0.157632 Hz/pt
number of scans: 64

freq. of 0 ppm: 500.060000 MHz
processed size: 32768 complex points
LB: 0.300 GF: 0.0000

SpinWorks 4: 2,6-Di-[3-(3-mehoxyphenyl)propenoyl]pyridine

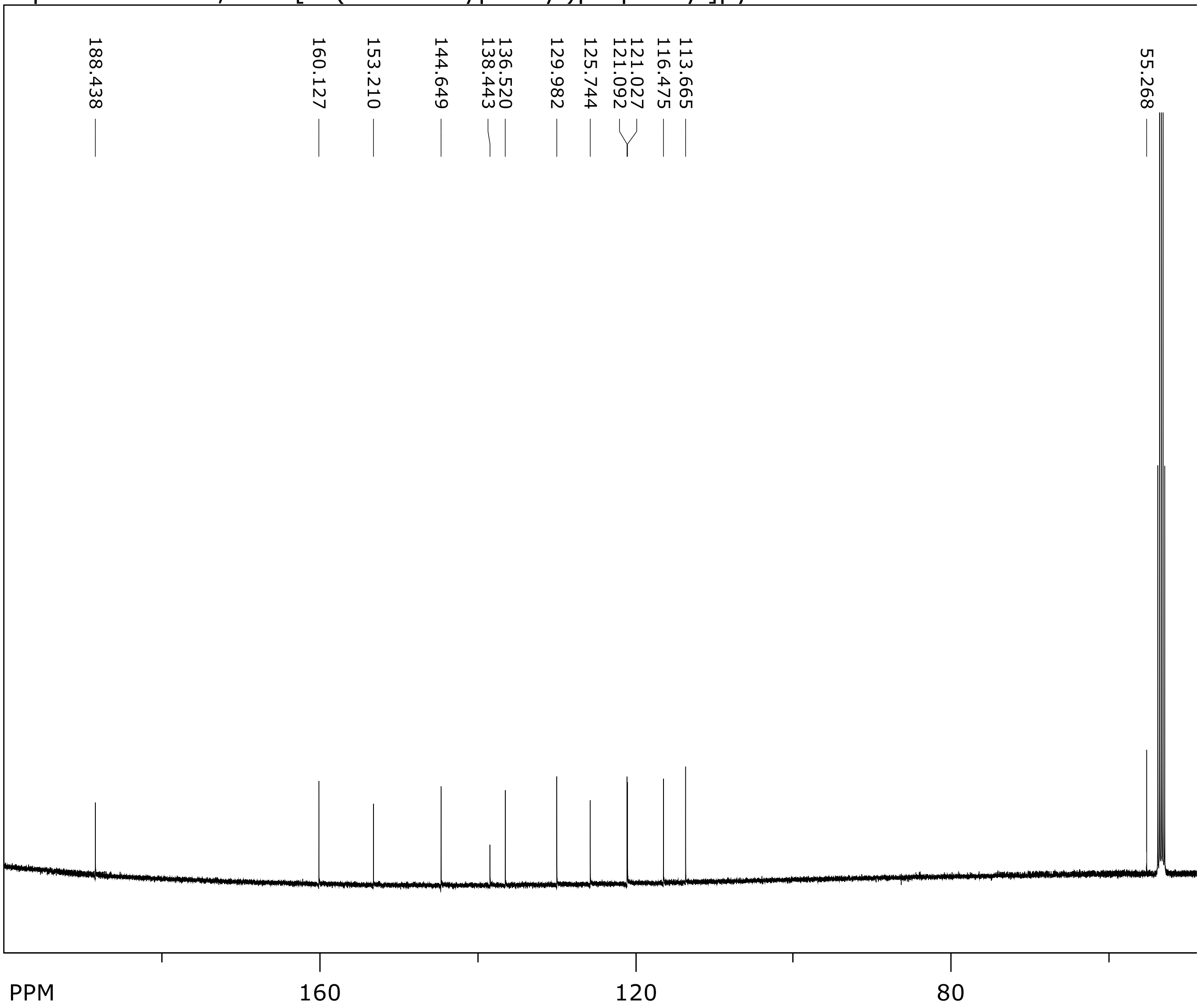

file: ...\NMR Data\EAV\500NMR\LS-312C\1\fid expt: <zgpg>
transmitter freq.: 125.752761 MHz
time domain size: 262144 points
width: 26455.03 Hz = 210.3733 ppm = 0.100918 Hz/pt
number of scans: 1416

freq. of 0 ppm: 125.740187 MHz
processed size: 131072 complex points
LB: 0.300 GF: 0.0000

SpinWorks 4: 2,6-Di-[3-(2,3-dimethoxyphenyl)propenoyl]pyridine

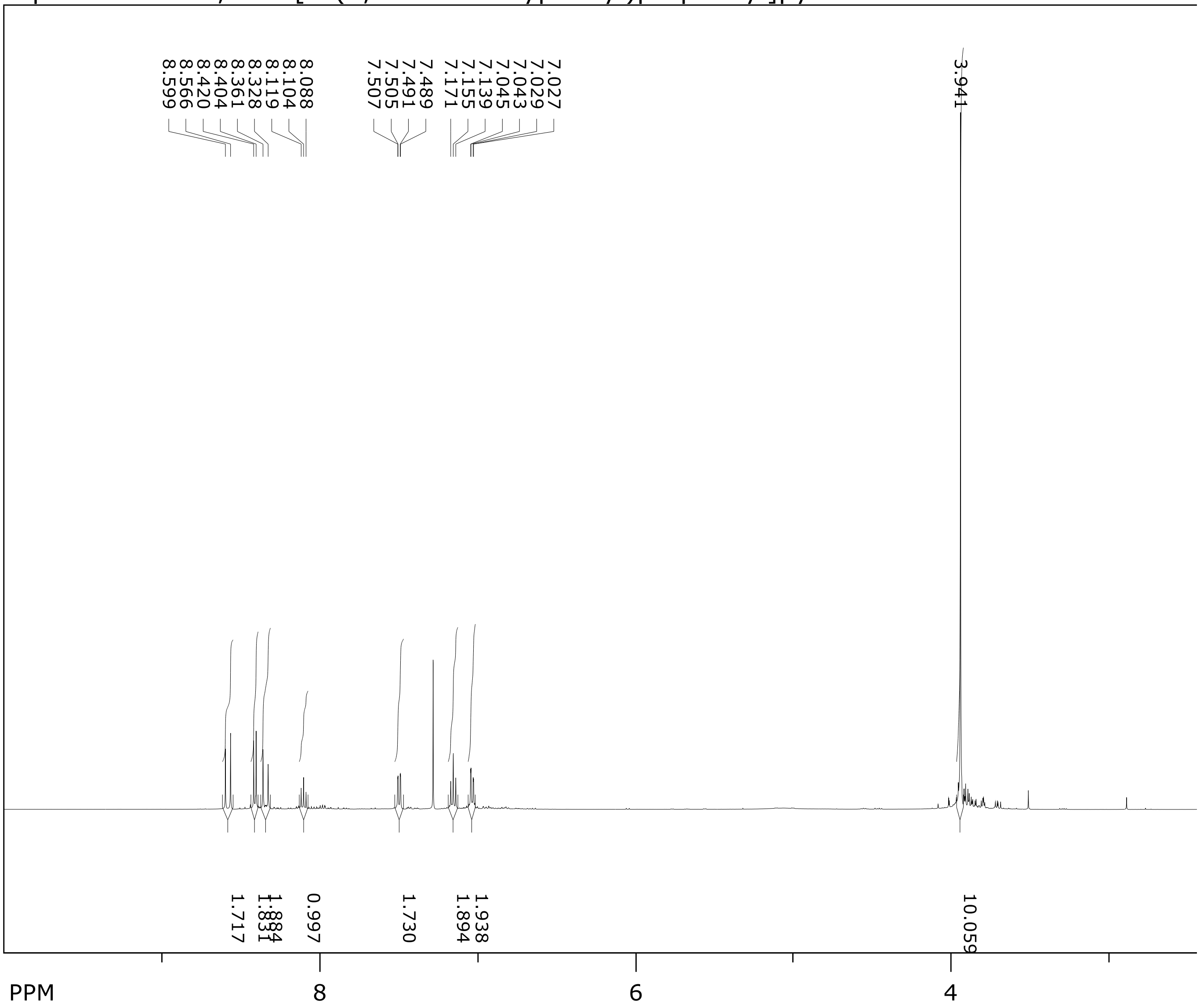

file: ... Data\EAV\500NMR\EAV-VII-120\1\fid expt: <zg30>
transmitter freq.: 500.063088 MHz
time domain size: 65536 points
width: 10330.58 Hz = 20.6586 ppm = 0.157632 Hz/pt
number of scans: 64

freq. of 0 ppm: 500.060000 MHz
processed size: 32768 complex points
LB: 0.300 GF: 0.0000

SpinWorks 4: 2,6-Di-[3-(2,3-dimehoxyphenyl)propenoyl]pyridine

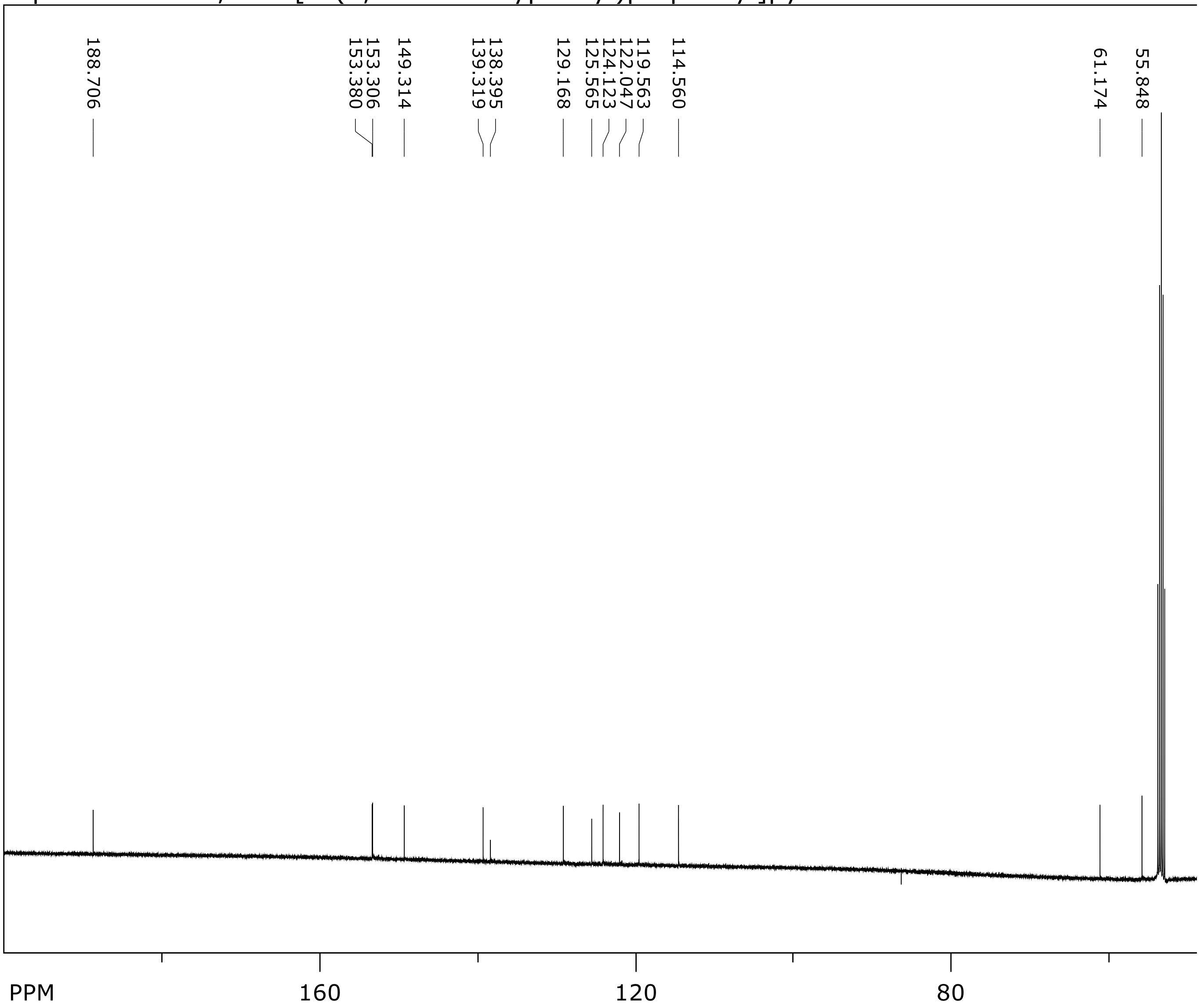

file: ...Data\EAV\500NMR\EAV-VII-120C\1\fid expt: <zgpg>
transmitter freq.: 125.752761 MHz
time domain size: 262144 points
width: 26455.03 Hz = 210.3733 ppm = 0.100918 Hz/pt
number of scans: 1288

freq. of 0 ppm: 125.740187 MHz
processed size: 131072 complex points
LB: 0.300 GF: 0.0000

SpinWorks 4: 2,6-Di-[3-(3,5-dimethoxyphenyl)propenoyl]pyridine

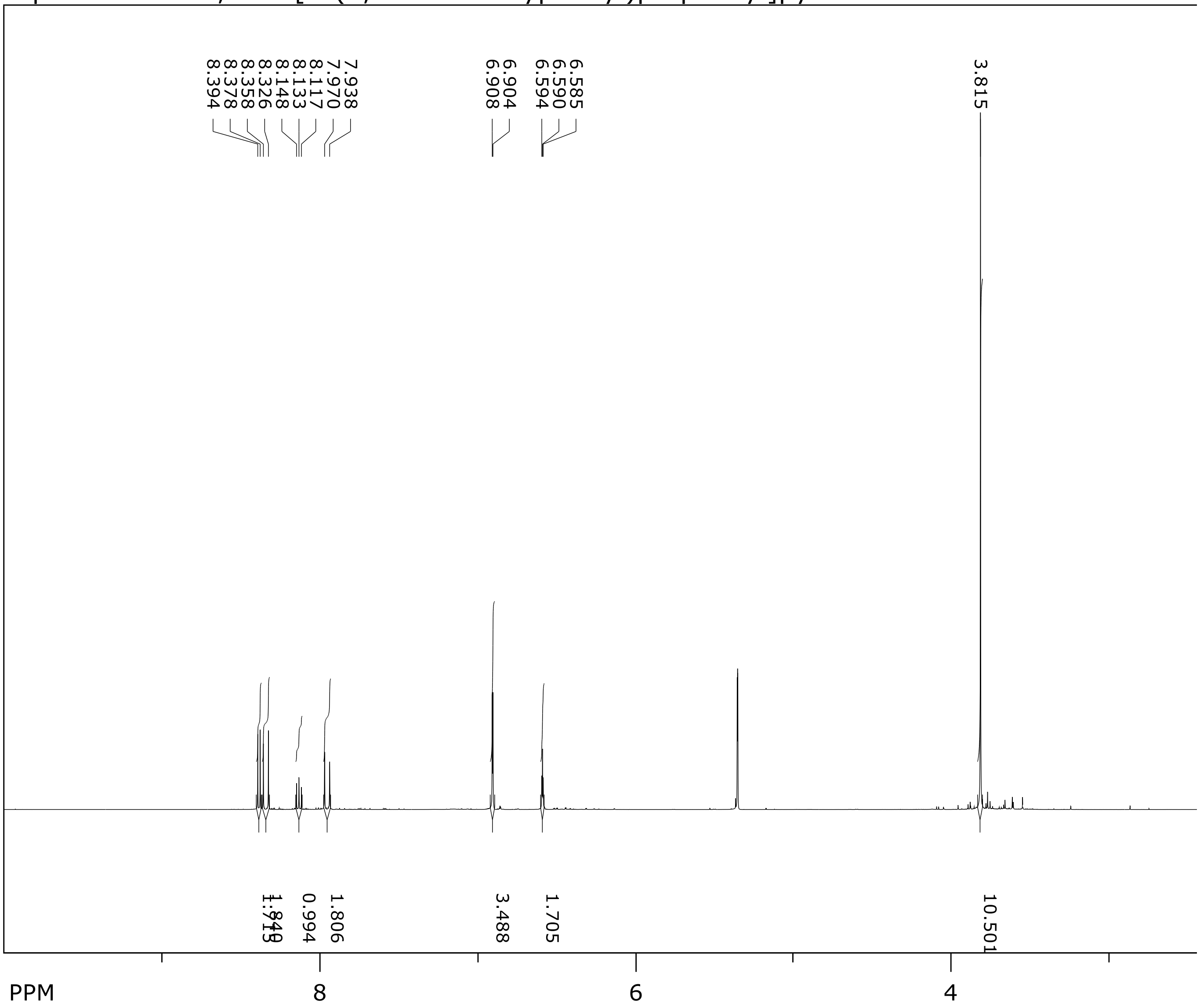

file: ... Data\EAV\500NMR\EAV-IV-68-2\1\fid expt: <zg30>
transmitter freq.: 500.063088 MHz
time domain size: 65536 points
width: 10330.58 Hz = 20.6586 ppm = 0.157632 Hz/pt
number of scans: 32

freq. of 0 ppm: 500.060000 MHz
processed size: 32768 complex points
LB: 0.300 GF: 0.0000

SpinWorks 4: 2,6-Di-[3-(3,5-dimethoxyphenyl)propenoyl]pyridine

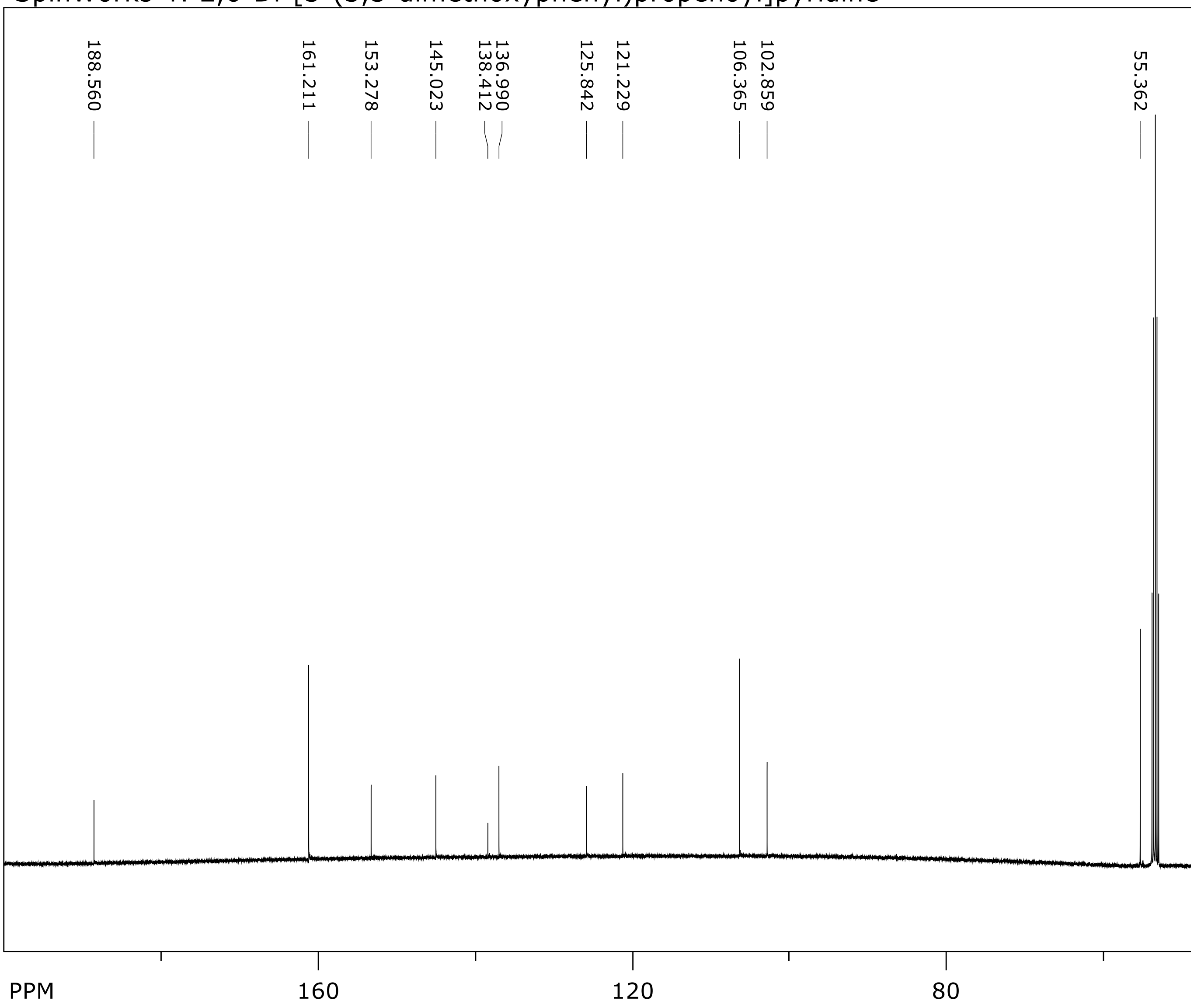

file: ...Data\EAV\500NMR\EAV-IV-68-2C\1\fid   expt: <zgpg>
transmitter freq.: 125.752761 MHz
time domain size: 262144 points
width: 26455.03 Hz = 210.3733 ppm = 0.100918 Hz/pt
number of scans: 1152

freq. of 0 ppm: 125.740187 MHz
processed size: 131072 complex points
LB: 0.300   GF: 0.0000

SpinWorks 4: 2,6-Di-[3-(3,4-methylenedioxyphenyl)propenoyl]pyridine

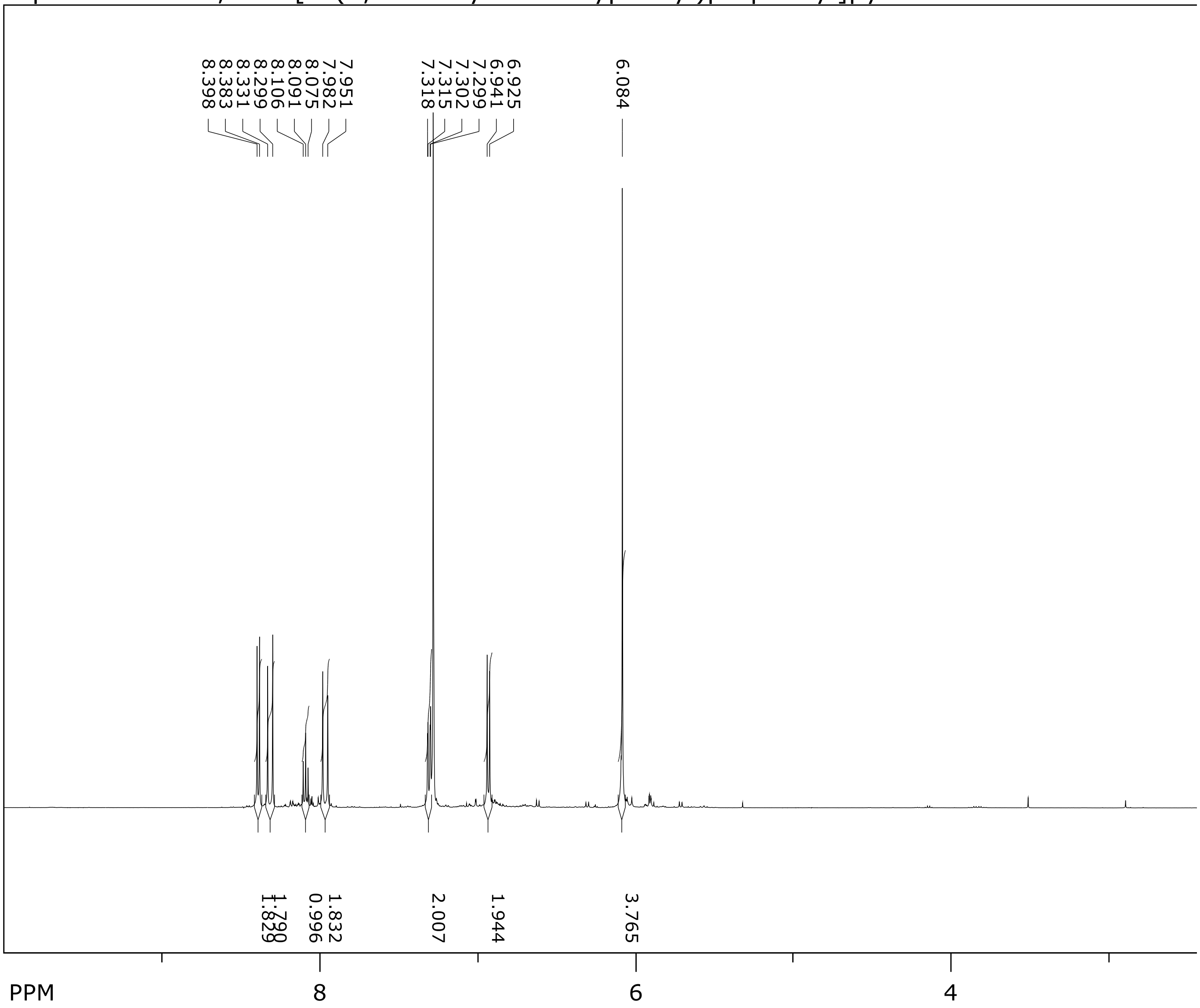

file: ...R Data\EAV\500NMR\EAV-VII-98\1\fid expt: <zg30>
transmitter freq.: 500.063088 MHz
time domain size: 65536 points
width: 10330.58 Hz = 20.6586 ppm = 0.157632 Hz/pt
number of scans: 64

freq. of 0 ppm: 500.060000 MHz
processed size: 32768 complex points
LB: 0.300 GF: 0.0000

SpinWorks 4: 2,6-Di-[3-(3,4-Methylenedioxyphenyl)propenoyl]pyridine

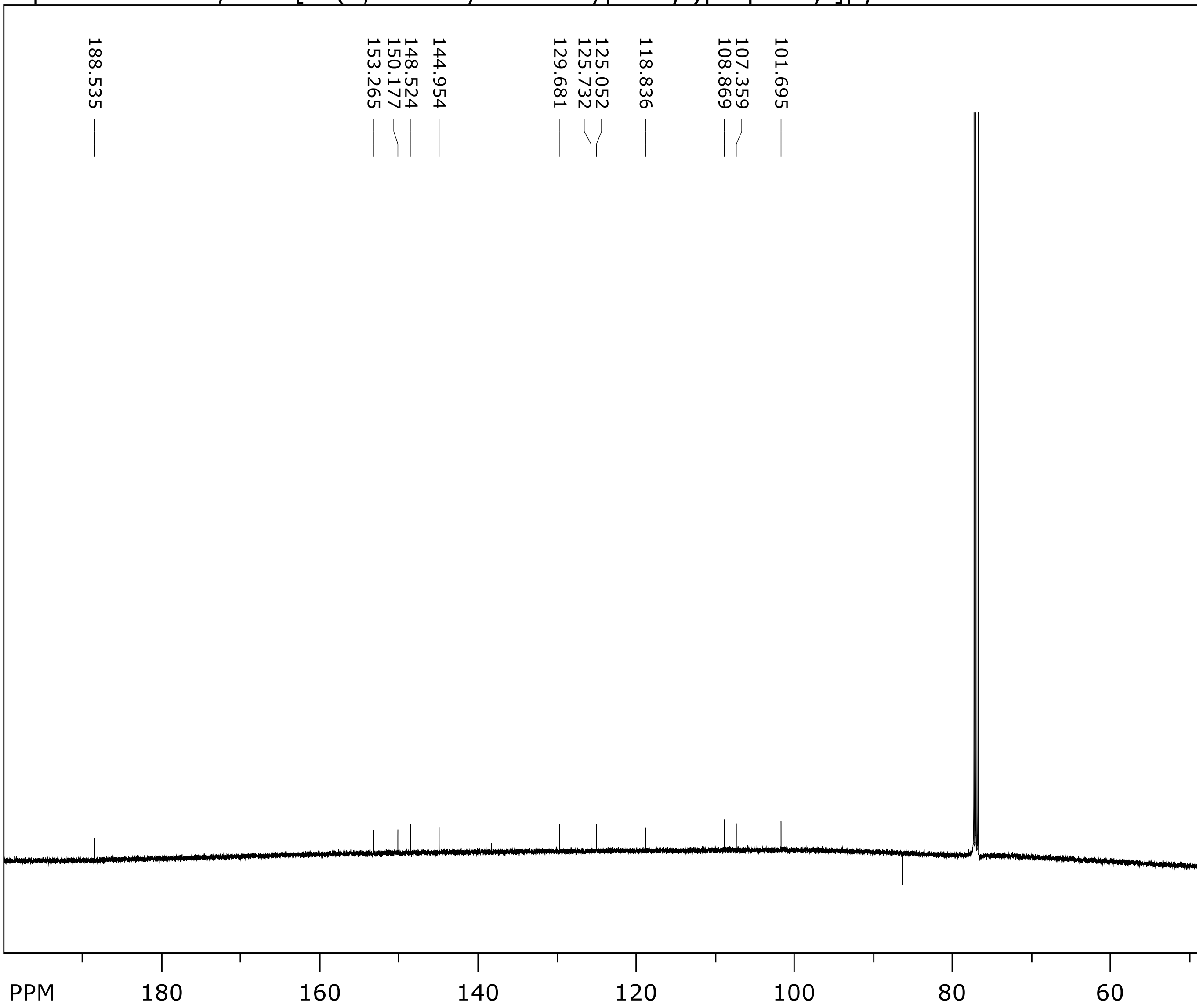

file: ... Data\EAV\500NMR\EAV-VII-98C\1\fid expt: <zgpg>
transmitter freq.: 125.752761 MHz
time domain size: 262144 points
width: 26455.03 Hz = 210.3733 ppm = 0.100918 Hz/pt
number of scans: 1152

freq. of 0 ppm: 125.740187 MHz
processed size: 131072 complex points
LB: 0.300 GF: 0.0000

SpinWorks 4: 2,6-Di-[3-(3,4,5-trimethoxyphenyl)propenoyl]pyridine

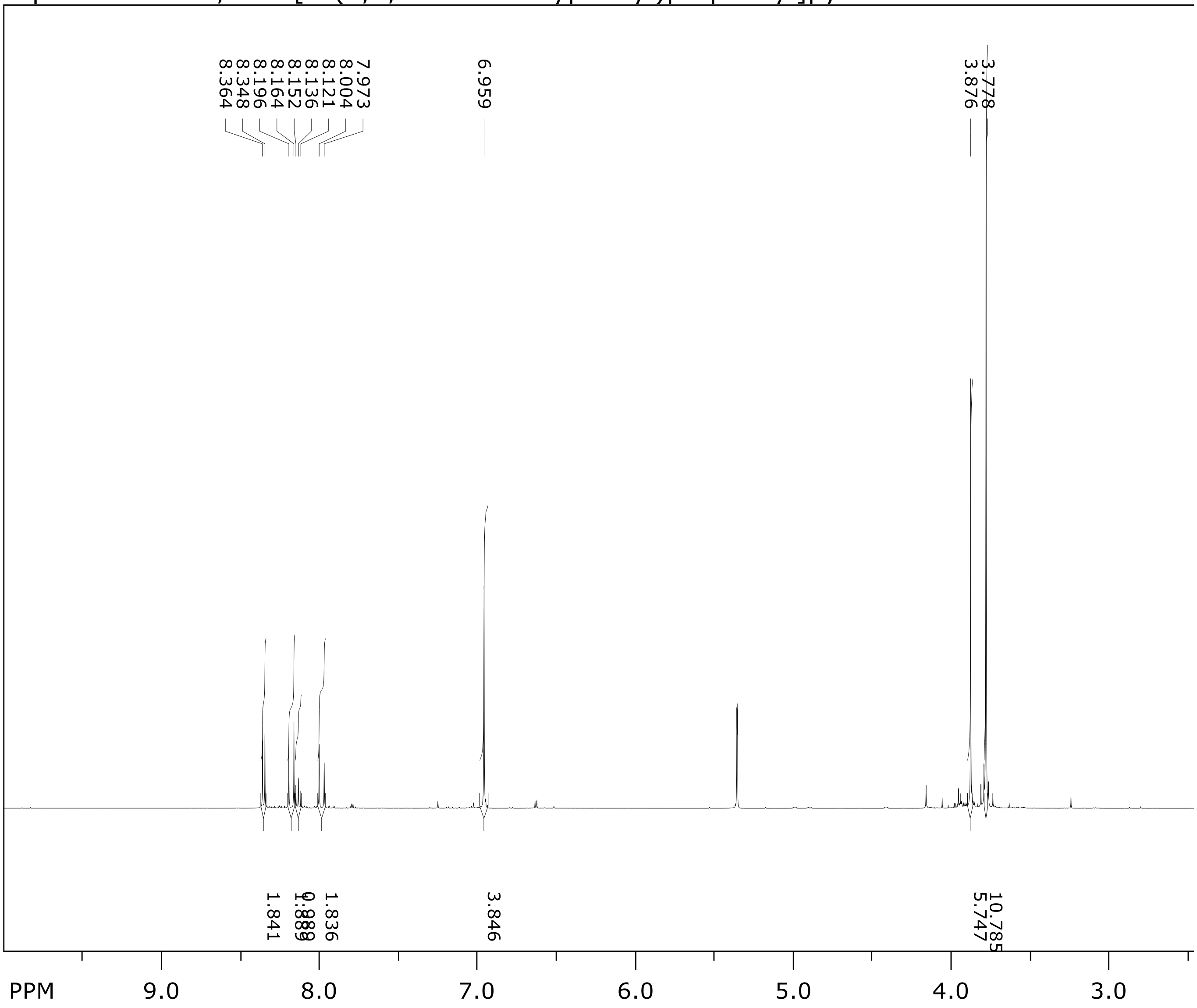

file: ...h\NMR Data\EAV\500NMR\LS-296\1\fid expt: <zg30>
transmitter freq.: 500.063088 MHz
time domain size: 65536 points
width: 10330.58 Hz = 20.6586 ppm = 0.157632 Hz/pt
number of scans: 64

freq. of 0 ppm: 500.060000 MHz
processed size: 32768 complex points
LB: 0.300 GF: 0.0000

SpinWorks 4: 2,6-Di-[3-(3,4,5-Trimethoxyphenyl)propenoyl]pyridine

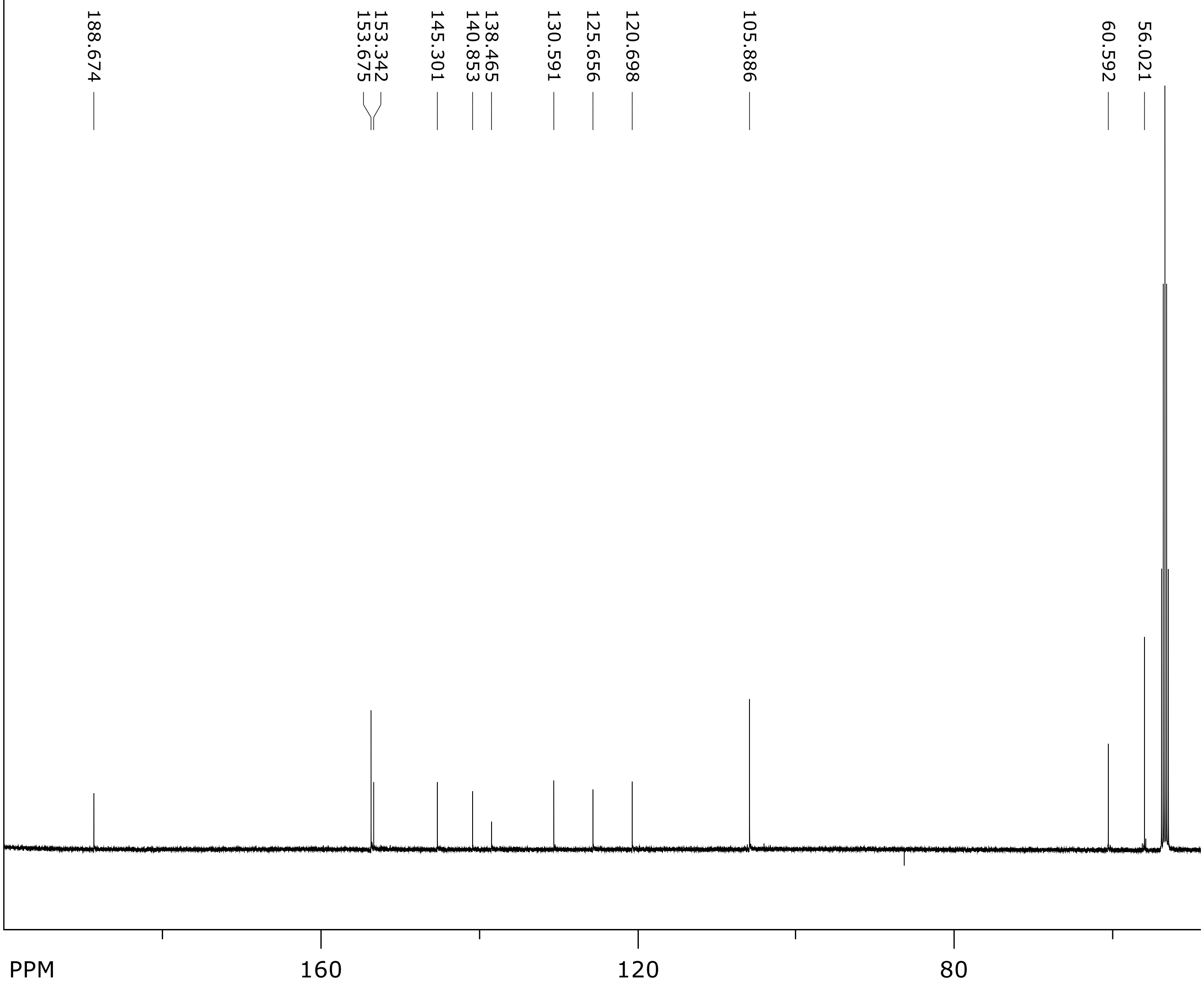

file: ...\NMR Data\EAV\500NMR\LS-296C\1\fid expt: <zgpg>
transmitter freq.: 125.752761 MHz
time domain size: 262144 points
width: 26455.03 Hz = 210.3733 ppm = 0.100918 Hz/pt
number of scans: 1152

freq. of 0 ppm: 125.740187 MHz
processed size: 131072 complex points
LB: 0.300 GF: 0.0000

SpinWorks 4: 2,6-Di-[3-(4-methoxymethoxyphenyl)propenoyl]pyridine

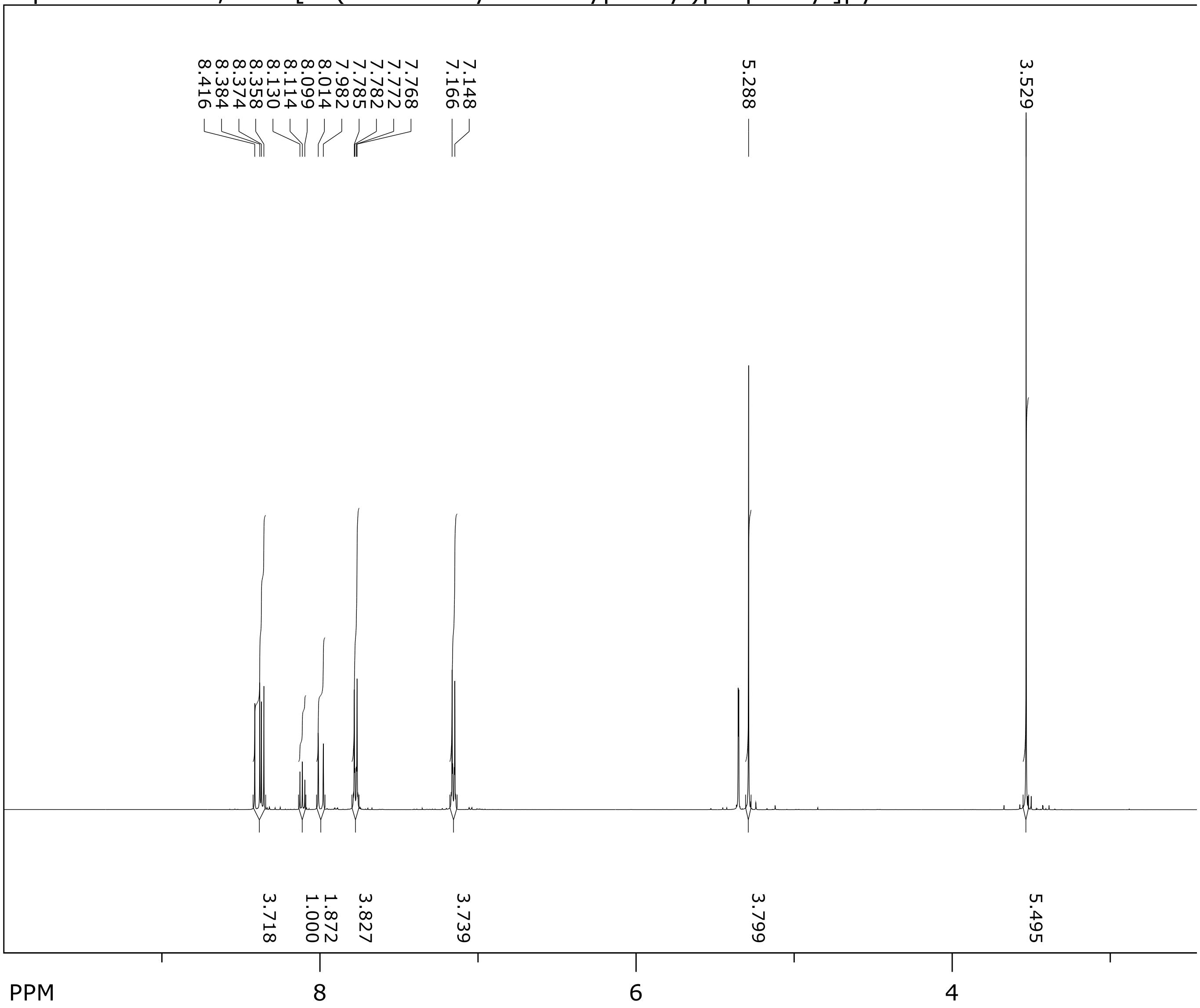

file: ...h\NMR Data\EAV\500NMR\LS-277\1\fid expt: <zg30>
transmitter freq.: 500.063088 MHz
time domain size: 65536 points
width: 10330.58 Hz = 20.6586 ppm = 0.157632 Hz/pt
number of scans: 64

freq. of 0 ppm: 500.060000 MHz
processed size: 32768 complex points
LB: 0.300 GF: 0.0000

SpinWorks 4: 2,6-Di-[3-(3-methoxy-4-methoxymethoxyphenyl)propenoyl]pyridine

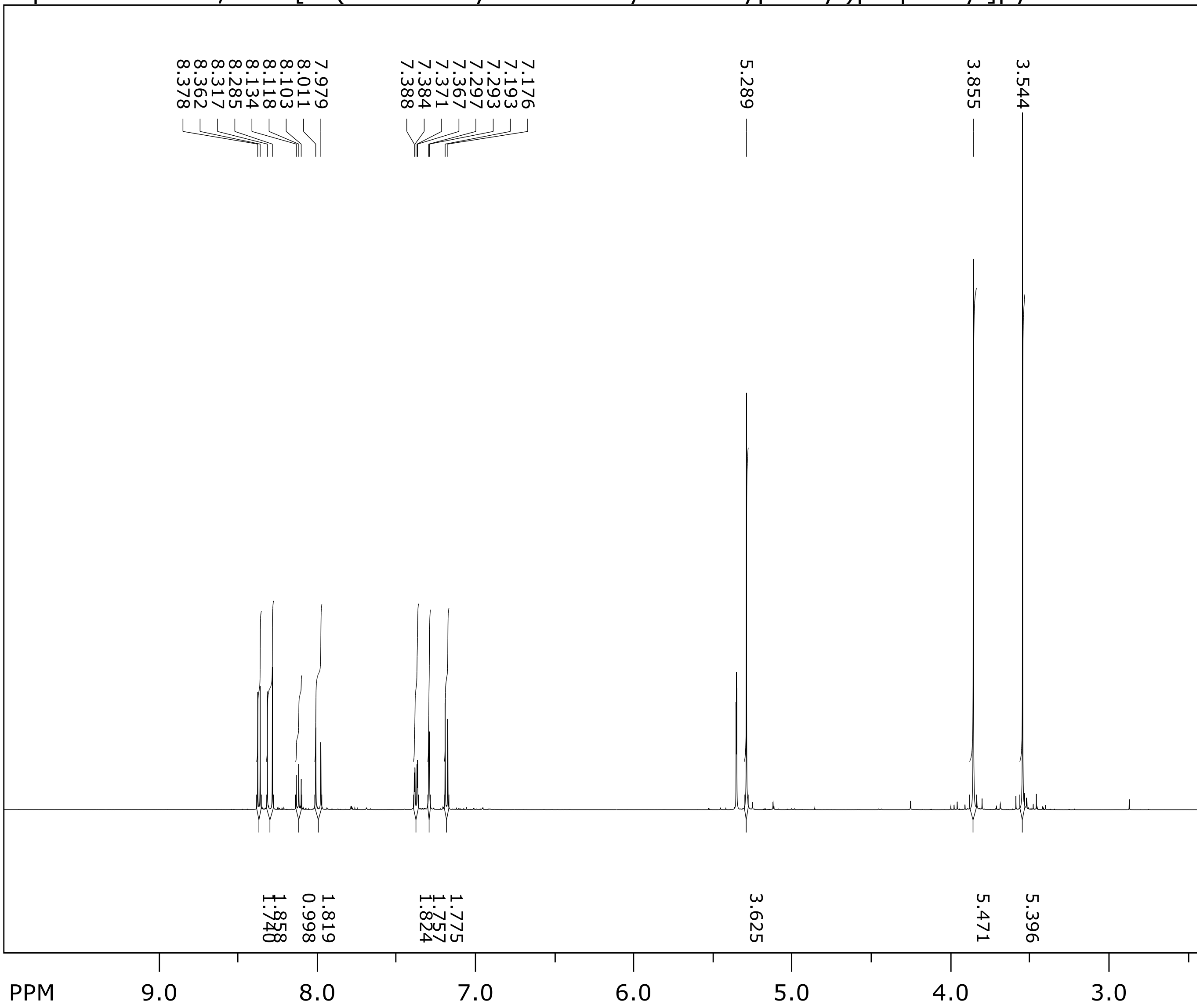

file: ...h\NMR Data\EAV\500NMR\LS-295\1\fid expt: <zg30>
transmitter freq.: 500.063088 MHz
time domain size: 65536 points
width: 10330.58 Hz = 20.6586 ppm = 0.157632 Hz/pt
number of scans: 32

freq. of 0 ppm: 500.060000 MHz
processed size: 32768 complex points
LB: 0.300 GF: 0.0000

SpinWorks 4: 2,6-Di-[3-(3-methoxy-4-methoxymethoxyphenyl)propenoyl]pyridine

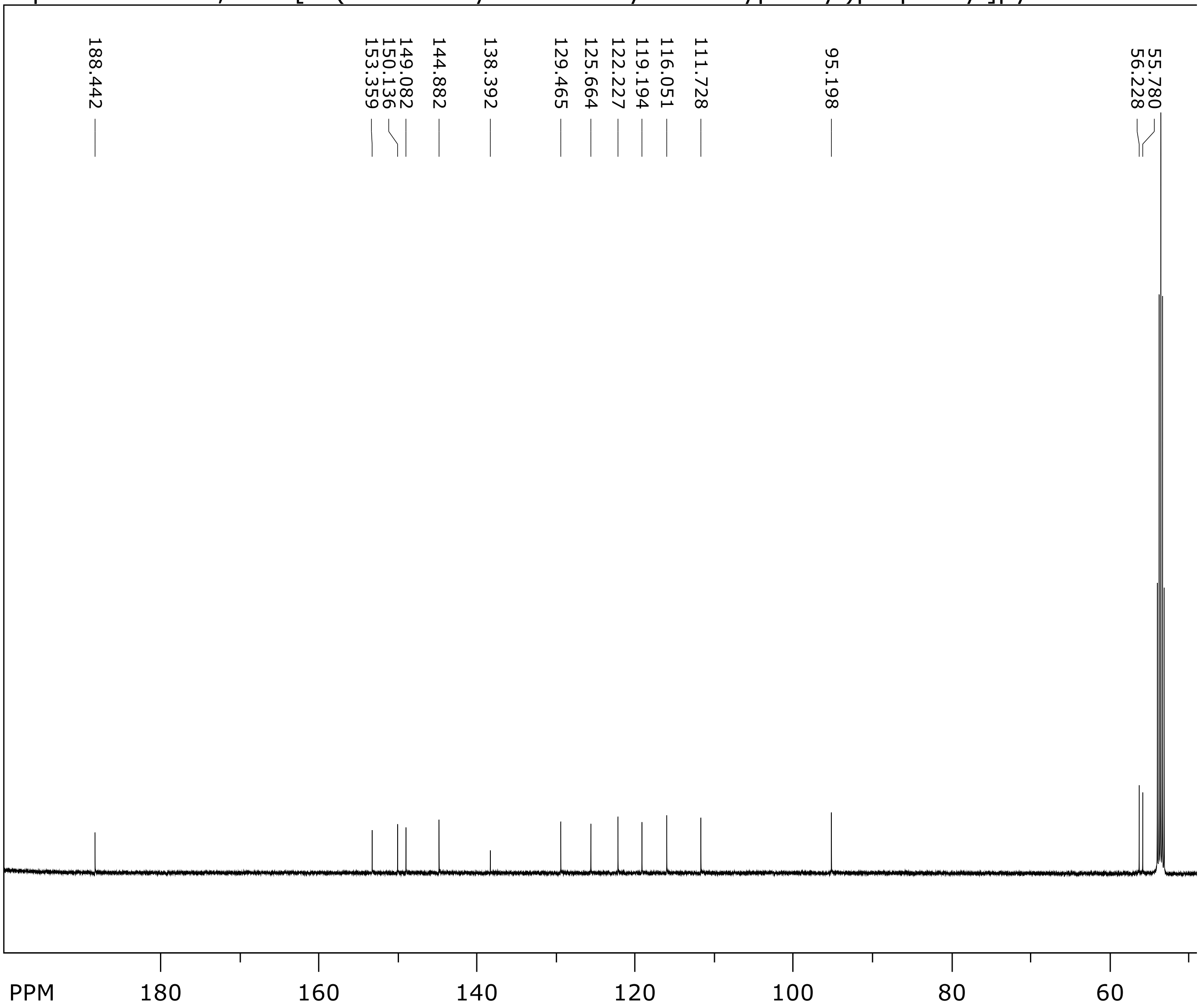

file: ...\NMR Data\EAV\500NMR\LS-295C\1\fid expt: <zgpg>
transmitter freq.: 125.752761 MHz
time domain size: 262144 points
width: 26455.03 Hz = 210.3733 ppm = 0.100918 Hz/pt
number of scans: 1408

freq. of 0 ppm: 125.740187 MHz
processed size: 131072 complex points
LB: 1.000 GF: 0.0000

SpinWorks 4: 2,6-Di-[3-(3-Benzyloxy-4-methoxyphenyl)propenoyl]pyridine

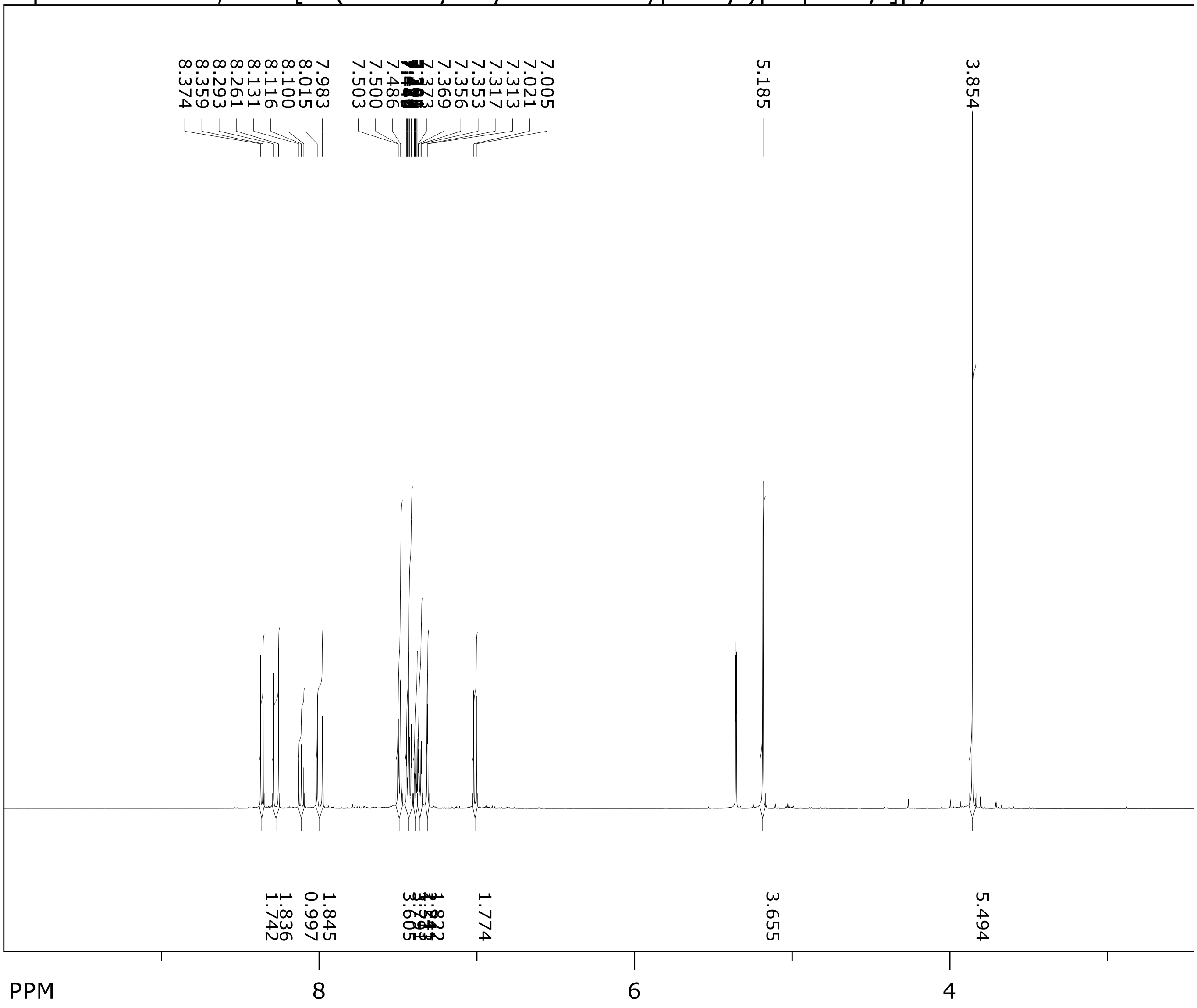

file: ...\NMR Data\EAV\500NMR\LS-192A\1\fid expt: <zg30>
transmitter freq.: 500.063088 MHz
time domain size: 65536 points
width: 10330.58 Hz = 20.6586 ppm = 0.157632 Hz/pt
number of scans: 64

freq. of 0 ppm: 500.060000 MHz
processed size: 32768 complex points
LB: 0.300 GF: 0.0000

SpinWorks 4: 2,6-Di-[3-(4-Benzyloxy-3-methoxyphenyl)propenoyl]pyridine

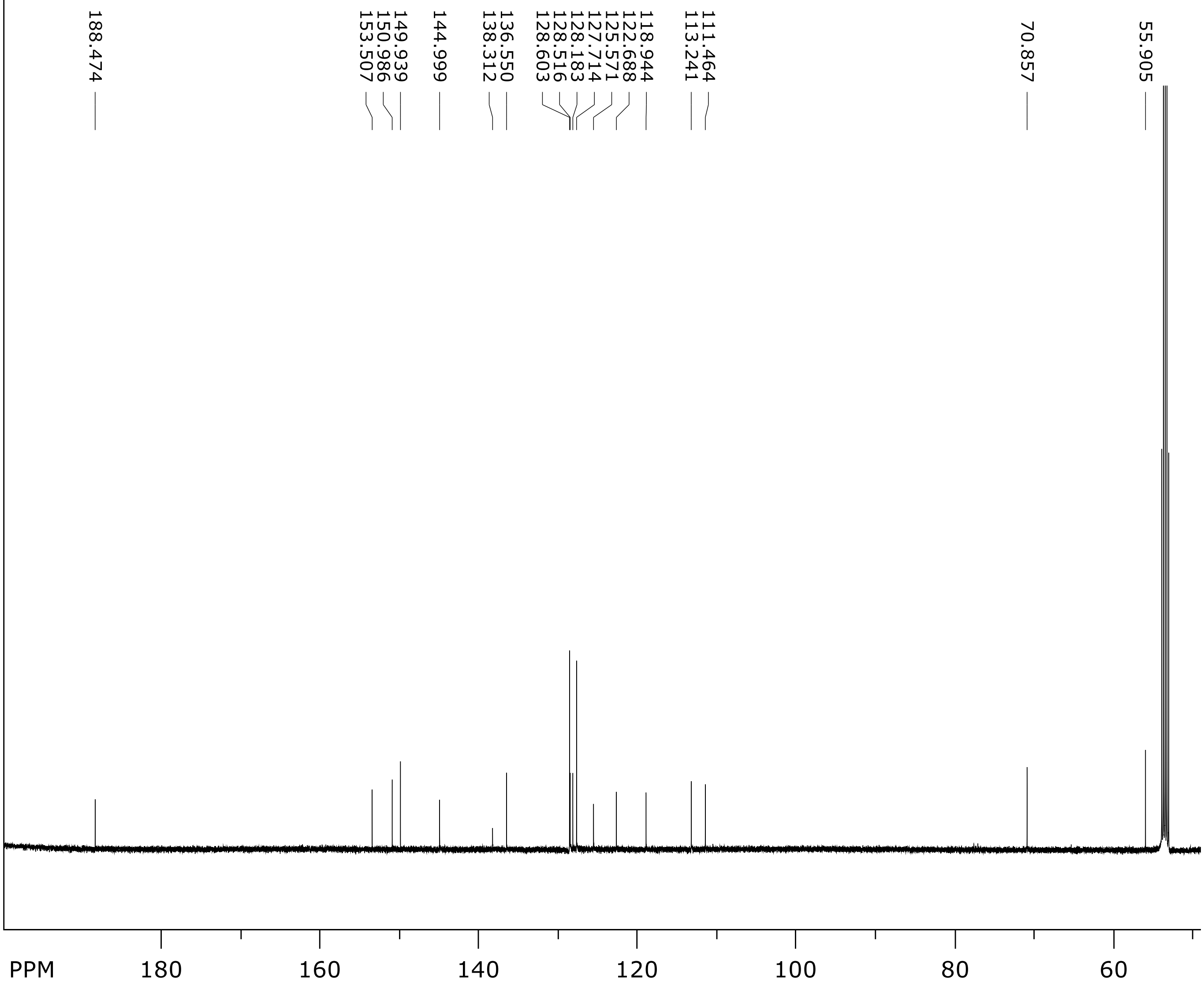

file: ...NMR Data\EAV\500NMR\LS-192CA\1\fid expt: <zgpg>
transmitter freq.: 125.752761 MHz
time domain size: 262144 points
width: 26455.03 Hz = 210.3733 ppm = 0.100918 Hz/pt
number of scans: 1800

freq. of 0 ppm: 125.740187 MHz
processed size: 131072 complex points
LB: 0.300 GF: 0.0000

SpinWorks 4: 2,6-Di-[5-(4-dimeyhylaminophenyl)penta-2,4-dienoyl]pyridine

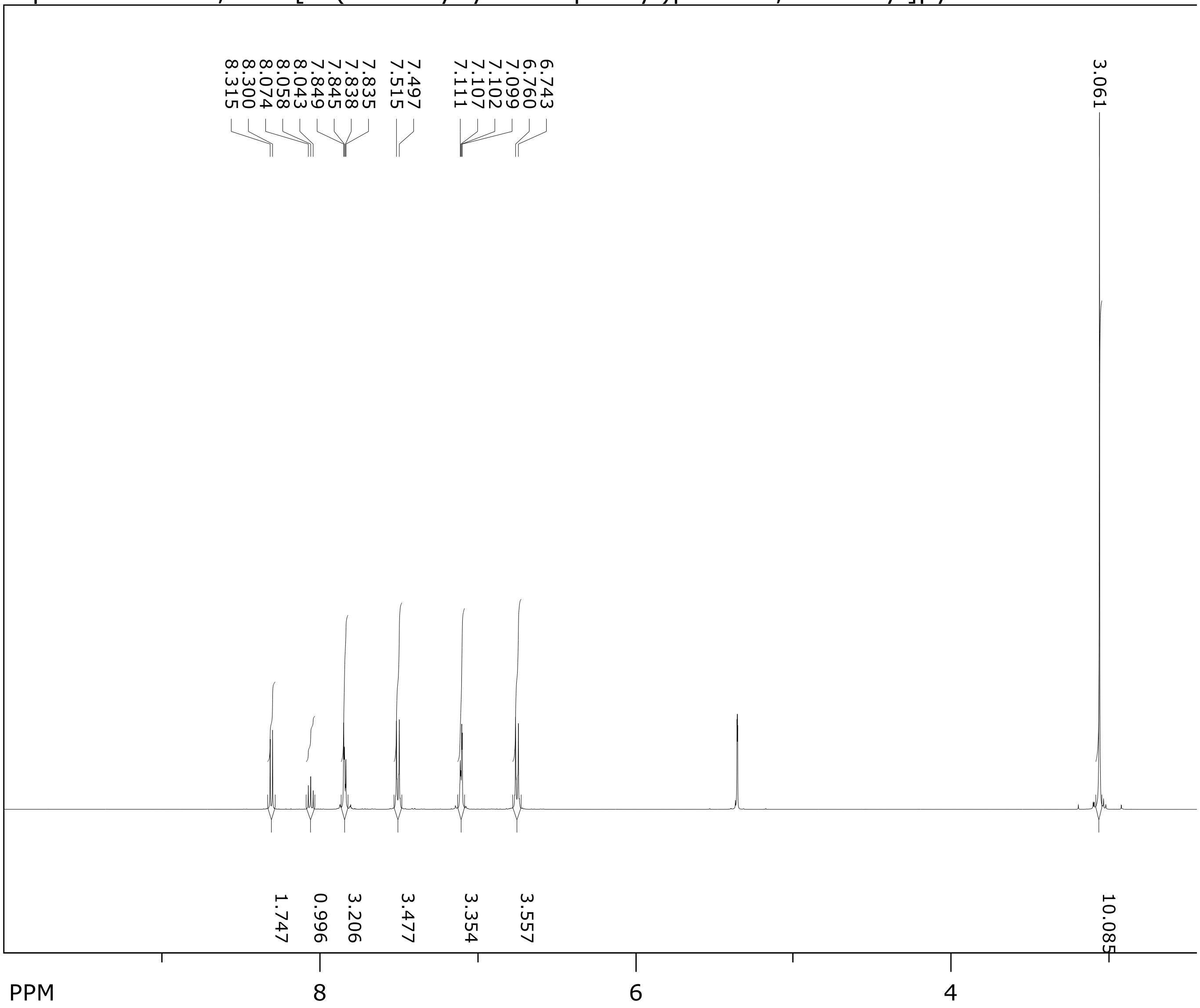

file: ...R Data\EAV\500NMR\EAV-VII-59\1\fid expt: <zg30>
transmitter freq.: 500.063088 MHz
time domain size: 65536 points
width: 10330.58 Hz = 20.6586 ppm = 0.157632 Hz/pt
number of scans: 64

freq. of 0 ppm: 500.060000 MHz
processed size: 32768 complex points
LB: 0.300 GF: 0.0000

SpinWorks 4: 2,6-Di-[5-(4-dimethylaminophenyl)pent-2,4-dienoyl]pyridine

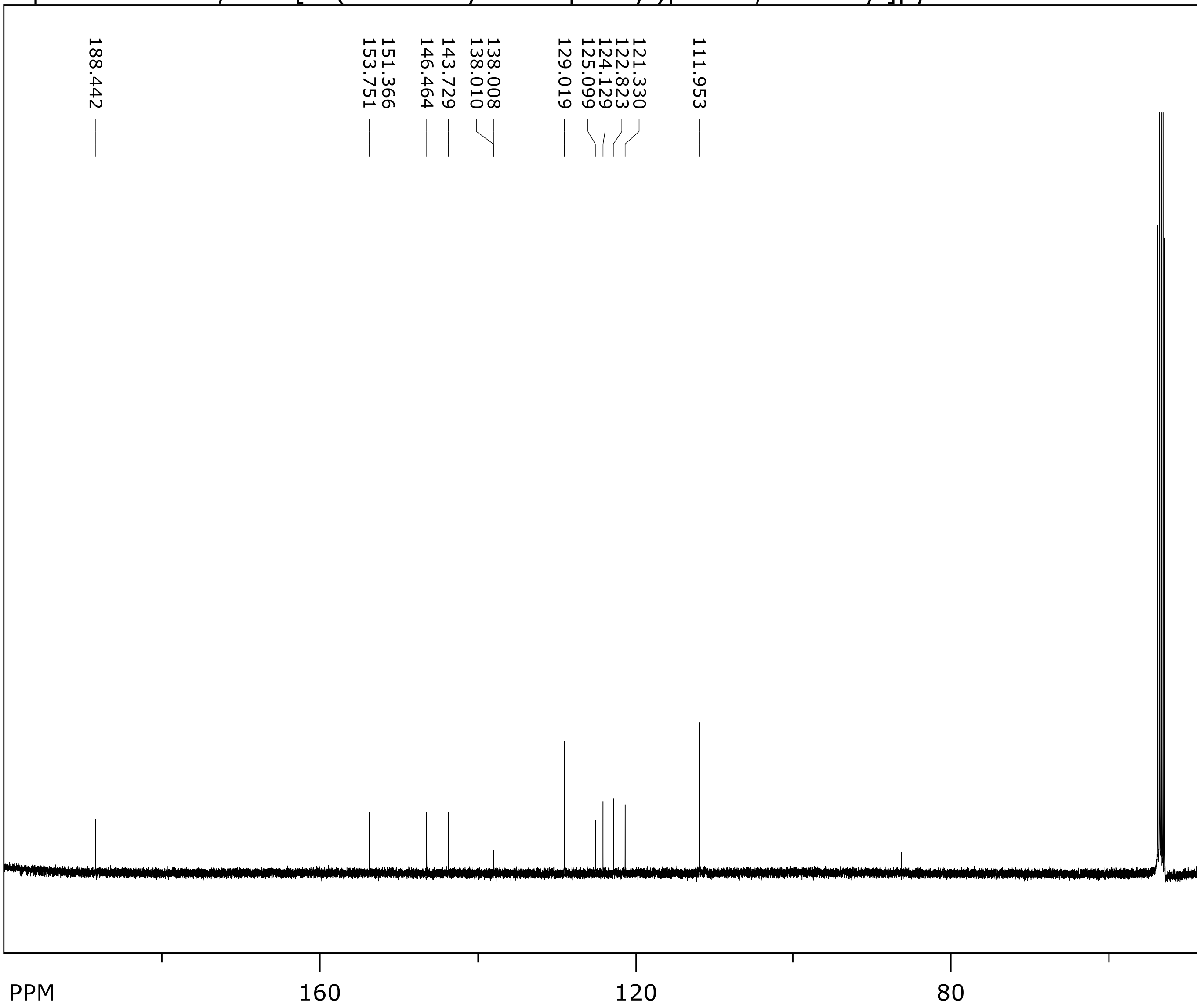

file: ... Data\EAV\500NMR\EAV-VII-59C\1\fid expt: <zgpg>
transmitter freq.: 125.752761 MHz
time domain size: 262144 points
width: 26455.03 Hz = 210.3733 ppm = 0.100918 Hz/pt
number of scans: 1416

freq. of 0 ppm: 125.740187 MHz
processed size: 131072 complex points
LB: 0.300 GF: 0.0000

SpinWorks 4: 2,6-Di-[3-(2,3,4-trimethoxyphenyl)propenoyl]pyridine

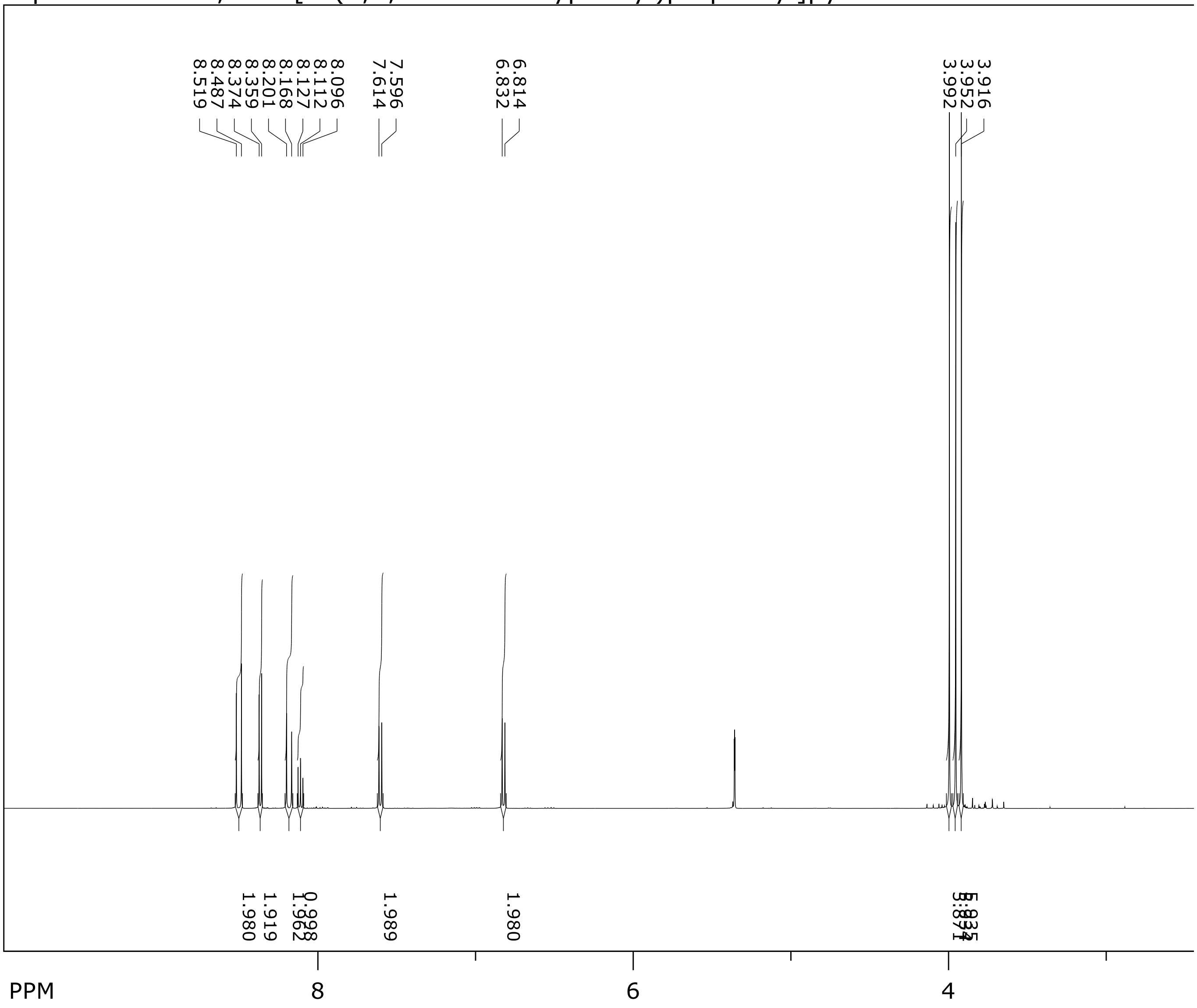

file: ...R Data\EAV\500NMR\EAV-VI-149\1\fid expt: <zg30>
transmitter freq.: 500.063088 MHz
time domain size: 65536 points
width: 7507.51 Hz = 15.0131 ppm = 0.114555 Hz/pt
number of scans: 64

freq. of 0 ppm: 500.060000 MHz
processed size: 32768 complex points
LB: 0.300 GF: 0.0000

SpinWorks 4: 2,6-Di-[3-(2,3,4-trimehoxyphenyl)propenoyl]pyridine

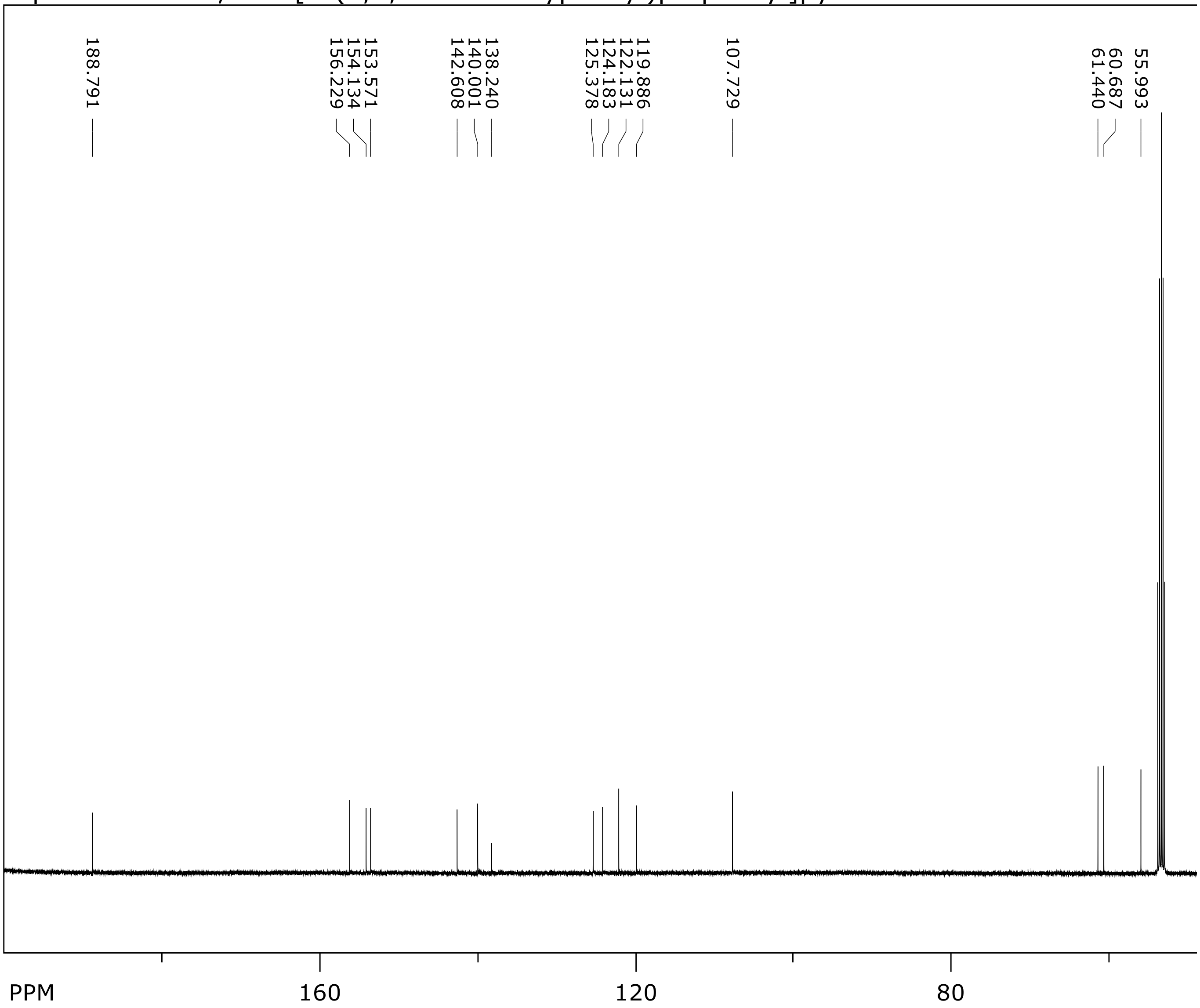

file: ... Data\EAV\500NMR\EAV-VI-149C\1\fid expt: <zgpg>
transmitter freq.: 125.752761 MHz
time domain size: 262144 points
width: 26455.03 Hz = 210.3733 ppm = 0.100918 Hz/pt
number of scans: 1800

freq. of 0 ppm: 125.740187 MHz
processed size: 131072 complex points
LB: 0.300 GF: 0.0000

SpinWorks 4: 2,6-Di-[3-(2,4,5-trimethoxyphenyl)propenoyl]pyridine

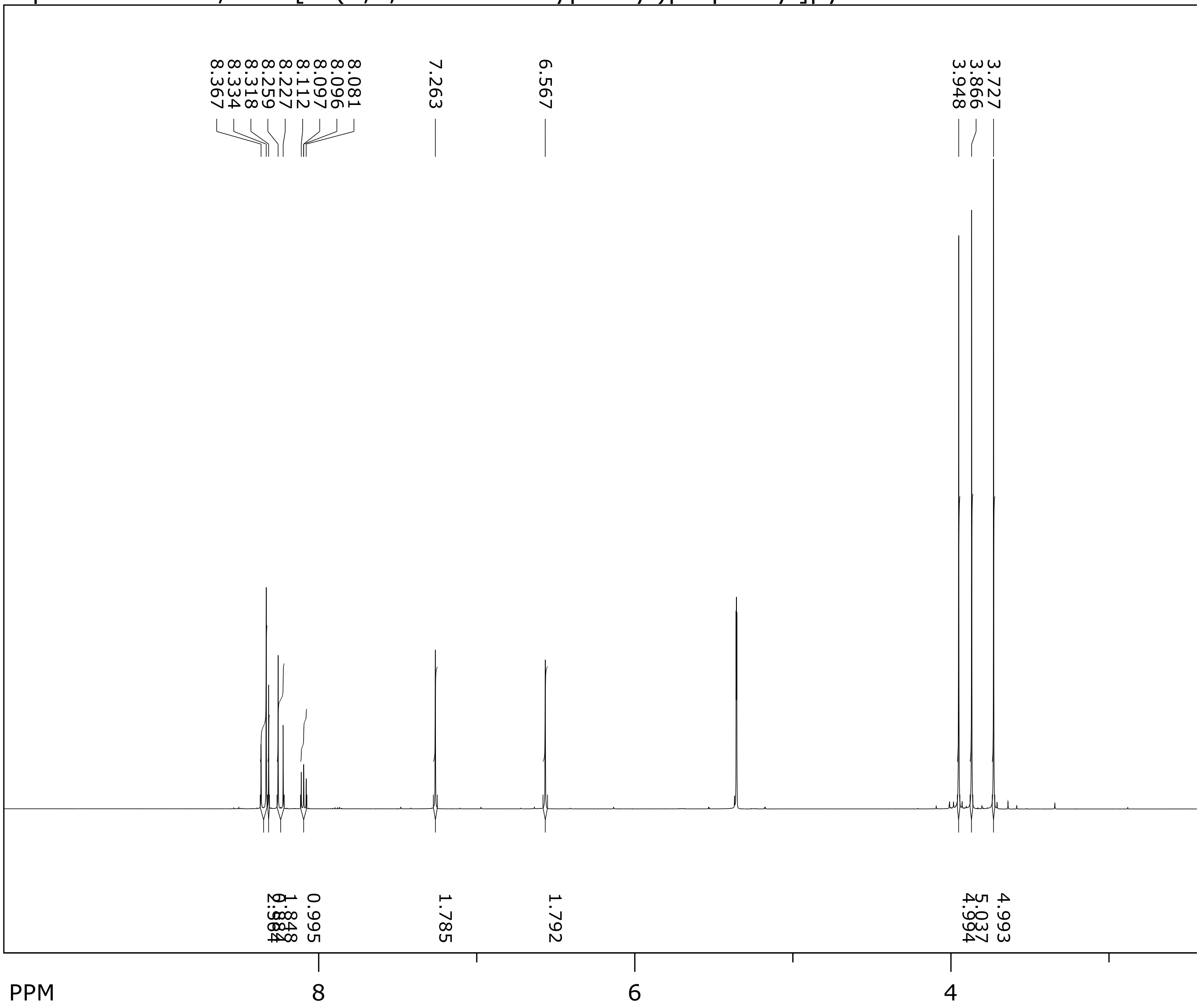

file: ...R Data\EAV\500NMR\EAV-VI-150\1\fid expt: <zg30>
transmitter freq.: 500.063088 MHz
time domain size: 65536 points
width: 7507.51 Hz = 15.0131 ppm = 0.114555 Hz/pt
number of scans: 64

freq. of 0 ppm: 500.060000 MHz
processed size: 32768 complex points
LB: 0.300 GF: 0.0000

SpinWorks 4: 2,6-Di-[3-(2,4,5-trimehoxyphenyl)propenoyl]pyridine

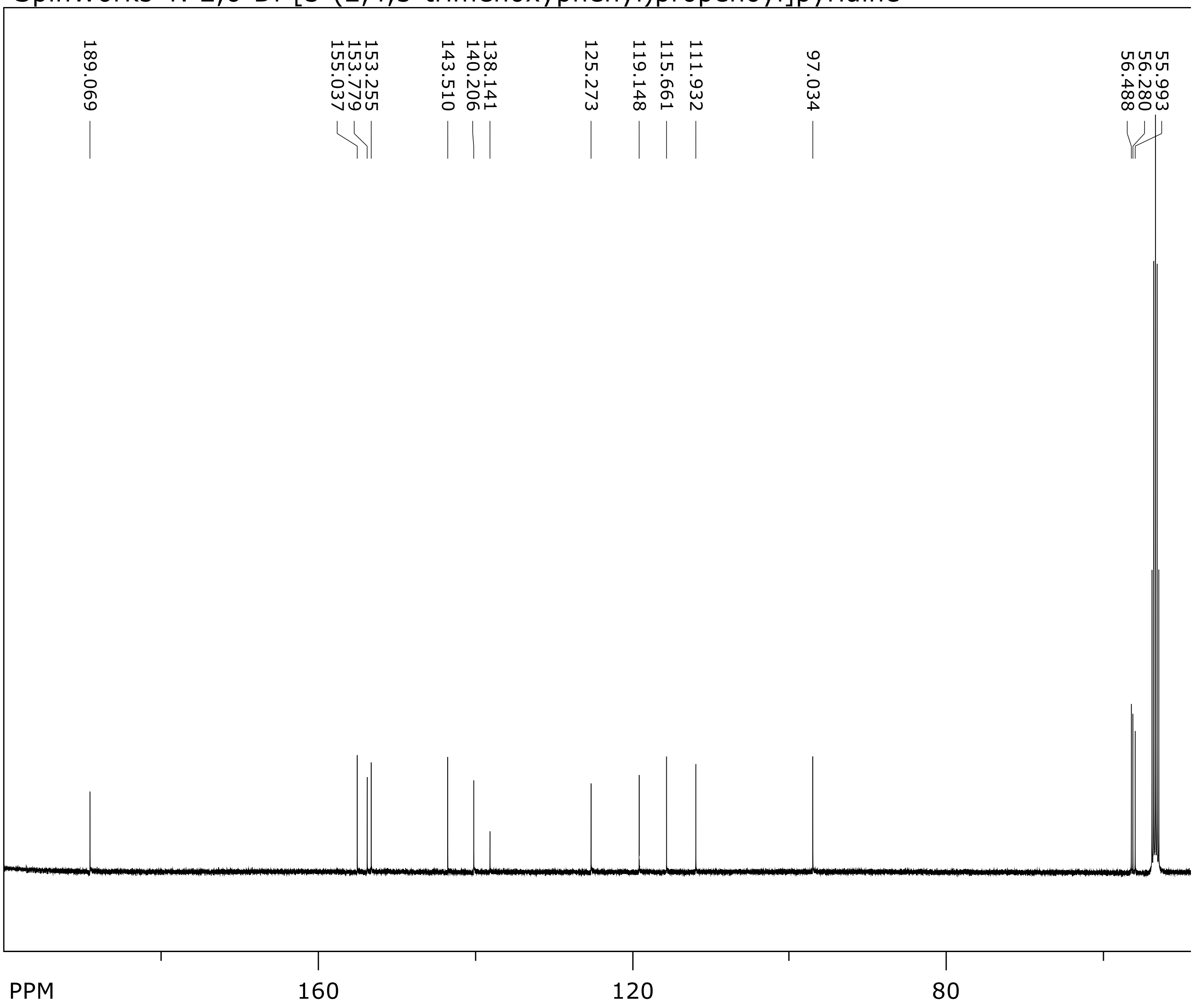

file: ...Data\EAV\500NMR\EAV-VI-150C1\1\fid   expt: <zgpg>
transmitter freq.: 125.752761 MHz
time domain size: 262144 points
width: 26455.03 Hz = 210.3733 ppm = 0.100918 Hz/pt
number of scans: 1800

freq. of 0 ppm: 125.740187 MHz
processed size: 131072 complex points
LB: 0.300   GF: 0.0000

SpinWorks 4: 2,6-Di-[3-(4-hydroxy-3-methoxyphenyl)propenoyl]pyridine

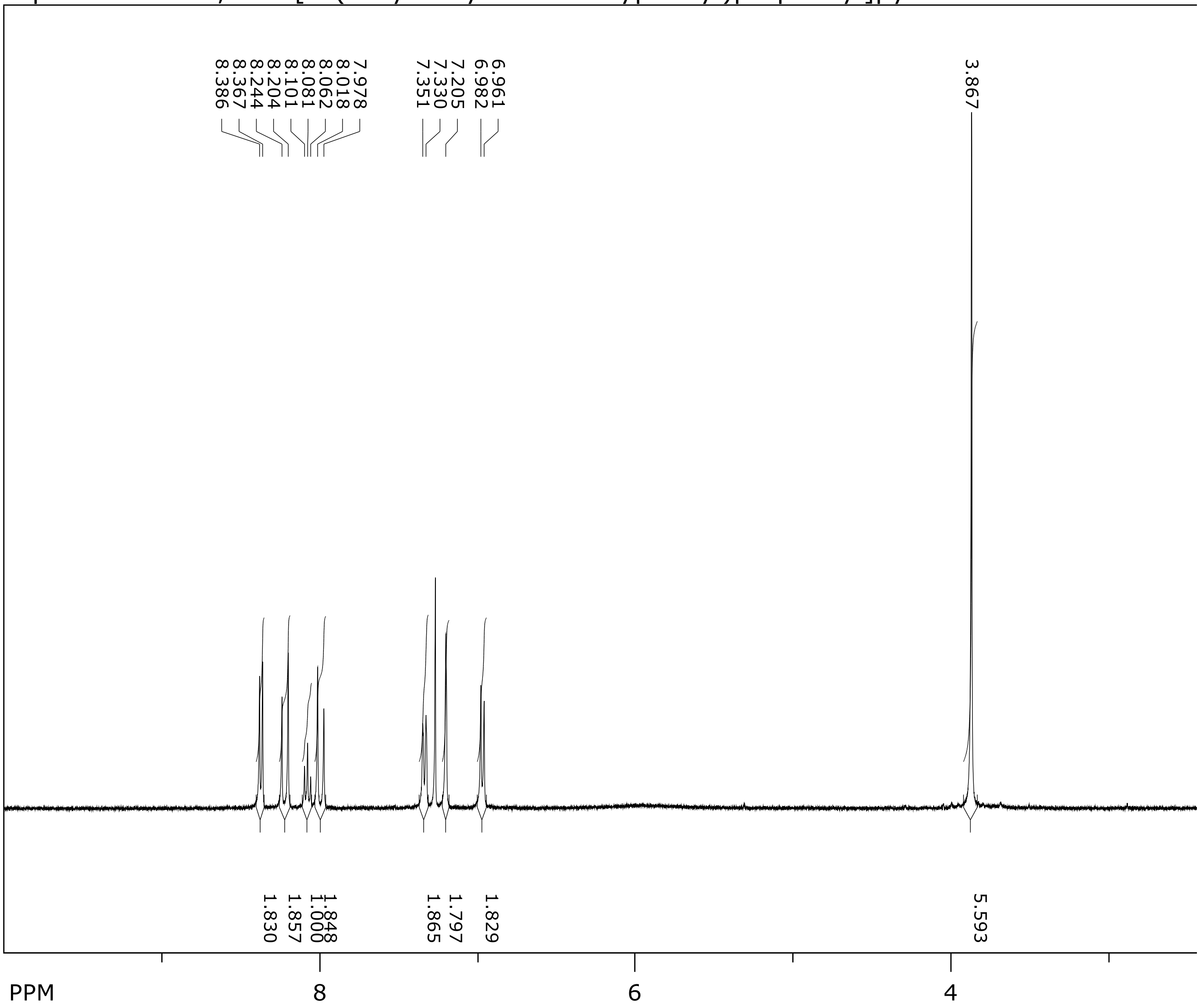

file: ...0NMR\nmr\LS 297 after column\1\fid expt: <zg30>
transmitter freq.: 399.995470 MHz
time domain size: 65536 points
width: 8278.15 Hz = 20.6956 ppm = 0.126314 Hz/pt
number of scans: 32

freq. of 0 ppm: 399.993000 MHz
processed size: 32768 complex points
LB: 0.000 GF: 0.0000

SpinWorks 4: 2,6-Di-[3-(4-hydroxy-3-methoxyphenyl)propenoyl]pyridine

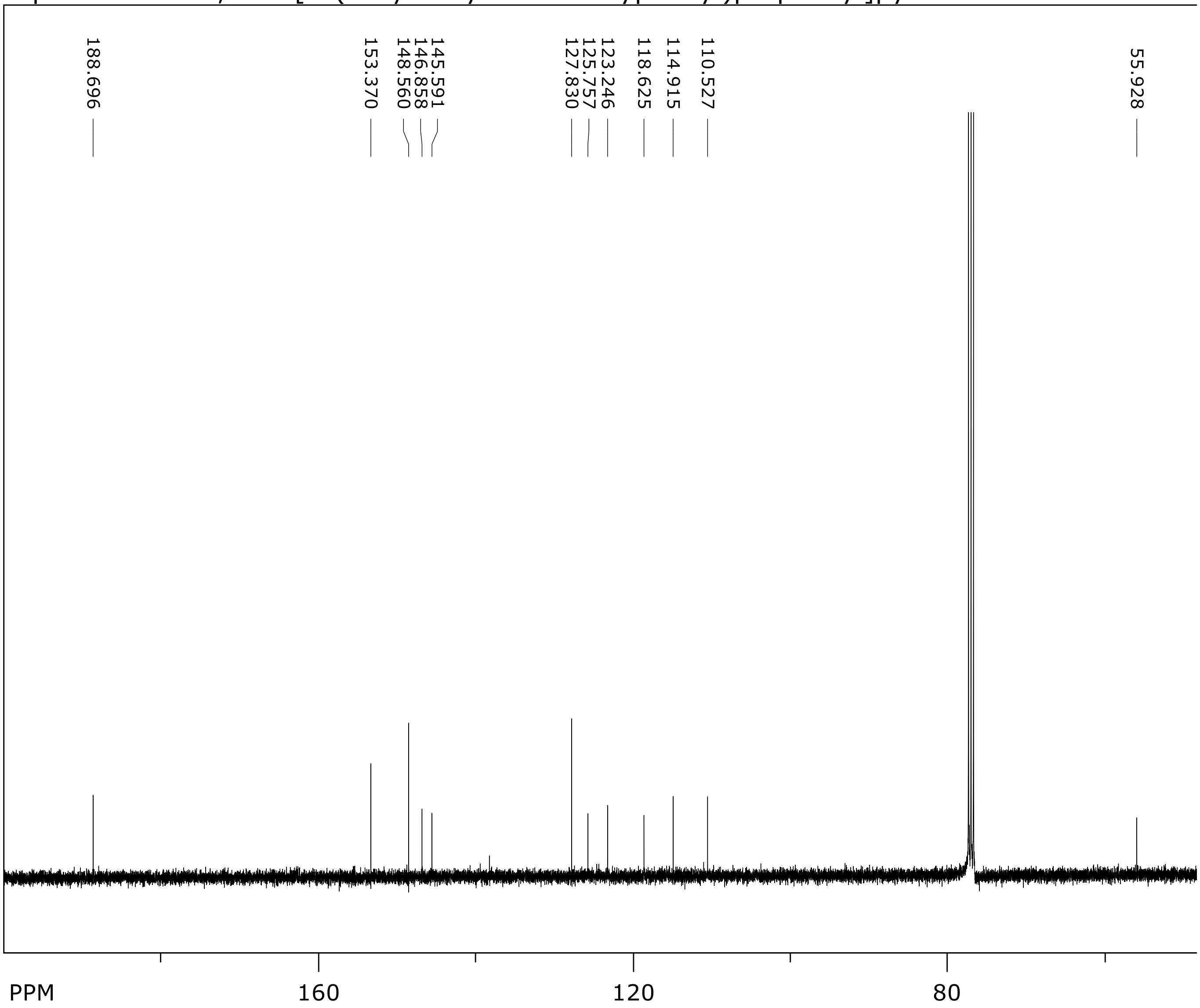

file: ...st. 1 and filtering in CDCl3\2\fid   expt: <zgpg30>
transmitter freq.: 100.588378 MHz
time domain size: 65536 points
width: 23980.82 Hz = 238.4054 ppm = 0.365918 Hz/pt
number of scans: 4096

freq. of 0 ppm: 100.578320 MHz
processed size: 32768 complex points
LB: 0.000   GF: 0.0000
